# Supplementary material for: Effect of upper limb isometric training (ULIT) on hamstring strength in early postoperative anterior cruciate ligament reconstruction patients: Study protocol for a randomized controlled trial
Source: PLoS One. 2025 Aug 21;20(8):e0319724. doi: 10.1371/journal.pone.0319724 (PMC12370102; doi:10.1371/journal.pone.0319724)
Supplement: S1 Appendix — (PDF) [file pone.0319724.s001.pdf]

S1 Appendix. Study Protocol for Ethics Application

EFFECT OF UPPER LIMB ISOMETRIC TRAINING ON HAMSTRING STRENGTH IN EARLY POSTOPERATIVE ANTERIOR CRUCIATE LIGAMENT RECONSTRUCTION PATIENTS: A RANDOMISED CONTROLLED TRIAL

Abstract

This study aims to investigate the effects of upper limb isometric training (ULIT) combined with standard care of an anterior cruciate ligament reconstruction (ACLR) rehabilitation program on hamstring strength and physical function in the early-phase postoperative period. Early recovery of hamstring strength is critical for knee stability and functional rehabilitation post-ACL surgery. Hamstring strength deficits are prolonged issues in rehabilitation, with significant hamstring strength deficiencies in an early postoperative ACLR, especially using hamstring autograft, leading to functional limitations and an increased risk of secondary injuries. High knee muscle strength imbalances can hinder strength return in later stages. However, swelling, donor site pain, joint stiffness, and graft protection often neglect the direct activation of the hamstring in the early period. Also, clinical practice guidelines and consensus on early rehabilitation strategies are conflicting. While lower limb strengthening exercises are the mainstay of rehabilitation, this study explores the potential benefits of ULIT in promoting hamstring activation through the inter-limb posterior myofascial kinetic chain. This study hypothesise that participants receiving ULIT in addition to standard care will demonstrate better hamstring strength at 12 weeks post-operatively compared to those receiving standard care rehabilitation alone. A total of 32 patients who underwent ACLR with hamstring tendon autografts will be randomly assigned to either the ULIT group or a control group. The ULIT group performed upper limb isometric exercises in addition to standard postoperative rehabilitation, while the control group followed the standard rehabilitation protocol alone. We will measure hamstring and quadriceps strength using hand-held dynamometry at baseline (preoperative), 4, 8, and 12 weeks postoperatively. The primary outcome is the change in hamstring strength in the operated limb, while secondary outcomes included hamstring flexibility and patient-reported outcomes on symptoms, physical function and adherence. Statistical analysis will be conducted to compare the strength recovery between the two groups. It is believed that the results of this study will enhance knowledge about the global functional muscle concept, offer effective, safe, inexpensive, and feasible interventions, and promote further research on the interlimb connection of muscle activation and flexibility in heterologous muscle groups after ACLR.

Keywords: isometric training, hamstring, anterior cruciate ligament, rehabilitation.

Background and Literature Review

Anterior Cruciate Ligament (ACL) injuries are common in sports, with a prevalence of 68.6 per 100,000 individuals per year (Matar et al., 2021). ACL reconstruction (ACLR) is indicated for those who are physically active and experience prolonged knee instability (Hiemstra et al., 2000). Over 100,000 ACLRs were performed worldwide, with an annual increase of at least 2% in the past decade (Zbrojkiewicz et al., 2018). The ACLR significantly impacts physical function and keeps the patients out of sports for at least six months (Kuenze et al., 2023). The prevalence of knee weakness, tightness, and physical function limitation in post-ACLR patients have been associated with demographic factors, duration from injury to surgery, concomitant bone and chondral lesion, type of graft and surgical technique, and timing and structure of rehabilitation program (Fairus et al., 2022; Herbawi et al., 2022; Roula et al., 2023). The ACLR may result in chronic neuromuscular inhibition and persistent hamstring strength deficits, especially when using an ipsilateral hamstring autograft (San Jose et al., 2023). Around 50% hamstring strength deficit was observed in patients with hamstring tendon graft at 4 weeks, and only 46% achieved a knee flexor limb symmetry index (LSI) >90% at 6 months, compared to 67% with patellar tendon graft (Forelli et al., 2023; Zumstein et al., 2022). Knee flexor weakness deficits can be significant during the return to sport (RTS) phase at 6 months and even over a year following the ACLR (Lee & Lee, 2020). Chronic hamstring strength deficiencies following ACLR increase the risk of ACL re-injury, changes in running mechanics, negative self-reported outcomes, and a higher likelihood of post-traumatic osteoarthritis (Herbawi et al., 2022; Högberg et al., 2024; San Jose et al., 2023). Despite a significant increase in ACLR, no recent studies have reported whether the prevalence of ACLR cases among the Malaysian population aligns with those reported in current literature worldwide.

Restoring knee flexor muscle function is crucial for ACL rehabilitation, as the hamstring muscles contribute to knee joint stability by preventing excessive ACL shear stresses (Ko et al., 2012; San Jose et al., 2023). Anatomically, the medial hamstrings reduce medial condyle liftoff and dynamic knee valgus, known risk factors for ACL injuries. Knee flexor weakness increases the chance of ACL re-injury (Rob et al., 2018). Impaired knee flexor function following ACLR using a hamstring graft is often caused by complications related to the donor site (San Jose et al., 2023). A severe grade 4 injury of the harvested semitendinosus tendon can lead to decreased hamstring muscle activation during eccentric contraction (Forelli et al., 2023; Kositsky et al., 2022). Regeneration of the tendon may take around 18 months in 10-50% of patients (Lee & Lee, 2020). If these deficiencies are not counterbalanced early by hamstring hypertrophy and activation, they can result in a chronic deficiency in knee flexion strength (Fukunaga et al., 2019). Additionally, the absence of compensatory hypertrophy in the medial hamstring muscles can result in deficiencies in the overall volume of the hamstring muscles, impacting knee control and worsening the imbalance in muscular strength around the knee (Labanca et al., 2022).

Early-phase rehabilitation from the first week up to 3 months (see Figure 1a), postoperative aims to alleviate pain, reduce swelling, restore knee joint range of motion and optimize muscle strength, regain daily activities like walking without crutches, and minimize

muscle atrophy (Roula et al., 2023). There is typically a 50% decrease in knee flexor isometric strength after 4 weeks (Harput et al., 2015). This decrease in strength often aligns with the transition from the early to mid-rehabilitation phase. At three months post ACLR, recovery in quadriceps strength was 82% and recovery in hamstring strength was 76% following a structured neuromuscular rehabilitation (Harput et al., 2015; McPherson et al., 2023). A high prevalence of knee muscle strength imbalances would impede the return of strength throughout the latter stages of rehabilitation (Ohji et al., 2022). However, direct activation and stretching of the hamstring in the early period, especially the first 12 weeks after ACLR rehabilitation, is often neglected and challenging because of swelling, donor site pain, joint stiffness and the necessity to protect the graft from excessive tensile force and joint loading (Cristiani et al., 2021). In post-operative ACLR, a neurological response known as Arthrogenic Muscular Inhibition (AMI) disrupts sensory information transmission, leading to dysfunction in the somatosensory and motor cortex (Pietrosimone et al., 2022). This inhibits quadriceps and hamstring activation due to a cortical reflex (Pinto et al., 2017; Sonnery-Cottet et al., 2022). Instead, there are limited clinical practice guidelines (CPG) and consensus on hamstring strength and flexibility rehabilitation strategy in the early stage following ACLR (see Figure 1a), with most of the studies in ACL prevention and rehabilitation focusing on gaining quadriceps strength and achieving full knee extension, which overshadows the need for strengthening and flexibility of hamstring (Roula et al., 2023; Svantesson et al., 2020). Despite that, restoring optimal hamstring strength is an important facet of functional recovery following ACLR, providing dynamic joint stabilization and preventing excessive ACL shear forces during forceful dynamic movement (San Jose et al., 2023). Thus, it is necessary to achieve optimal hamstring strength during the initial stage of the ACLR rehabilitation programme before progressing to the next phase.

The myofascial kinetic chain is a crucial part of functional anatomy, consisting of interconnected muscles and connective tissues (Myers, 2014; Stecco et al., 2023). The ULIT like shoulder abduction, external rotation, or scapular retraction can work out muscles in the posterior myofascial kinetic chain (PMKC), including the hamstrings (Deng et al., 2022; Nuhmani & Zouhal, 2022). This approach enhances neuromuscular coordination and movement efficiency, integrating hamstring muscles into functional activities and dynamic motions. The ULIT indirectly loads the hamstring muscles through their connections along the kinetic chain, enhancing muscle activation, joint stability, and movement efficiency, rendering isometric training a potentially efficacious rehabilitation intervention.

To maximize the strength benefits of inter-limb cross effect, it is recommended to train with a resistance level of at least 50% of one's maximum voluntary strength, as this intensity has been proven to yield significant strength gains (Smyth et al., 2023). The ULIT has the potential to serve as a warm-up before engaging in physical activities, as it stimulates interlimb muscle activation and enhances the body's preparedness for dynamic physical and sports activities (see Figure 3). Therefore, isometric contraction of the upper limb could be advantageous in injury prevention and early rehabilitation stages.

Muscle synergies play a pivotal role in optimizing hamstring strength and function in ACL reconstruction patients (Saki et al., 2023; Zumstein et al., 2022). The PMKC framework emphasizes the significance of these synergies in improving neuromuscular coordination and movement efficiency. Isometric exercises that target the musculature of the upper limb can enhance hamstring strength and functional results by improving knee muscle coordination and

synergy (Deng et al., 2022; Kasmi et al., 2023; Miller et al., 2019). Research has shown that incorporating upper body exercises, core stabilization, and lower limb resistance training enhances hamstring strength, joint stability, and functional performance in the mid and return to sport phase of ACLR (Bregenhof et al., 2023; Chao et al., 2018; Saki et al., 2023). This implies that an early-phase ACLR rehabilitation program may include the ULIT to enhance hamstring activation.

In summary, current evidence regarding the influence of upper limb isometric training on hamstring strength in early-phase ACLR offers a theoretical framework for comprehending the potential mechanisms, physiological adaptations, and clinical implications of this rehabilitation intervention. There is limited research available to advise clinicians on the suitable intensity, volume, and frequency of the ULIT during the early phase of ACLR rehabilitation. Hence, the applicability of upper-limb isometric training to hamstring strength and functional results in the ACLR population is less established. Emerging findings support its role as a safe and effective supplementary therapy to enhance hamstring activation. Further research is necessary to elucidate the optimal role of upper limb isometric training in ACLR rehabilitation programs and develop evidence-based protocols that improve patient clinical outcomes.

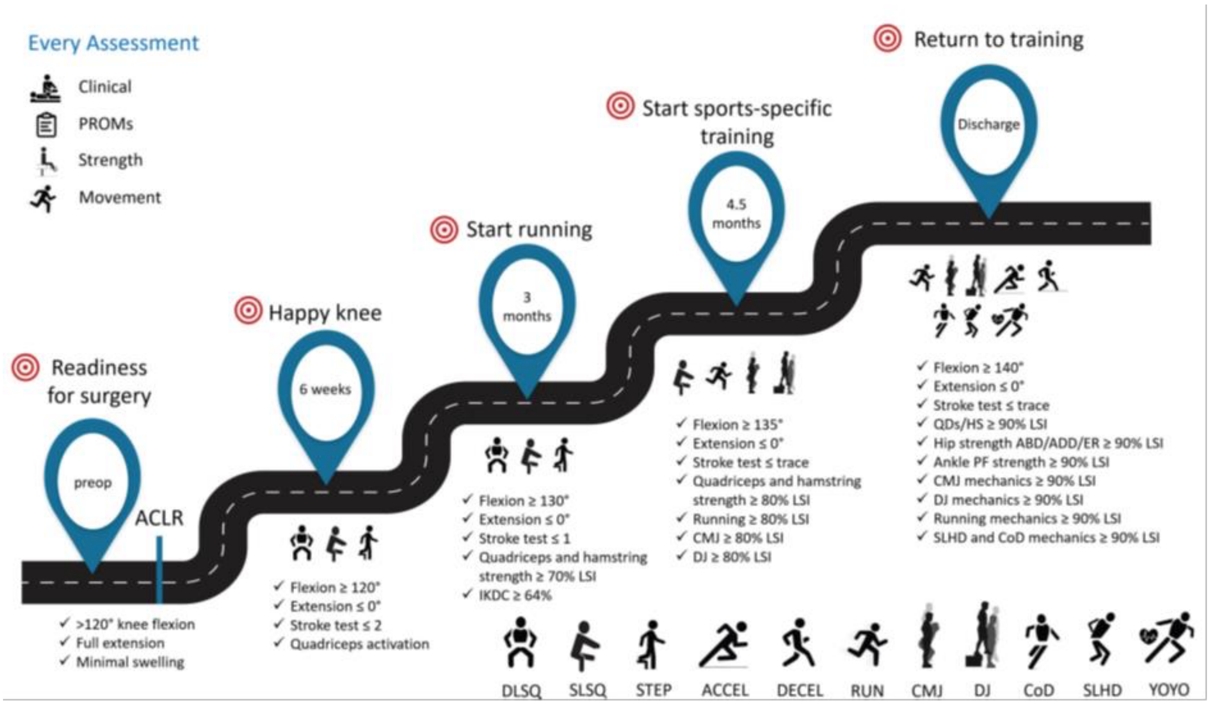

Figure 1a: ACLR Rehabilitation Phase and Goal (Aspetar, 2023).

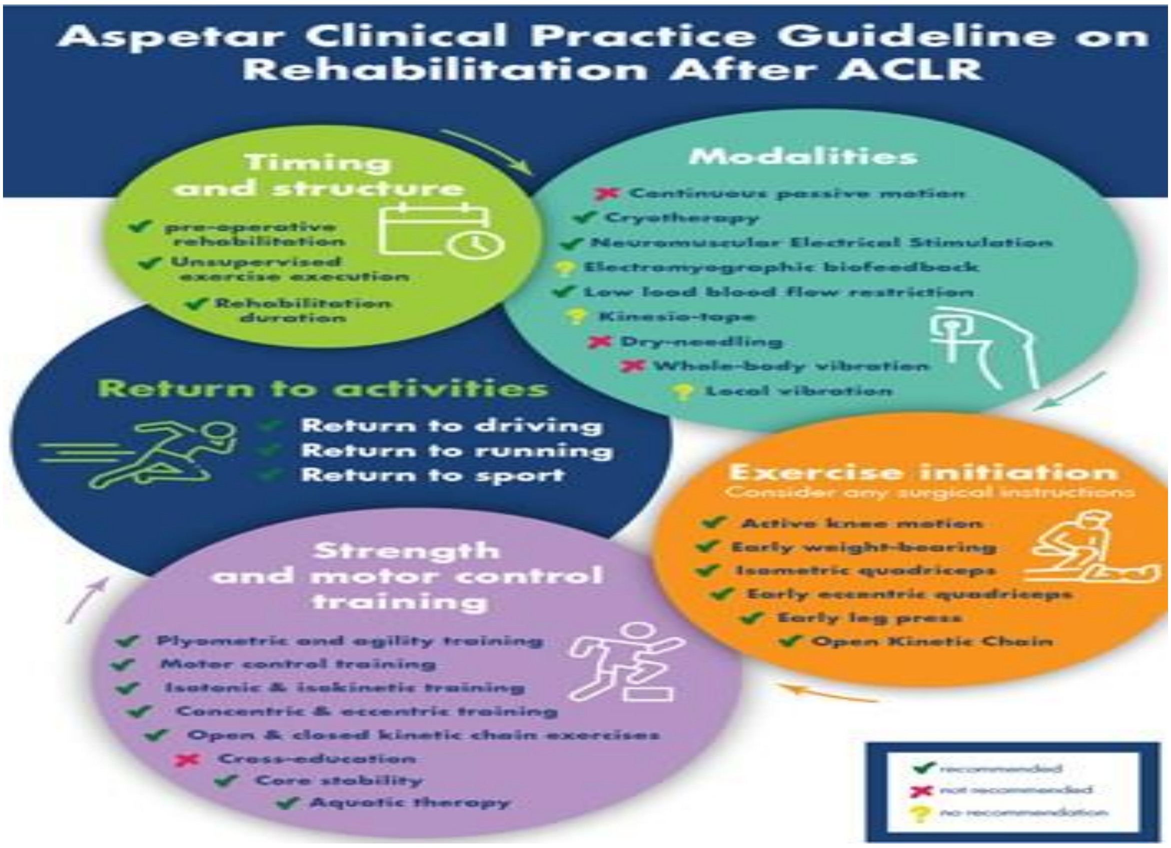

Figure 1b: ACLR Clinical Practice Guideline (Aspetar, 2023).

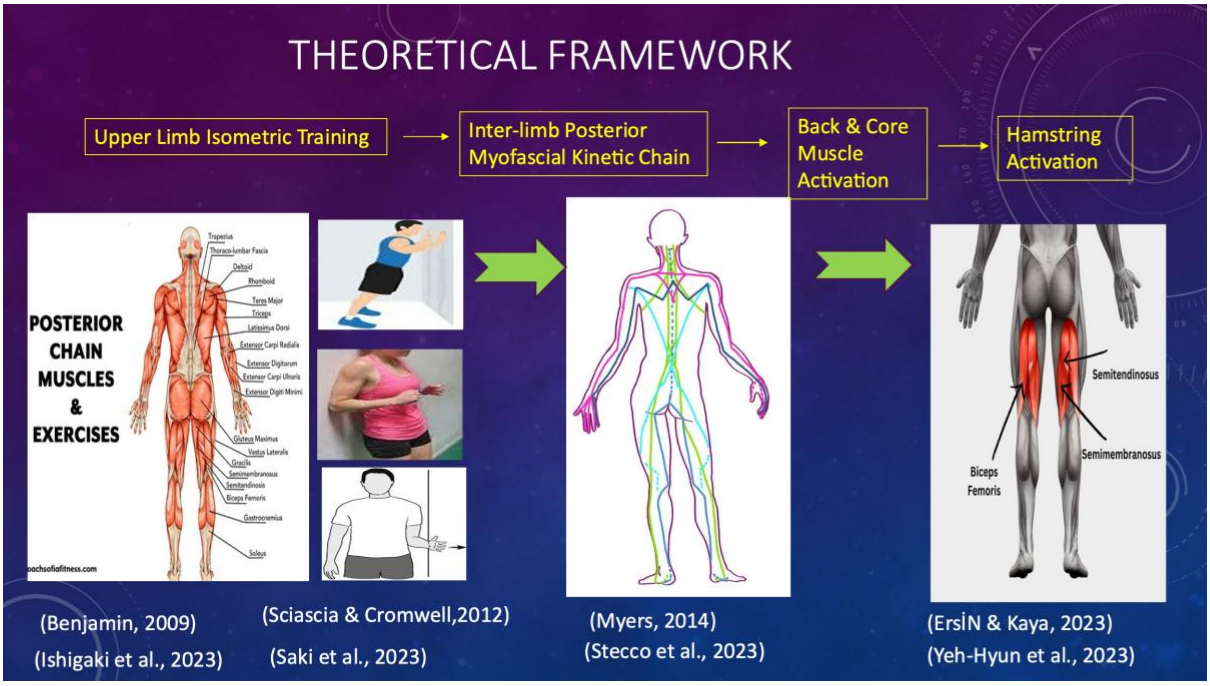

Figure 3: Proposed Mechanism and Exercises of ULIT.

Problem Statement

Considerable hamstring strength deficits following ACLR impact patients' physical function and increase re-injury risk. The high prevalence of hamstring strength imbalances in early-phase ACLR rehabilitation, particularly with hamstring autografts, hinders recovery. Early postoperative ACLR challenges, such as swelling, donor site pain, and joint stiffness, complicate direct hamstring activation, eccentric loading, and stretching. This study aims to investigate the effect of specific upper limb isometric training (ULIT) combined with standard care rehabilitation on hamstring strength and physical function in early-phase postoperative ACLR patients, potentially offering a novel and effective approach to ACLR rehabilitation.

Research Significant and Benefits

Applying exercise to the unaffected limb can offer an alternative, feasible and novel approach for acute stage and early rehabilitation of ACLR. Initiating indirect training in the non-injured limb allows muscle activation without causing unnecessary pain and complications to the injured part. This approach may accelerate return to achieve optimal strength and physical function enhancement among post-operative ACLR patients.

Research Question

Does the combination of upper limb isometric training (ULIT) and a standard care rehabilitation protocol enhance hamstring strength among patients in the early postoperative phase of ACLR?

Research Objective

The main objective of this study is to investigate the effects of upper limb isometric training (ULIT) combined with standard care rehabilitation versus standard care rehabilitation alone on hamstring strength and physical function in early phase postoperative ACLR patients.

The secondary objectives are:

1) to assess the efficacy of ULIT compared to standard care in improving physical function three months after surgery.

2) to explore ULIT's effectiveness in enhancing hamstring flexibility at three months following ACLR

3) to assess patient adherence to ULIT.

Specific Objectives

1. To investigate the difference in hamstring strength between the ULIT and the standard care group compared to the standard care alone group at 4, 8, and 12 weeks post-ACLR.

2. To measure the changes in physical function based on the IKDC score in the ULIT group compared to the standard care group at preoperative and 12 weeks post-ACLR.

3. To evaluate the effect of ULIT on hamstring flexibility, measured by the active knee extension test (AKET) test, compared to standard care alone at preoperative and 12 weeks post-ACLR.

4. To analyse the relationship between patient adherence to ULIT and improvements in hamstring strength and physical function over the 12-week rehabilitation period.

Research Hypothesis

We hypothesise that participants receiving the ULIT plus standard care will have better hamstring strength at 12 weeks post-operatively compared to participants receiving a standard care rehabilitation program alone.

Null Hypothesis

There is no significant difference between the combination of ULIT and standard care rehabilitation compared to the standard care rehabilitation alone on hamstring strength in early phase ACLR.

Alternative hypothesis

The combination of ULIT and standard care rehabilitation significantly improves hamstring strength compared to standard care rehabilitation alone in early phase ACLR

Methods

The methods are reported following CONSORT guidelines for non-pharmacological treatment studies and the Template for Intervention Description and Replication (TIDieR) reporting of interventions.

Study Design

This is a longitudinal, parallel-group, concealed allocation, randomised (1:1) controlled, single-blinded (assessor) study conducted at a tertiary hospital. If participants satisfy the eligibility criteria, provide informed consent, and have completed baseline measurement testing, they will be randomly assigned to the study. Participants will be informed that they will be randomly

assigned to one of two study groups: 1) the intervention group (ULIT plus standard care ACLR rehabilitation protocol) or 2) the control group (standard care rehabilitation protocol).

Study setting

The trial will be carried out at the Orthopaedic Clinic and Physiotherapy Unit of Hospital Cancelor Tuanku Muhriz (HCTM). The majority of recruited participants will be located in the Klang Valley, where they will receive surgical care from the same team. The main distinction among them will be the source of funding and reimbursement for their surgeries. The project will seek ethics approval from the Institutional Review Board (IRB) at the Research Ethics Committee UKM (RECUKM). The trial will be done in compliance with the Declaration of Helsinki (2000) and will be registered with the Australian and New Zealand clinical trials registry (http:www.anctzctr.org.au).

Eligibility

Potential participants who meet the criteria for the study will receive an invitation to participate no later than two weeks prior to their ACLR surgery. The potential participants will be identified based on the elective operation list of the Orthopaedic Clinic of HCTM. The patients who meet the specified criteria will be provided with both oral and written information regarding the trial's conditions and will be required to sign a standardised consent form. The principal investigator will verbally present information about the trial to eligible participants. The inclusion criteria will be verified based on the patient's written medical records physical examination by the surgeon and through direct communication with the patient. Patients who choose not to participate in the trial will still receive standard care rehabilitation.

Inclusion/Exclusion Criteria

The inclusion criteria are as follows: individuals who plan for ACLR using a ipsilateral hamstring tendon autograft, with or without a meniscal injury, capable of understanding English and giving informed consent, and are aged between 18 to 45 years. The following factors are considered as exclusions: Individuals with a revision ACL surgery, a multi-ligament injury (such as instability in the collateral, posterolateral, medial, or posterior cruciate ligaments), the use of allografts, an upper limb and contralateral lower limb injury after ACL surgery.

Recruitment Feasibility

Our objective is to enrol 32 individuals from the list of ACLR elective surgical patients at the HCTM using an ipsilateral Hamstring autograft. Hence, the anticipated duration for recruiting 32 individuals is 14 months, with an average of 3-4 ACLR cases per month.

Randomisation and Allocation

Following the baseline assessments, individuals will be assigned either to the intervention group or the control group by a simple random sampling. The randomisation process will be performed using a computer-generated sequence numbering scheme ranging from one (1) to thirty-two (32), with an equal allocation ratio of 1:1. The concealment is achieved using a set of sealed and numbered envelopes. Allocation takes place following baseline testing (T1), when a staff of the Orthopaedic Department, who is not participating in the study, opens the next study envelope. Subsequently, they notify the group assignment to the attending physiotherapist. The envelopes will be securely stored and sealed in a cabinet to ensure security and prevent any unauthorised access. Measures will be implemented to restrict the number of authorised individuals who have access or authority to open the research envelopes, to prevent any potential bias in the allocation process. The statistical analysis will specifically focus on allocation codes, ensuring that the data analyzers remain unaware of the study group allocation (see Figure 9).

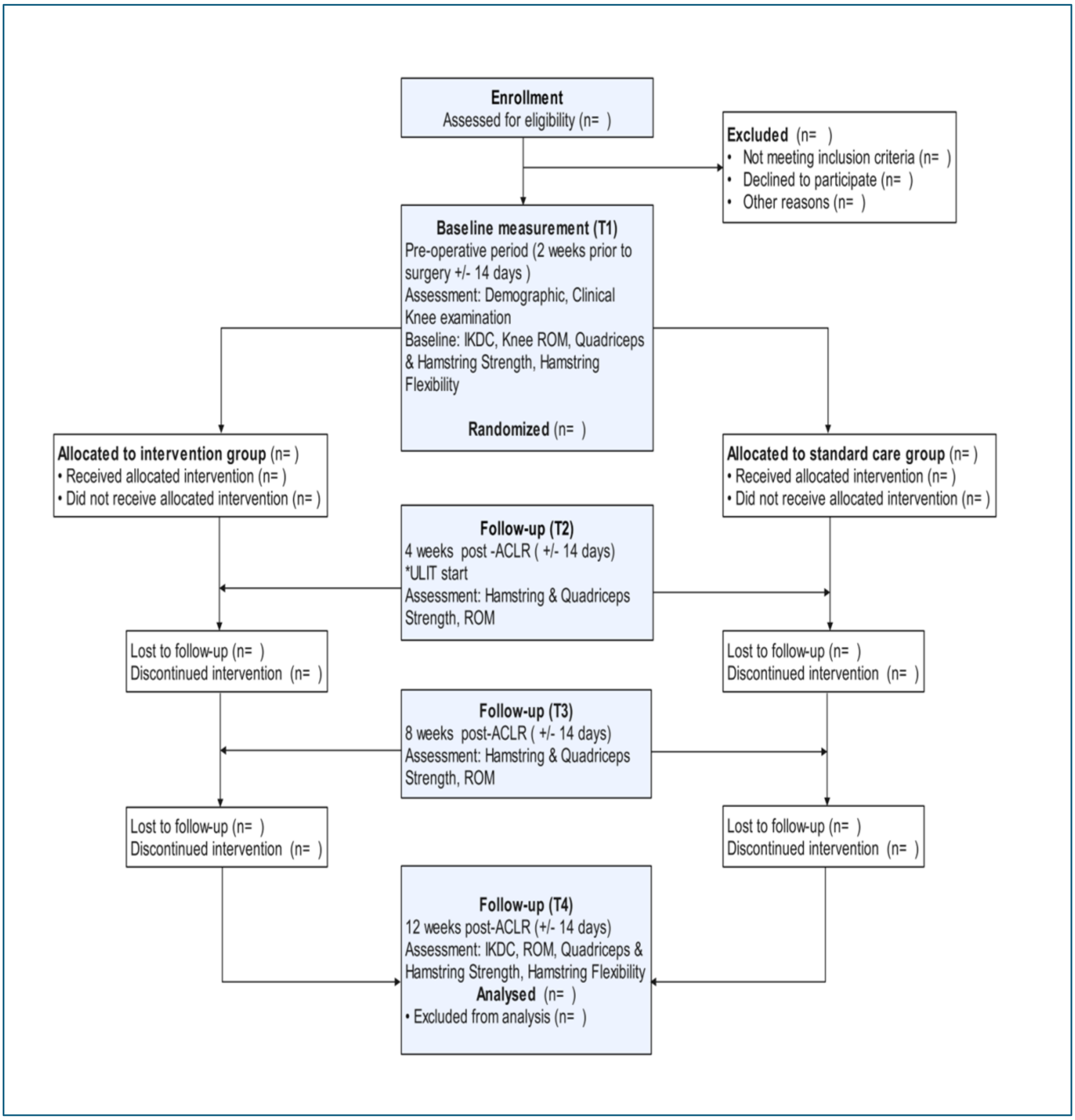

Figure 9: Study Three (RCT) Flow Chart

Control group (Standard care)

Patients will be directed to follow the standard care ACLR rehabilitation protocol from preoperative until 12 weeks after the ACLR (see Figure 10). Both study groups will receive standard physiotherapy care from the Department of Medical Rehabilitation Services (HCTM). For standard care rehabilitation sessions, there are five physiotherapists in charge with more than five years of clinical expertise in musculoskeletal rehabilitation. Current rehabilitation strategies consist of knee mobilisation exercises, cryotherapy, and progressive resistance exercises, including eccentric strengthening exercises, proprioceptive training, and dynamic stabilisation drills. The standard outpatient physiotherapy program takes about one hour per session, with a recommendation of 1-2 sessions per week until three months post-operative.

All participants are encouraged to diligently follow the designated daily home exercise program. The standard care involves a personalised therapeutic exercise programme that is specifically designed to meet the needs of each client's goal and the timeline of ACLR recovery. This physiotherapy outpatient rehabilitation program lasts approximately one hour per session, with 1-2 attended sessions per week recommended until three months post-operative. Furthermore, all participants are instructed to diligently adhere to the prescribed daily home exercise programme (see Appendix H).

Intervention Group (ULIT + Standard Care)

A single physiotherapist will administer the ULIT. The treating physiotherapist will present the ULIT to patients in verbal, demonstration, and written formats. In the fourth week following ACLR surgery, the treating physiotherapist will conduct the ULIT as a single, personalised session. The treating physiotherapist will instruct patients to follow the ULIT from 4 to 12 weeks post-operatively together with the standard care ACLR rehabilitation protocol. The ULIT comprised three concise isometric exercises: 1) wall push-up, 2) shoulder extension, and 3) shoulder external rotation (see Appendix I). The intervention group will be instructed to continue the ULIT plus standard care rehabilitation daily as a warm-up and home exercise programme for eight weeks.

Intervention Fidelity

A physiotherapist will conduct follow-up phone calls to evaluate exercise adherence for both groups. They will use standardized guidelines and handouts to encourage patients' exercise adherence, minimizing biases. A flexible and patient-centred approach is applied to address a patient's deviation from their rehabilitation protocol. This involves assessing and reevaluating the patient's progress, modifying the rehab plan for individual needs, rescheduling missed sessions, maintaining weekly communication (online exercise diary), documenting changes and analysing data under an intention-to-treat (ITT) protocol to reduce bias and preserve randomisation.

Blinding

The individuals responsible for evaluating the results and managing the data are unaware of the assigned therapy. Participants will be informed that they will be assigned randomly to either the standard care group or the intervention group. The patients are reminded not to reveal their group allocation to the physician or medical officer who performed the outcome assessment. The details of ULIT are not recorded in the medical record. A sports physician and medical officer, who is blinded to the group allocation and located away from the physiotherapy outpatient unit, will assess all outcome measures at the Orthopaedic Clinic. If any participant notifies the assessor about their post-operative training session, this information will be

recorded and reported. The rationale for this notification will be documented when the randomization is revealed. An intention-to-treat analysis will be conducted.

Due to the nature of the treatments, it will not be possible for physiotherapists to be blinded to the treatment allocation of patients. The primary investigator (PI) and data analyst (not from the same departments) will be unaware of the treatment allocation as data will be evaluated using coded identification numbers. The central study coordinator (a staff who is not involved in intervention and outcome assessment) will be responsible for coding and re-coding the identification numbers.

A half-day training workshop for blinded assessors and treating physiotherapists will be conducted before this study commences to enhance data quality and protocol adherence, reduce the bias risk of assessment and treatment, and increase the confidence and competence of the investigator.

To assure the overall standard and credibility of this clinical study, the process of disclosing the assigned treatment to participants will only be done in exceptional instances, for instance when there is a risk of harm, and when knowing the real treatment becomes essential for the participant's ongoing care. Before disclosing the group allocation, the PI will determine the necessity and amount of un-blinding. The allocation will remain confidential and will not be revealed to other study team members. If any code breaks occur, the PI will report it with the justifications.

Withdrawal from Trial

Following ACLR, each participant will have a scheduled follow-up visit at the HCTM Orthopaedic Outpatient Clinic. Every effort will be made to meet each participant's feasibility in attending the designated follow-up appointments (For example, flexible appointment times and dates). If they withdraw their consent, they will no longer be participants and will continue to receive the standard care rehabilitation and scheduled Orthopaedic Clinic follow-up. Information collected up to this point will be included.

Data Collection

The study will collect demographic data, as well as pre-, intra-, and postoperative data (see Table 3). Data will be gathered from the participants and their medical records for all the data collection forms (see Appendix A, C, and D). Baseline evaluations will be conducted throughout the pre-operative period, within a range of 14 days, to reduce any bias when selecting participants. The subsequent assessment in the 4th week (±14 days), 8 weeks (±14 days), and three months (±14 days) are scheduled at the Orthopaedic Clinic (van Melick et al., 2021). The blinded assessors will give all tests and questionnaires in person, ensuring participant consistency. These assessments will take place at four weeks, eight weeks and three months after the surgery. Phone consultations will be used for post-hospital discharge follow-up. Participants who cannot be reached by phone for a continuous period of 14 days starting from the assessment due date will be classified as lost to follow-up for outcome measurement.

	STUDY PERIOD					
		Eligibility	Baseline Measurement	Post-allocation		
			T1	*T2*	*T3*	*T4*
	TIME POINT (T)	*ACLR Surgery List*	*Pre-operative (14 days)*	*Weeks 4 (±14 days)*	*Weeks 8 (±14 days)*	Week 12 *(±14 days)*
ENROLMENT:	Eligibility screen	X				
	Enrolment	X				
	Informed consent	X				
	Demographics		X			
	Clinical Knee Examination		X			
	Random allocation		X			
INTERVENTIONS:	CONTROL: Standard care			←——————→		
	INTERVENTION: ULIT+ Standard care			←——————→		
ASSESSMENTS:	* Isometric Strength Test for Quadriceps and Hamstring		X	X	X	X
	Knee Range of Motion		X	X	X	X
	Active Knee Extension Test		X	X	X	X
	Numerical Pain Rating Scale		X	X	X	X
	International Knee Documentation Committee (IKDC) Form		X			X
	The Tampa Scale of Kinesiophobia		X			X
	The Sport Injury Rehabilitation Adherence Scale/ Exercise Diary			←——————→		
	Adverse Event			←——————→		

Table 3: Overview of trial procedures following the SPIRIT checklist for the schedule of enrolment, interventions, and assessments. * Primary outcome measure.

Outcomes Assessment

Primary outcome

A maximum voluntary isometric contraction (MVIC) test is employed to measure the maximum forces exerted by the quadriceps and hamstring muscles at pre-operative, 4-, 8-, and 12-weeks following ACLR (Harput et al., 2015). The subjects are positioned in high sitting with their hips flexed at 90 degrees. The knee is bent at an angle of 60 degrees, and the handheld dynamometer (*Wagner Force OneTM* - Model FDIX) force arm is fixed 5 centimetres above the lateral malleolus. During this position, the contraction of the hamstring muscles is unlikely to cause additional strain on the graft (Cristiani et al., 2021). The non-operated leg will be evaluated initially. The handheld dynamometer had ICC scores ranging from strong to excellent. The inter-reliability had a value of 0.9582 [95% C.I.: 0.9297–0.9752], whereas the

intra-reliability had a value of 0.9269 [95% C.I.: 0.8909–0.9533] (Grgic et al., 2020; Karagiannopoulos et al., 2022; Romero-Franco et al., 2019).

The participants are directed to generate deliberate isometric contractions of their quadriceps or hamstring muscles, by pushing against the resistance provided by the dynamometer's arm with a perceived maximal effort of 100% (Grgic et al., 2020). During the formal testing, the participants conducted three maximum voluntary isometric contractions (MVIC) of knee extension lasting five seconds each for the quadriceps (Padulo et al., 2020). Similarly, This is followed by knee flexion testing for the hamstrings. A minute rest time will be given between the quadriceps and hamstring testing (Mellemkjær et al., 2024).

The assessor will consistently give instruction and verbal encouragement (see Appendix I). The participants are instructed to exert maximum force by either pushing with their quadriceps or pulling with their hamstrings against the dynamometer. The average and best scores of the peak forces will be recorded (Grgic et al., 2020).

The average scores of quadriceps and hamstring muscular strength during rehabilitation are quantified using the Quadriceps Index (QI) and Hamstring Index (HI), which are calculated using the formula: (MVIC peak force of the affected limb / MVIC peak force of the unaffected limb) × 100 (van Melick et al., 2021).

Secondary/Explorative Outcomes:

All trial participants will complete the International Knee Documentation Committee Subjective Knee Form (IKDC-SKF) questionnaire (Appendix B) to evaluate their perception of daily knee function and associated symptoms (Jeon et al., 2022; Roula et al., 2023). The IKDC, a free online patient-reported outcome questionnaire that does not require licensing for non-commercial research, evaluates three crucial aspects of the patient's condition: symptoms, athletic activity, and knee function. The questionnaire is self-explanatory, meaning that it is simple for patients to understand and complete on their own. It has been created and confirmed to be effective for the population undergoing ACL reconstruction and comprehensively covers The International Classification of Functioning, Disability and Health (ICF) domains (Zebis et al., 2019). The Intra-Class Correlation (ICC) of the IKDC showed acceptable validity and responsiveness. The subjective form achieved excellent scores for construct validity, with 84% confirmation of preset hypotheses, and responsiveness, with 86% confirmation, in its test-retest reliability assessment (Jeon et al., 2022; Richardson et al., 2023).

The Active Knee Extension Test (AKET) will use an inclinometer to assess participants' hamstring flexibility. It relies on measuring the knee extension angle (KEA). We determine the hamstring flexibility by recording the KEA value plus 90°. The participant lies supine, with their hip in a 90° flexion position and a flat position for the contralateral leg. They are asked to extend their knee until they feel maximum tightness in the posterior thigh without pain, maintaining a relaxed ankle position to reduce the impact of the gastrocnemius muscle. In the interim, the tester will ensure that the tested hip is in a 90° flexion position by reading the inclinometer on the thigh. One or more of the following three criteria can determine the endpoint for AKET: (a) the examiner's perception of firm resistance; (b) the visible onset of pelvic rotation; and (c) the participant experiencing a strong but tolerable stretch, slightly before the onset of pain. In a previous study (Niewiadomy et al., 2021), researchers proposed

a clinically significant improvement of 10.2°. The AKET has demonstrated high reliability in evaluating hamstring flexibility in healthy people, with intraclass correlation coefficients (ICC) of 0.87 for the dominant knee and 0.81 for the non-dominant knee, while the intra-rater (test-retest) reliability ICC scores varied between 0.75 and 0.97 (Olivencia et al., 2020).

Adherence monitoring

A threshold for adherence is established for the experimental group, targeting participants to report a self-perceived rating of ≥80% (Cimino & Braun, 2023). Participants are advised to adhere to the instructions on home-based programs included in their respective flyers. The study used self-report scales and exercise diaries to measure adherence to the home exercise programme over 3 months. Participants are classified as adhered (≥ 3 times per week) or non-adhered (≤ 2 times per week) based on the average frequency of sessions performed weekly (Welling, 2024). Participants are instructed to complete the weekly exercise diary via online Google Form (see Appendix J). Physiotherapists will contact participants weekly via phone or WhatsApp to reinforce their exercise adherence. Self-report questionnaires and activity diaries have demonstrated efficacy, simplicity, and affordability as means of assessing exercise adherence in clinical and research contexts (Lahham et al., 2018; Nicolson et al., 2018).

The Sports Injury Rehabilitation Adherence Scale (SIRAS) is administered by a physician and physiotherapist to assess the level of adherence of all patients to the rehabilitation procedure in both study groups at the Orthopaedic Clinic and physiotherapy outpatient rehabilitation session (see Appendix E). The SIRAS is a reliable measure for evaluating clinic-based adherence in musculoskeletal rehabilitation with inter-rater and test-retest reliability coefficients ranging from 0.76 to 0.93 (Furlong & Hall, 2023).

ACL rehabilitation adherence and recovery can be significantly impacted by fear of movement, addressed by the Tampa Scale of Kinesophobia (TSK), a self-report checklist, utilising a 4-point likert scale (Ohji et al., 2022). By monitoring this fear, clinicians can improve compliance and modify rehabilitation programs (Kasmi et al., 2023). The TSK enables personalised treatment to overcome physical and psychological barriers. The TSK will be assessed preoperatively and at 3 months post-ACL reconstruction to monitor changes in fear of movement and reinjury (see Appendix F). The Tampa Scale of Kinesiophobia (TSK) is available online for free use at https://eprovide.mapi-trust.org/instruments/tampa-scale-for-kinesiophobia-11, requiring only proper citation and acknowledgement in relevant publications or research (Woby et al., 2005).

Adverse events

Adverse events will be observed through a non-leading questionnaire (Appendix G) throughout the entire phase of the intervention, as an element of the participant's training diary. Participants are welcome to contact the PI and the relevant physiotherapist(s) whenever necessary throughout the study period. Any adverse events or harm to participants during the intervention will be communicated to the principal investigator daily. We intend to disclose and publish the findings, regardless of their outcome.

Sample Size

Sample size calculations are performed using SPSS version 29 for the primary outcome: differences between two means of Hamstring MVIC peak force symmetry index (see Figure 11). With the population standard deviation for the main outcome measure amount of 0.71 as in the Harput et al. study, type I error of 0.05, type II error of 0.2, 80% power, the minimum sample size required is 14 patients per group (28 in total). Expecting an attrition rate of 10%, 16 participants are considered for each study group.

Table 1 Hamstring and Quadriceps Muscle Strength Outcomes at 4, 8, and 12 Weeks, Mean ± SD

Muscle	Limb	Time Point 4 wk	8 wk	12 wk	*P*
Hamstring peak torque (Nm/kg)	Involved	1.0 ± 0.5*	1.32 ± 0.9*	1.55 ± 0.8*	<.001
	Uninvolved	1.86 ± 0.34†	1.89 ± 0.46	2.05 ± 0.38†	.001
Quadriceps peak torque (Nm/kg)	Involved	1.47 ± 0.62*	2.18 ± 0.65*	2.61 ± 0.67*	.001
	Uninvolved	2.60 ± 0.63*	2.88 ± 0.73*	3.14 ± 0.64*	.001

*Quadriceps and hamstrings peak torques were significantly different between the 4th, 8th, and 12th weeks for the involved limb, and quadriceps peak torque was significantly different between the 4th, 8th, and 12th weeks for the uninvolved limb. †Significant difference for hamstrings peak torque between the 4th and 12th weeks for the uninvolved limb.

Figure 11: Sample size for the RCT based on the study by Harput (2015)

Data Management and Confidentiality

Only the supervisor, Principal Investigator (PI), co-researcher, and independent statistician have the right to access the data, and it will not be disclosed to any non-relevant party. The researcher supervisor and the PI will provide advisory support to the trial investigators in monitoring withdrawals, assessing ethical conduct, addressing missing data and reviewing major adverse events. A case report form (CRF) will record instances of unavailable data, ensuring uniformity and precision, and providing essential information for analysis and management. The data will be stored in a secure cabinet in the office of the PI and will be preserved for at least 5 years after the study is finished and published.

Statistical Analysis

The data analysis will be conducted using the SPSS Windows Version 29.0 software (SPSS, Chicago, IL, USA). The data will be summarised using descriptive statistics such as the mean and standard deviation, median and interquartile range, quantity and percentage, and frequency, depending on the distribution and type of data. This encompasses participant demographics as well as adherence to exercise. To evaluate the normality of the data, we will examine the skewness and kurtosis statistics (the ratio of the statistic to the standard error) by conducting the Shapiro-Wilk test. Boxplots are employed to detect outliers.

The main outcome measure, the mean changes in hamstring isometric peak force between 4 and 12 weeks, will be assessed using a paired t-test. The main hypothesis will be tested by comparing the changes in the mean difference between the intervention group and the standard

care group. The missing data will be analysed according to the intention-to-treat concept, considering the groups to which participants have been randomly allocated. Descriptive analysis, on the other hand, will describe missing data patterns in great detail to look for possible biases. If the data distribution exhibits non-normality or ceiling/floor effects, a non-parametric test (specifically, the Mann-Whitney test) will be conducted. Furthermore, this study will employ one-way repeated-measures ANOVA within-group measurements to investigate potential differences between the Quadriceps Index and Hamstring Index over an interval of time.

The secondary outcome data, which includes measurements of hamstring flexibility, range of motion, IKDC scores, and exercise adherence, will be summarised and evaluated in a manner consistent with the primary result. A repeated measures analysis of variance (ANOVA) will be used to examine the impact of rehabilitation and time on all the measured variables.

Statistical significance will be determined by a p-value of less than 0.05 (two-tailed) for all conducted tests with a 95% confidence interval.

Duration and Timeline

The recruitment process for all 32 participants is expected to be achieved from November 2024 to February 2025. The process of gathering data will be concluded and thoroughly examined by Mei 2026. The final manuscript will be prepared following the CONSORT extensions for a pragmatic trial employing a non-pharmacological intervention.

Budget

No budget is allocated for this study, as personnel costs, equipment, and data management resources are freely available within the study setting.

Funding and Conflict of Interest

This study does not receive any funding, and the authors disclose no conflicts of interest.

Gantt Chart

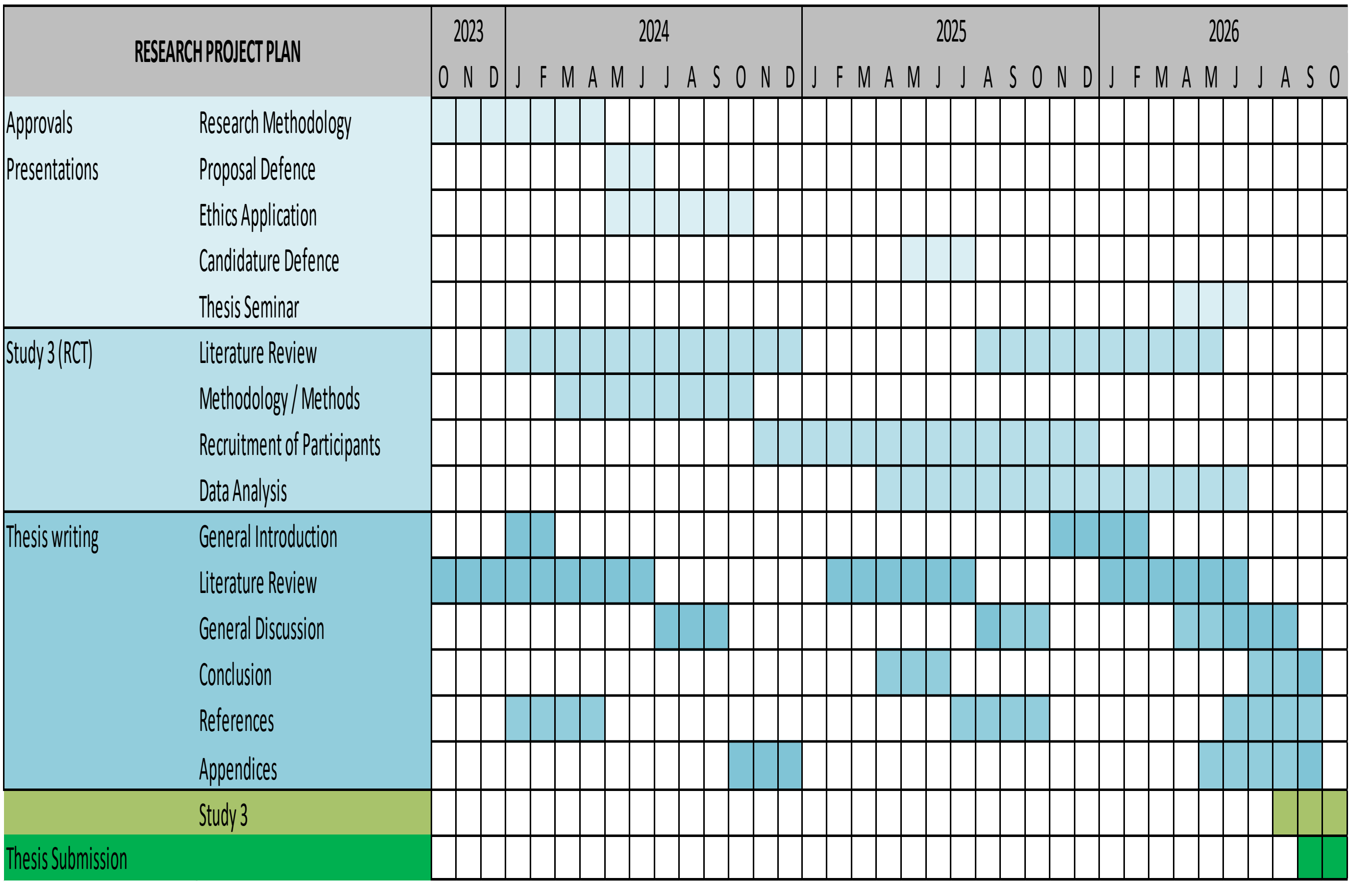

Conclusion and Clinical Relevance

This randomised clinical trial aims to assess the impact of specific upper limb isometric training on hamstring strength among individuals who have undergone ACLR. As a potential randomised controlled trial, this study aims to offer substantial evidence about the possible clinical and functional advantages of implementing an indirect exercise during the initial phase after ACLR rehabilitation, specifically utilising hamstring autografts. Currently, no clinical trials have been conducted to assess the impact of combining upper limb resistance training with a standardised rehabilitation program during the acute stage of ACLR. If proven efficacious, the intervention paradigm described in this study has the potential to enhance current treatment strategies and offer an alternative and novel approach in the early phase of ACLR rehabilitation.

References

Aspetar. (2023). *Aspetar ACL Rehabilitation Protocol*. Aspetar. https://www.aspetar.com/en/acl-rehabilitation-protocol

Bregenhof, B., Aagaard, P., Nissen, N., Creaby, M. W., Thorlund, J. B., Jensen, C., Torfing, T., & Holsgaard-Larsen, A. (2023). The Effect of Progressive Resistance Exercise on Knee Muscle Strength and Function in Participants with Persistent Hamstring Deficit Following ACL Reconstruction: A Randomized Controlled Trial. *J Orthop Sports Phys Ther*, *53*(1), 40-48. https://doi.org/10.2519/jospt.2022.11360

Chao, W. C., Shih, J. C., Chen, K. C., Wu, C. L., Wu, N. Y., & Lo, C. S. (2018). The Effect of Functional Movement Training After Anterior Cruciate Ligament Reconstruction: A Randomized Controlled Trial. *J Sport Rehabil*, *27*(6), 541-545. https://doi.org/10.1123/jsr.2017-0022

Cimino, J., & Braun, C. (2023). Design a Clinical Research Protocol: Influence of Real-World Setting. *Healthcare*, *11*(16).

Cristiani, R., Mikkelsen, C., Wange, P., Olsson, D., Stålman, A., & Engström, B. (2021). Autograft type affects muscle strength and hop performance after ACL reconstruction. A randomised controlled trial comparing patellar tendon and hamstring tendon autografts with standard or accelerated rehabilitation. *Knee Surg Sports Traumatol Arthrosc*, *29*(9), 3025-3036. https://doi.org/10.1007/s00167-020-06334-5

Deng, N., Soh, K. G., Zaremohzzabieh, Z., Abdullah, B., Salleh, K. M., & Huang, D. (2022). Effects of Combined Upper and Lower Limb Plyometric Training Interventions on Physical Fitness in Athletes: A Systematic Review with Meta-Analysis. *Int J Environ Res Public Health*, *20*(1). https://doi.org/10.3390/ijerph20010482

Fairus, F. Z., Ibrahim, S. A., Md Nadzalan, A., Md Yusoff, B. A. H., Mohamad, N., Hendri, E. N., Harithasan, D., Tengah, R. Y., & Mohd Nordin, N. A. (2022). Pattern of Anterior Cruciate Ligament Reconstruction (ACLR) among Athletes in Malaysia between 2015 and 2020. *Physical Education Theory and Methodology*, *22*(3s), S51-S58. https://doi.org/10.17309/tmfv.2022.3s.07

Forelli, F., Barbar, W., Kersante, G., Vandebrouck, A., Duffiet, P., Ratte, L., Hewett, T. E., & Rambaud, A. J. M. (2023). Evaluation of Muscle Strength and Graft Laxity With Early Open Kinetic Chain Exercise After ACL Reconstruction: A Cohort Study. *Orthopaedic Journal of Sports Medicine*, *11*(6), 23259671231177594. https://doi.org/10.1177/23259671231177594

Fukunaga, T., Johnson, C. D., Nicholas, S. J., & McHugh, M. P. (2019). Muscle hypotrophy, not inhibition, is responsible for quadriceps weakness during rehabilitation after anterior cruciate ligament reconstruction. *Knee Surg Sports Traumatol Arthrosc*, *27*(2), 573-579. https://doi.org/10.1007/s00167-018-5166-1

Furlong, B., & Hall, A. (2023). Clinimetrics: The Sport Injury Rehabilitation Adherence Scale. *Journal of Physiotherapy*, 1. https://doi.org/10.1016/j.jphys.2023.10.014

Grgic, J., Lazinica, B., Schoenfeld, B. J., & Pedisic, Z. (2020). Test–Retest Reliability of the One-Repetition Maximum (1RM) Strength Assessment: a Systematic Review. *Sports Medicine - Open*, *6*(1), 31. https://doi.org/10.1186/s40798-020-00260-z

Harput, G., Kılınç, H., Özer, H., Baltaci, G., & Mattacola, C. (2015). Quadriceps and Hamstring Strength Recovery During Early Period of Neuromuscular Rehabilitation Following ACL Hamstring Tendon Autograft Reconstruction. *Journal of Sport Rehabilitation*, *24*. https://doi.org/10.1123/jsr.2014-0224

Herbawi, F., Lozano-Lozano, M., Lopez-Garzon, M., Postigo-Martin, P., Ortiz-Comino, L., Martin-Alguacil, J. L., Arroyo-Morales, M., & Fernandez-Lao, C. (2022). A Systematic Review and Meta-Analysis of Strength Recovery Measured by Isokinetic Dynamometer Technology after Anterior Cruciate Ligament Reconstruction Using Quadriceps Tendon Autografts vs. Hamstring Tendon Autografts or Patellar Tendon Autografts. *Int J Environ Res Public Health*, *19*(11). https://doi.org/10.3390/ijerph19116764

Hiemstra, L. A., Webber, S., MacDonald, P. B., & Kriellaars, D. J. (2000). Knee strength deficits after hamstring tendon and patellar tendon anterior cruciate ligament reconstruction. *Med Sci Sports Exerc*, *32*(8), 1472-1479. https://doi.org/10.1097/00005768-200008000-00016

Högberg, J., Piussi, R., Wernbom, M., Della Villa, F., Simonsson, R., Samuelsson, K., Thomeé, R., & Hamrin Senorski, E. H. (2024). No Association Between Hamstrings-to-Quadriceps Strength Ratio and Second ACL Injuries After Accounting for Prognostic Factors: A Cohort Study of 574 Patients After ACL-Reconstruction. *Sports Medicine - Open*, *10*(1), 7. https://doi.org/10.1186/s40798-023-00670-9

Jeon, Y. S., Lee, J. W., Kim, S. H., Kim, S. G., Kim, Y. H., & Bae, J. H. (2022). Determining the Substantial Clinical Benefit Values for Patient-Reported Outcome Scores After Primary ACL Reconstruction. *Orthop J Sports Med*, *10*(5), 23259671221091795. https://doi.org/10.1177/23259671221091795

Karagiannopoulos, C., Griech, S., & Leggin, B. (2022). Reliability and Validity of the ActivForce Digital Dynamometer in Assessing Shoulder Muscle Force across Different User Experience Levels. *Int J Sports Phys Ther*, *17*(4), 669-676. https://doi.org/10.26603/001c.35577

Kasmi, S., Sariati, D., Hammami, R., Clark, C. C. T., Chtara, M., Hammami, A., Salah, F. Z. B., Saeidi, A., Ounis, O. B., Granacher, U., & Zouhal, H. (2023). The effects of different rehabilitation training modalities on isokinetic muscle function and male athletes' psychological status after anterior cruciate ligament reconstructions. *BMC Sports Sci Med Rehabil*, *15*(1), 43. https://doi.org/10.1186/s13102-023-00645-z

Ko, M. S., Yang, S. J., Ha, J. K., Choi, J. Y., & Kim, J. G. (2012). Correlation between Hamstring Flexor Power Restoration and Functional Performance Test: 2-Year Follow-Up after ACL Reconstruction Using Hamstring Autograft. *Knee Surg Relat Res*, *24*(2), 113-119. https://doi.org/10.5792/ksrr.2012.24.2.113

Kositsky, A., Barrett, R. S., du Moulin, W., Diamond, L. E., & Saxby, D. J. (2022). Semitendinosus muscle morphology in relation to surface electrode placement in anterior cruciate ligament reconstructed and contralateral legs. *Front Sports Act Living*, *4*, 959966. https://doi.org/10.3389/fspor.2022.959966

Kuenze, C., Weaver, A., Grindstaff, T. L., Ulman, S., Norte, G. E., Roman, D. P., Giampetruzzi, N., Lisee, C. M., Birchmeier, T., Triplett, A., Farmer, B., Hopper, H., Sherman, D. A., Ness, B. M., Collins, K., Walaszek, M., Baez, S. E., Harkey, M. S., Tulchin-Francis, K., . . . Hart, J. M. (2023). Age-, Sex-, and Graft-Specific Reference Values From 783 Adolescent Patients at 5 to 7 Months After ACL Reconstruction: IKDC, Pedi-IKDC, KOOS, ACL-RSI, Single-Leg Hop, and Thigh Strength. *J Orthop Sports Phys Ther*, *53*(4), 1-8. https://doi.org/10.2519/jospt.2023.11389

Labanca, L., Rocchi, J. E., Giannini, S., Faloni, E. R., Montanari, G., Mariani, P. P., & Macaluso, A. (2022). Early Superimposed NMES Training is Effective to Improve Strength and Function Following ACL Reconstruction with Hamstring Graft regardless of Tendon Regeneration. *J Sports Sci Med*, *21*(1), 91-103. https://doi.org/10.52082/jssm.2022.91

Lee, O. S., & Lee, Y. S. (2020). Changes in hamstring strength after anterior cruciate ligament reconstruction with hamstring autograft and posterior cruciate ligament reconstruction with tibialis allograft. *Knee Surg Relat Res*, *32*(1), 27. https://doi.org/10.1186/s43019-020-00047-2

Matar, H. E., Platt, S. R., Bloch, B. V., James, P. J., & Cameron, H. U. (2021). A Systematic Review of Randomized Controlled Trials in Anterior Cruciate Ligament Reconstruction: Standard Techniques Are Comparable (299 Trials With 25,816 Patients). *Arthrosc Sports Med Rehabil*, *3*(4), e1211-e1226. https://doi.org/10.1016/j.asmr.2021.03.017

McPherson, A., Schilaty, N., Anderson, S., Nagai, T., & Bates, N. (2023). Arthrogenic muscle inhibition after anterior cruciate ligament injury: Injured and uninjured limb recovery over time. *Frontiers in Sports and Active Living*, *5*. https://doi.org/10.3389/fspor.2023.1143376

Mellemkjær, F., Madeleine, P., Nørgaard, J., Jorgensen, M., & Kristiansen, M. (2024). Assessing Isometric Quadriceps and Hamstring Strength in Young Men and Women: Between-Session Reliability and Concurrent Validity. *Applied Sciences*, *14*, 958. https://doi.org/10.3390/app14030958

Miller, W., Kang, M., Jeon, S., & Ye, X. (2019). A Meta-analysis of Non-local Heterologous Muscle Fatigue. *Journal of Trainology*, *8*(1), 9-18. https://doi.org/10.17338/trainology.8.1_9

Myers, T. W. L. M. T. (2014). *Anatomy trains: myofascial meridians for manual and movement therapists* (3rd ed.). Elsevier.

Niewiadomy, P., Szuścik-Niewiadomy, K., Kochan, M., & Kuszewski, M. T. (2021). The Relationship between Active and Passive Flexibility of the Knee Flexors. *Muscle Ligaments and Tendons Journal*, *11*(02), 360. https://doi.org/10.32098/mltj.02.2021.21

Nuhmani, S., & Zouhal, H. (2022). Correlation between Core Stability and Upper-Extremity Performance in Male Collegiate Athletes. *Medicina*, *58*(8). https://doi.org/10.3390/medicina58080982

Ohji, S., Aizawa, J., Hirohata, K., Ohmi, T., Mitomo, S., Koga, H., & Yagishita, K. (2022). Changes in subjective knee function and psychological status from preoperation to 6 months post anterior cruciate ligament reconstruction. *Journal of Experimental Orthopaedics*, *9*(1), 114. https://doi.org/10.1186/s40634-022-00551-2

Olivencia, O., Godinez, G. M., Dages, J., Duda, C., Kaplan, K., Kolber, M. J., Kaplan, & Kolber. (2020). The Reliability And Minimal Detectable Change Of The Ely And Active Knee Extension Tests. *Int J Sports Phys Ther*, *15*(5), 776-782. https://doi.org/10.26603/ijspt20200776

Padulo, J., Trajković, N., Cular, D., Grgantov, Z., Madić, D. M., Di Vico, R., Traficante, A., Alin, L., Ardigò, L. P., & Russo, L. (2020). Validity and Reliability of Isometric-Bench for Knee Isometric Assessment. *Int J Environ Res Public Health*, *17*(12). https://doi.org/10.3390/ijerph17124326

Pietrosimone, B., Lepley, A., Kuenze, C., Harkey, M., Hart, J., Blackburn, T., & Norte, G. (2022). Arthrogenic Muscle Inhibition Following Anterior Cruciate Ligament Injury. *Journal of Sport Rehabilitation*, *31*, 1-13. https://doi.org/10.1123/jsr.2021-0128

Pinto, F. G., Thaunat, M., Daggett, M., Kajetanek, C., Marques, T., Guimares, T., Quelard, B., & Sonnery-Cottet, B. (2017). Hamstring Contracture After ACL Reconstruction Is Associated With an Increased Risk of Cyclops Syndrome. *Orthop J Sports Med*, *5*(1), 2325967116684121. https://doi.org/10.1177/2325967116684121

Richardson, R. D., Casanova, M. P., Reeves, A. J., Ryu, S., Cady, A. C., & Baker, R. T. (2023). Evaluating Psychometric Properties of the International Knee Documentation

Committee Subjective Knee Form in a Heterogeneous Sample of Post-Operative Patients. *Int J Sports Phys Ther*, *18*(4), 923-939. https://doi.org/10.26603/001c.83940
Rob, P. A. J., Nicky van, M., Jan, B. A. v. M., Max, R., & Lodewijk, W. v. R. (2018). ACL reconstruction with hamstring tendon autograft and accelerated brace-free rehabilitation: a systematic review of clinical outcomes. *BMJ Open Sport & Exercise Medicine*, *4*(1), e000301. https://doi.org/10.1136/bmjsem-2017-000301
Romero-Franco, N., Fernández-Domínguez, J. C., Montaño-Munuera, J. A., Romero-Franco, J., & Jiménez-Reyes, P. (2019). Validity and reliability of a low-cost dynamometer to assess maximal isometric strength of upper limb. *Journal of sports sciences*, *37*(15), 1787-1793. https://doi.org/10.1080/02640414.2019.1594570
Roula, K., Vasileios, K., Enda, K., Olivia, B., Dustin, M., Michail, P., Andreas, B., Julius, L., Jan, W., & Rodney, W. (2023). Aspetar clinical practice guideline on rehabilitation after anterior cruciate ligament reconstruction. *British Journal of Sports Medicine*, *57*(9), 500. https://doi.org/10.1136/bjsports-2022-106158
Saki, F., Shafiee, H., Tahayori, B., & Ramezani, F. (2023). The effects of core stabilization exercises on the neuromuscular function of athletes with ACL reconstruction. *Sci Rep*, *13*(1), 2202. https://doi.org/10.1038/s41598-023-29126-6
San Jose, A. T., Maniar, N., Timmins, R. G., Beerworth, K., Hampel, C., Tyson, N., Williams, M. D., & Opar, D. A. (2023). Explosive hamstrings strength asymmetry persists despite maximal hamstring strength recovery following anterior cruciate ligament reconstruction using hamstring tendon autografts. *Knee Surgery, Sports Traumatology, Arthroscopy*, *31*(1), 299-307. https://doi.org/10.1007/s00167-022-07096-y
Smyth, C., Broderick, P., Lynch, P., Clark, H., & Monaghan, K. (2023). To assess the effects of cross-education on strength and motor function in post stroke rehabilitation: a systematic literature review and meta-analysis. *Physiotherapy*, *119*, 80-88. https://doi.org/10.1016/j.physio.2023.02.001
Sonnery-Cottet, B., Hopper, G. P., Gousopoulos, L., Vieira, T. D., Thaunat, M., Fayard, J.-M., Freychet, B., Ouanezar, H., Cavaignac, E., & Saithna, A. (2022). Arthrogenic Muscle Inhibition Following Knee Injury or Surgery: Pathophysiology, Classification, and Treatment. *Video Journal of Sports Medicine*, *2*(3), 26350254221086295. https://doi.org/10.1177/26350254221086295
Stecco, A., Giordani, F., Fede, C., Pirri, C., De Caro, R., & Stecco, C. (2023). From Muscle to the Myofascial Unit: Current Evidence and Future Perspectives. *Int J Mol Sci*, *24*(5). https://doi.org/10.3390/ijms24054527
Svantesson, E., Hamrin Senorski, E., Webster, K. E., Karlsson, J., Diermeier, T., Rothrauff, B. B., Meredith, S. J., Rauer, T., Irrgang, J. J., Spindler, K. P., Ma, C. B., Musahl, V., The Panther Symposium Acl Injury Clinical Outcomes Consensus, G., Fu, F. H., Ayeni, O. R., Della Villa, F., Della Villa, S., Dye, S., Ferretti, M., . . . Hao Zheng, M. (2020). Clinical Outcomes After Anterior Cruciate Ligament Injury: Panther Symposium ACL Injury Clinical Outcomes Consensus Group. *Orthop J Sports Med*, *8*(7), 2325967120934751. https://doi.org/10.1177/2325967120934751
van Melick, N., van der Weegen, W., & Van der Horst, N. (2021). Quadriceps and Hamstrings Strength Reference Values for Athletes With and Without Anterior Cruciate Ligament Reconstruction Who Play Popular Pivoting Sports, Including Soccer, Basketball, and Handball: A Scoping Review. *Journal of Orthopaedic & Sports Physical Therapy*, *52*, 1-35. https://doi.org/10.2519/jospt.2022.10693
Welling, W. (2024). Return To Sports After an ACL Reconstruction in 2024 – a Glass Half Full? A narrative review. *Physical Therapy in Sport*, *67*. https://doi.org/10.1016/j.ptsp.2024.05.001

Woby, S. R., Roach, N. K., Urmston, M., & Watson, P. J. (2005). Psychometric properties of the TSK-11: a shortened version of the Tampa Scale for Kinesiophobia. *Pain*, *117*(1-2), 137-144. https://doi.org/10.1016/j.pain.2005.05.029

Zbrojkiewicz, D., Vertullo, C., & Grayson, J. E. (2018). Increasing rates of anterior cruciate ligament reconstruction in young Australians, 2000-2015. *Med J Aust*, *208*(8), 354-358. https://doi.org/10.5694/mja17.00974

Zebis, M. K., Warming, S., Pedersen, M. B., Kraft, M. H., Magnusson, S. P., Rathcke, M., Krogsgaard, M., Døssing, S., & Alkjær, T. (2019). Outcome Measures After ACL Injury in Pediatric Patients: A Scoping Review. *Orthop J Sports Med*, *7*(7), 2325967119861803. https://doi.org/10.1177/2325967119861803

Zumstein, F., Centner, C., & Ritzmann, R. (2022). How limb dominance influences limb symmetry in ACL patients: effects on functional performance. *BMC Sports Science, Medicine and Rehabilitation*, *14*(1), 206. https://doi.org/10.1186/s13102-022-00579-y
