# Supplementary material for: Effect of upper limb isometric training (ULIT) on hamstring strength in early postoperative anterior cruciate ligament reconstruction patients: Study protocol for a randomized controlled trial
Source: PLoS One. 2025 Aug 21;20(8):e0319724. doi: 10.1371/journal.pone.0319724 (PMC12370102; doi:10.1371/journal.pone.0319724)
Supplement: S2 Appendix — (PDF) [file pone.0319724.s002.pdf]

S2 Appendix. Participant Information Sheets

PARTICIPANT INFORMATION SHEET

Research Title:
EFFECT OF UPPER LIMB ISOMETRIC TRAINING ON HAMSTRING STRENGTH IN EARLY POSTOPERATIVE ANTERIOR CRUCIATE LIGAMENT RECONSTRUCTION PATIENTS: A RANDOMISED CONTROLLED TRIAL

Study Site:
Hospital Canselor Tuanku Muhriz UKM

Introduction:
You are invited to participate in a research study. Before participating in this study, it is important that you take time to read and understand the information in this Information Sheet.

Purpose of Study:
This study aims to investigate the effects of upper limb isometric training (ULIT) combined with the standard care of the anterior cruciate ligament reconstruction (ACLR) rehabilitation program on hamstring strength, flexibility, and physical function among ACLR patients in HCTM with a hamstring tendon graft. It aims to address the lack of consensus on the most effective training approach for optimizing hamstring strength in the early stages of ACLR, which can lead to fewer return-to-sport opportunities and physical function deficits.

What will the study involve?
We will randomly assign participants to the study if they meet the eligibility criteria, provide informed consent, and complete baseline measurement testing. Participants will be informed that they will be randomly assigned to one of two study groups:
a) The control group (standard care rehabilitation protocol) or
b) The intervention group (ULIT plus standard care rehabilitation protocol)

The primary goals of the early phase of the standard care ACLR rehabilitation protocol are to control pain, reduce swelling, restore knee joint range of motion, optimise muscle strength, regain daily activities such as walking without crutches, and minimise muscle atrophy. Current rehabilitation strategies include knee mobilizing exercises, cryotherapy, and progressive resistance exercises. Furthermore, standard rehabilitation protocols implement currently established interventions such as eccentric strengthening exercises, proprioceptive training, and dynamic stabilisation drills to improve activation and restore muscle function.

Patients in the intervention group will be instructed to follow the ULIT from week 4 to week 12 after the ACLR operation together with the standard care ACLR rehabilitation protocol. The ULIT program comprised three concise, submaximal-intensity exercises conducted in standing position as a home exercise program. This approach helps in enhancing neuromuscular pre-activation and improving overall muscle readiness for activity.

Evaluations of your knee strength, hamstring flexibility, functional testing and some written questionnaires relating to your knee health and exercise adherence will be conducted throughout the pre-operative period, 4th week, 8th week and 3 months at the Orthopaedic Clinic and Physiotherapy Unit of HCTM.

Benefits:

There is unlikely to be a direct benefit for you in participating in this study. Each participant will have a scheduled follow-up visit at the HCTM Orthopedic Clinic. Every effort will be made to meet each participant's feasibility to attend the designated follow-up appointments. The information gained from this study may help to guide rehabilitation protocols in the future, reduce the risk of re-injury, and provide more information about a safe return to sport post-surgery

Risks

There are no significant risks associated with the tests and treatment. However, there is an adverse event of muscle fatigue and soreness over the arm and forearm, although this risk is extremely low. A physiotherapist or physician, equipped with the necessary knowledge and skills to manage pain, swelling, and irritation in musculoskeletal health, can provide you with appropriate advice on how to manage any discomfort throughout the entire intervention phase. Participants are welcome to contact the principal investigator and the relevant physiotherapist(s) whenever necessary throughout the study period.

Do you have to take part?

Participation in this study is voluntary. If you agree to take part, then you will be asked to sign the "Informed Consent Form". You will be given a copy of the form and this Information Sheet.

Patients who choose not to participate in the trial will still receive standard care rehabilitation. Your data will not be used for publication and will be discarded. The researcher may also remove you from the study for a variety of reasons. In this event, you will not be penalised or lose your rights as a patient.

Data & Confidentiality:

The data from this study will be made into a report, which may be published. Access to the data is only available to the research team and RECUKM have access to the data. The data will be reported collectively with no reference to an individual. Hence, your identity will be kept confidential.

Payment and compensation:

You do not have to pay nor will you be paid to participate in this study.

Who can I ask about the study?
If you have any questions, you can direct them to the research team

Prof Madya Dato Dr Badrul Akmal Hisham Md Yusuf
Consultant
Jabatan Ortopedik
HCTM
E-mel: badortho@gmail.com
Contact Number: 0125196119

Efri Noor Muhamad Hendri
Senior Musculoskeletal Physiotherapist
Jabatan Perkhidmatan Pemulihan Perubatan
HCTM
E-mel: efri.noor.muhamad.hendri@ppukm.ukm.edu.my
Contact Number: 0132117407

Prof Madya Dr Mohamad Shariff Bin A Hamid
Sport Medicine Specialist
Unit Perubatan Sukan
Universiti Malaya
E-mel: ayip@um.edu.my
Contact Number: 03-79492994

Who should I contact if I am unhappy with how the study is being conducted?
You can contact the UKM Research Ethics Committee for clarifications.
(RECUKM reference number: JEP-2024-860)

Sekretariat Etika Penyelidikan Universiti Kebangsaan Malaysia,
Tingkat 1, Blok Klinikal,
Hospital Canselor Tuanku Muhriz, Pusat Perubatan UKM,
Jalan Yaacob Latif, Bandar Tun Razak,
56000 Cheras Kuala Lumpur.
E-mail: sepukm@ukm.edu.my
Tel: +603-9145 5046 / 9145 5048

HELAIAN MAKLUMAT PESERTA

Tajuk Penyelidikan:
KESAN LATIHAN ISOMETRIK ANGGOTA ATAS TERHADAP KEKUATAN OTOT HAMSTRING DALAM FASA AWAL PASCA PEMBEDAHAN REKONSTRUKTIF *ANTERIOR CRUCIATE LIGAMENT*: PERCUBAAN RAWAK TERKAWAL

Lokasi Kajian:
Hospital Canselor Tuanku Muhriz UKM

Pengenalan:
Anda dijemput untuk mengambil bahagian dalam kajian penyelidikan. Sebelum menyertai kajian ini, adalah penting untuk anda meluangkan masa untuk membaca dan memahami maklumat dalam Helaian Maklumat ini.

Tujuan Kajian:
Kajian ini bertujuan untuk menyiasat kesan latihan isometrik anggota atas (ULIT) yang digabungkan dengan penjagaan standard program pemulihan selepas pembedahan rekonstruktif *Anterior Cruciate Ligament* (ACLR) terhadap kekuatan hamstring, fleksibiliti dan fungsi fizikal di kalangan pesakit HCTM yang menggunakan auto-graf dari tendon hamstring. Ia bertujuan untuk menangani kekurangan konsensus tentang pendekatan latihan yang paling berkesan untuk mengoptimumkan kekuatan hamstring pada peringkat awal ACLR, yang boleh merencat peluang kembali ke sukan dan peningkatan risiko defisit fungsi fizikal.

Apakah latar belakang rawatan dalam kajian ini?
Jika anda memenuhi kriteria kelayakan, memberikan persetujuan bermaklum, dan melengkapkan ujian pengukuran asas. Peserta akan dimaklumkan bahawa mereka akan dimasukkan secara rawak kepada salah satu daripada dua kumpulan kajian:
a) Kumpulan kawalan (protokol penjagaan standard) atau
b) Kumpulan intervensi (ULIT dan protokol penjagaan standard).

Matlamat utama fasa awal protokol penjagaan standard pemulihan ACLR penjagaan adalah untuk mengawal kesakitan, mengurangkan bengkak, memulihkan julat pergerakan sendi lutut, mengoptimumkan kekuatan otot, mencapai kembali kefungsian aktiviti harian seperti berjalan tanpa tongkat, dan meminimumkan penyusutan saiz otot. Strategi pemulihan semasa termasuk senaman pergerakan sendi lutut, terapi ais, dan latihan rintangan progresif. Selain itu, protokol standard pemulihan ACLR juga melaksanakan intervensi seperti latihan kekuatan eksentrik, latihan proprioseptif, dan latihan penstabilan dinamik untuk meningkatkan pengaktifan dan memulihkan fungsi otot.

Pesakit dalam kumpulan intervensi akan diarahkan untuk melakukan senaman ULIT dari minggu ke-4 hingga minggu ke-12 selepas pembedahan ACLR beserta dengan protokol standard pemulihan ACLR. Program ULIT terdiri daripada tiga senaman ringkas, berintensiti sederhana dan akan menjadi sebahagian dari program senaman harian di rumah. Senaman ULIT ini dijangka dapat membantu dalam meningkatkan pra-aktivasi neuromuskular dan memperbaiki kesiapsiagaan otot secara keseluruhan untuk aktiviti fizikal.

Penilaian kekuatan lutut, julat pergerakan, fleksibiliti, ujian kefungsian dan beberapa soal selidik bertulis yang berkaitan dengan kesihatan sendi lutut anda dan kepatuhan pada senaman akan dijalankan dalam tempoh pra-pembedahan, minggu ke-4, minggu ke-8 dan 3 bulan di Klinik Ortopedik dan Unit Fisioterapi HCTM.

Manfaat:
Tidak ada manfaat secara langsung untuk anda dalam mengambil bahagian dalam kajian ini. Setiap peserta akan menjalani lawatan susulan yang dijadualkan di Klinik Ortopedik HCTM dan Unit Fisioterapi. Segala usaha akan dilakukan untuk membolehkan setiap peserta untuk menghadiri janji temu susulan yang ditetapkan. Maklumat yang diperoleh daripada kajian ini akan membantu menambahbaik protokol pemulihan pada masa hadapan, mengurangkan risiko kecederaan semula, dan memberi maklumat tambahan untuk para pesakit ACLR kembali ke aktiviti bersukan mahupun fizikal yang selamat.

Risiko:
Tiada risiko ketara yang berkaitan dengan ujian dan rawatan. Walau bagaimanapun, terdapat kesan sampingan seperti keletihan dan kesakitan pada otot lengan. Walaubagaimanapun risiko ini adalah rendah dan jarang berlaku. Ahli fisioterapi atau doktor, yang dilengkapi dengan pengetahuan dan kemahiran menguruskan kesakitan, bengkak dan kerengsaan dalam bidang muskuloskeletal, boleh memberi anda nasihat yang sesuai tentang cara menguruskan sebarang ketidakselesaan sepanjang keseluruhan fasa intervensi. Peserta dialu-alukan untuk menghubungi penyelidik dan ahli fisioterapi yang berkaitan apabila perlu sepanjang tempoh kajian.

Adakah anda perlu mengambil bahagian?
Penyertaan dalam kajian ini adalah sukarela. Jika anda bersetuju untuk mengambil bahagian, maka anda akan diminta untuk menandatangani "Borang Persetujuan Bermaklum". Salinan borang ini akan diberikan kepada anda.

Pesakit yang memilih untuk tidak mengambil bahagian dalam percubaan akan tetap menerima pemulihan penjagaan standard. Data anda tidak akan digunakan untuk penerbitan dan akan dihapuskan. Selain itu, Penyelidik juga berhak mengeluarkan anda daripada kajian atas pelbagai sebab. Dalam keadaan ini, anda tidak akan dihukum atau kehilangan hak anda sebagai pesakit.

Data & Kerahsiaan:
Data daripada kajian ini akan dijadikan laporan dan akan diterbitkan dalam jurnal akademik. Akses kepada data hanya tersedia untuk pasukan penyelidik dan RECUKM mempunyai akses kepada data tersebut. Data akan dilaporkan secara kolektif tanpa merujuk kepada individu. Oleh itu, identiti anda akan dirahsiakan.

Bayaran dan pampasan:
Anda tidak perlu membayar dan anda tidak akan dibayar untuk mengambil bahagian dalam kajian ini.

Siapa yang boleh saya hubungi untuk sebarang persoalan berkaitan kajian ini?
Jika anda mempunyai sebarang soalan, anda boleh mengemukan pertanyaan tersebut kepada pasukan penyelidik:

Prof Madya Dato Dr Badrul Akmal Hisham Md Yusuf
Perunding
Jabatan Ortopedik
HCTM
E-mel: badortho@gmail.com
Nombor telefon: 0125196119

Efri Noor Muhamad Hendri
Senior Muskuloskeletal Fisioterapis
Jabatan Perkhidmatan Pemulihan Perubatan
HCTM
E-mel: efri.noor.muhamad.hendri@ppukm.ukm.edu.my
Nombor telefon: 0132117407

Prof Madya Dr Mohamad Shariff Bin A Hamid
Pakar Perubatan Sukan
Unit Perubatan Sukan
Universiti Malaya
E-mel: ayip@um.edu.my
Nombor telefon: 0379492994

Siapakah yang perlu saya hubungi jika saya tidak berpuas hati dengan cara kajian dijalankan?
Anda boleh menghubungi Jawatankuasa Etika Penyelidikan UKM untuk tindakan lanjut:

Sekretariat Etika Penyelidikan Universiti Kebangsaan Malaysia,
Tingkat 1, Blok Klinikal,
Hospital Canselor Tuanku Muhriz, Pusat Perubatan UKM,
Jalan Yaacob Latif, Bandar Tun Razak,
56000 Cheras Kuala Lumpur.
E-mel: sepukm@ukm.edu.my
Tel: +603-9145 5046 / 9145 5048
