# Supplementary material for: Effect of upper limb isometric training (ULIT) on hamstring strength in early postoperative anterior cruciate ligament reconstruction patients: Study protocol for a randomized controlled trial
Source: PLoS One. 2025 Aug 21;20(8):e0319724. doi: 10.1371/journal.pone.0319724 (PMC12370102; doi:10.1371/journal.pone.0319724)
Supplement: S3 Appendix — (PDF) [file pone.0319724.s003.pdf]

S3 Appendix. Participant Consent Form

INFORMED CONSENT FORM

EFFECT OF UPPER LIMB ISOMETRIC TRAINING ON HAMSTRING STRENGTH IN EARLY POSTOPERATIVE ANTERIOR CRUCIATE LIGAMENT RECONSTRUCTION PATIENTS: A RANDOMISED CONTROLLED TRIAL

Please initial (/) the appropriate box	**YES**	**NO**
I have read the Information Sheet concerning this project and understand what it is about.	☐	☐
All my questions have been answered to my satisfaction. I understand that I am free to request further information at any stage.	☐	☐
My participation in the project is entirely voluntary.	☐	☐
I do not have to pay nor will be paid to participate in this study.	☐	☐
I know that I can quit the project at any time without any consequences, without having to give a reason, and without affecting my hospital follow-up.	☐	☐
I understand that personal information will be destroyed at the end of the project, but any raw data on which the project results are based will be kept in secure storage for research purposes only.	☐	☐
I am aware that the results of the project may be published in an academic journal, but my anonymity will be preserved.	☐	☐
**I agree to take part in this project**	☐	☐

Participant's Name: ______________________________ **Signature of Participant**: ______________________

Participant's NRIC: ______________________________ **Date:**______________________________________

Researcher Name: ______________________________ **Signature of researcher:** ______________________

Researcher's NRIC: ______________________________ **Date:** ______________________________________

Witness by: ________________________________ **Signature of Witness:** ________________________

Witness's NRIC: ________________________________ **Date:** ______________________________________

Designation: ---

This project is approved by the UKM Research Ethics Committee (RECUKM reference number: JEP-2024-860)

Appendix L: Informed Consent (Malay Version)

KESAN LATIHAN ISOMETRIK ANGGOTA ATAS TERHADAP KEKUATAN OTOT HAMSTRING DALAM FASA AWAL PASCA PEMBEDAHAN REKONSTRUKTIF ANTERIOR CRUCIATE LIGAMENT: PERCUBAAN RAWAK TERKAWAL

Sila tandakan (/) pada kotak yang sesuai	**YA**	**TIDAK**
Saya telah membaca Helaian Maklumat Peserta mengenai projek ini dan memahami maksudnya.	☐	☐
Semua soalan saya telah dijawab dengan memuaskan hati saya. Saya faham bahawa saya bebas untuk meminta maklumat lanjut pada mana-mana peringkat kajian.	☐	☐
Penyertaan saya dalam projek ini adalah sukarela sepenuhnya.	☐	☐
Saya tidak perlu membayar dan tidak akan dibayar untuk mengambil bahagian dalam kajian ini.	☐	☐
Saya tahu bahawa saya boleh berhenti daripada projek itu pada bila-bila masa tanpa sebarang akibat, tanpa perlu memberi alasan, dan tanpa menjejaskan temujanji susulan di HCTM.	☐	☐
Saya faham bahawa maklumat peribadi akan dimusnahkan pada akhir projek, tetapi sebarang rekod dan data akan disimpan dalam simpanan selamat untuk tujuan penyelidikan sahaja.	☐	☐
Saya sedar bahawa hasil projek itu mungkin diterbitkan dalam jurnal akademik tetapi kerahsiaan saya akan dikekalkan.	☐	☐
**Saya bersetuju untuk mengambil bahagian dalam penyelidikan ini**	☐	☐

Nama Peserta: ________________________________ **Tandatangan Peserta**: ____________________

Kad Pengenalan Peserta: ______________________ **Tarikh:** _________________________________

Nama Penyelidik:______________________________ **Tandatangan penyelidik:** _________________

Kad Pengenalan Penyelidik: ____________________ **Tarikh:** ________________________________

Disaksikan oleh: _______________________________ **Tandatangan Saksi:** ______________________

Kad Pengenalan Saksi: __________________________ **Tarikh:** _________________________________

Jawatan: _____________________________________

Projek ini diluluskan oleh Jawatankuasa Etika Penyelidikan UKM (rujukan no. **XXXXXXXX**)
