# Supplementary material for: Effect of upper limb isometric training (ULIT) on hamstring strength in early postoperative anterior cruciate ligament reconstruction patients: Study protocol for a randomized controlled trial
Source: PLoS One. 2025 Aug 21;20(8):e0319724. doi: 10.1371/journal.pone.0319724 (PMC12370102; doi:10.1371/journal.pone.0319724)
Supplement: S4 Appendix — (PDF) [file pone.0319724.s004.pdf]

S4 Appendix. International Knee Documentation Committee Subjective Knee Form (IKDC-SKF) Questionnaire.

IKDC

CASE REPORT FORM

INTRODUCTION

The entire IKDC form, which includes a demographic form, current health assessment form, subjective knee evaluation form, knee history form, surgical documentation form, and knee examination form, may be used as separate forms. The knee history form and surgical documentation form are provided for convenience. All researchers are required to complete the subjective knee evaluation and knee examination form. Instructions for scoring the subjective knee evaluation form and the knee examination form are provided on the back of the forms.

TABLE OF CONTENTS

1. **Demographic Form**
2. **Current Health Assessment Form**
3. **Subjective Knee Evaluation Form**
4. **Knee History Form**
5. **Surgical Documentation Form**
6. **Knee Examination Form**

IKDC DEMOGRAPHIC FORM

Patient Initial ___ ___ ___

Date of Birth ___ ___ / ___ ___ / ___ ___ ___ ___
Day Month Year

Patient MRN ___ ___ ___ ___ ___ ___ ___ ___ **Gender:** ❑ **Male** ❑ **Female**

Today's Date ___ ___ / ___ ___ / ___ ___ ___ ___
Day Month Year

The following is a list of common health problems. Please indicate "Yes" or "No" in the first column, and then skip to the next item. If you do have the problem, please indicate in the second column if you receive medications or some other type of treatment for the problem. In the last column, indicate if the problem limits any of your activities.

	Do you have the problem?		Do you receive treatment for it?		Does it limit your activities?	
	Yes	No	Yes	No	Yes	No
Heart disease	❑	❑	❑	❑	❑	❑
High blood pressure	❑	❑	❑	❑	❑	❑
Asthma or pulmonary disease	❑	❑	❑	❑	❑	❑
Diabetes	❑	❑	❑	❑	❑	❑
Ulcer or stomach disease	❑	❑	❑	❑	❑	❑
Bowel disease	❑	❑	❑	❑	❑	❑
Kidney disease	❑	❑	❑	❑	❑	❑
Liver disease	❑	❑	❑	❑	❑	❑
Anemia or other blood disease	❑	❑	❑	❑	❑	❑
Overweight	❑	❑	❑	❑	❑	❑
Cancer	❑	❑	❑	❑	❑	❑
Depression	❑	❑	❑	❑	❑	❑
Osteoarthritis, degenerative arthritis	❑	❑	❑	❑	❑	❑
Rheumatoid arthritis	❑	❑	❑	❑	❑	❑
Back pain	❑	❑	❑	❑	❑	❑
Lyme disease	❑	❑	❑	❑	❑	❑
Other medical problem	❑	❑	❑	❑	❑	❑
Alcoholism	❑	❑	❑	❑	❑	❑

Page 2 - IKDC DEMOGRAPHIC FORM

1. Do you smoke cigarettes?

   ❑Yes
   ❑No, I quit in the last six months.
   ❑No, I quit more than six months ago.
   ❑No, I have never smoked.

2. Your height________ centimeters

3. Your weight________ kilograms

4. Your race (indicate all that apply)

   ❑Malay ❑Chinese ❑Indian

   ❑Other _____________

5. How many schools have you completed?

   ❑Less than high school ❑Graduated from high school ❑Some college

   ❑Graduated from college ❑Postgraduate school or degree

6. Activity level

   ❑Highly competitive sports person

   ❑Well-trained and frequently sporting

   ❑Sporting sometimes

   ❑Non-sporting

IKDC CURRENT HEALTH ASSESSMENT FORM *

Patient Initial ___ ___ ___

Date of Birth ___ ___ / ___ ___ / ___ ___ ___ ___
Day Month Year

Today's Date ___ ___ / ___ ___ / ___ ___ ___ ___
Day Month Year

1. In general, would you say your health is: ❑Excellent ❑Very Good ❑Good ❑Fair ❑Poor

2. Compared to one year ago, how would you rate your health in general now?

   ❑Much better now than 1 year ago ❑Somewhat better now than 1 year ago ❑About the same as 1 year ago

   ❑Somewhat worse now than 1 year ago ❑Much worse now than 1 year ago

3. The following items are about activities you might do during a typical day. Does your health now limit you in these activities? If so, how much?

		Yes, Limited A Lot	Yes, Limited A Little	No, Not Limited At All
a.	Vigorous activities, such as running, lifting heavy objects, participating in strenuous sports	❑	❑	❑
b.	Moderate activities, such as moving a table, pushing a vacuum cleaner, bowling, or playing golf	❑	❑	❑
c.	Lifting or carrying groceries	❑	❑	❑
d.	Climbing several flights of stairs	❑	❑	❑
e.	Climbing one flight of stairs	❑	❑	❑
f.	Bending, kneeling or stooping	❑	❑	❑
g.	Walking more than a mile	❑	❑	❑
h.	Walking several blocks	❑	❑	❑
i.	Walking one block	❑	❑	❑
j.	Bathing or dressing yourself	❑	❑	❑

4. During the <u>past 4 weeks</u>, have you had any of the following problems with your work or other regular daily activities as a result of your physical health?

		YES	NO
a.	Cut down on the amount of time you spent on work or other activities	❑	❑
b.	Accomplished less than you would like	❑	❑
c.	Were limited in the kind of work or other activities	❑	❑
d.	Had difficulty performing the work or other activities (for example, it took extra effort)	❑	❑

5. During the <u>past 4 weeks</u>, have you had any of the following problems with your work or other regular daily activities as a result of any emotional problems (such as feeling depressed or anxious)?

		YES	NO
a.	Cut down on the amount of time you spent on work or other activities	❑	❑
b.	Accomplished less than you would like	❑	❑
c.	Didn't do work or other activities as carefully as usual	❑	❑

6. During the <u>past 4 weeks</u>, to what extent has your physical health or emotional problems interfered with your normal social activities with family, friends, neighbors, or groups?

❑Not At All ❑Slightly ❑Moderately ❑Quite a Bit ❑Extremely

7. How much bodily pain have you had during the <u>past 4 weeks</u>?

❑None ❑Very Mild ❑Mild ❑Moderate ❑Severe ❑Very Severe

8. During the <u>past 4 weeks</u>, how much did pain interfere with your normal work (including both work outside the home and housework)?

❑Not at All ❑A Little Bit ❑Moderately ❑Quite a Bit ❑Extremely

9. These questions are about how you feel and how things have been with you during the past 4 weeks. For each question, please give the one answer that comes closest to the way you have been feeling. How much of the time during the <u>past 4 weeks</u>…

	All of the time	Most of the time	A good bit of the time	Some of the time	A little of the time	None of the time
a. Did you feel full of pep?	❑	❑	❑	❑	❑	❑
b. Have you been very nervous?	❑	❑	❑	❑	❑	❑
c. Have you felt calm and peaceful?	❑	❑	❑	❑	❑	❑
d. Did you have a lot of energy?	❑	❑	❑	❑	❑	❑
e. Have you felt down-hearted and blue?	❑	❑	❑	❑	❑	❑
f. Did you feel worn out?	❑	❑	❑	❑	❑	❑
g. Have you been a happy person	❑	❑	❑	❑	❑	❑
h. Did you feel tired?	❑	❑	❑	❑	❑	❑

10. During the <u>past 4 weeks</u>, how much of the time has your physical health or emotional problems interfered with your social activities (like visiting with friends, relatives, etc.)?

❑All of the time ❑Most of the time ❑Some of the time ❑A little of the time ❑None of the time

11. How TRUE or FALSE is each of the following statements for you?

	Definitely True	Mostly True	Don't Know	Mostly False	Definitely False
a. I seem to get sick a little easier than other people	❑	❑	❑	❑	❑
b. I am as healthy as anybody I know	❑	❑	❑	❑	❑
c. I expect my health to get worse	❑	❑	❑	❑	❑
d. My health is excellent	❑	❑	❑	❑	❑

*This form includes questions from the SF-36™ Health Survey.

IKDC SUBJECTIVE KNEE EVALUATION FORM

Patient Initial ___ ___ ___

Today's Date ___ ___ / ___ ___ / ___ ___ ___ ___ Date of Injury ___ ___ / ___ ___ / ___ ___ ___ ___
Day Month Year Day Month Year

SYMPTOMS*:

*Grade symptoms at the highest activity level at which you think you could function without significant symptoms, even if you are not actually performing activities at this level.

1. What is the highest level of activity that you can perform without significant knee pain?

4❑Very strenuous activities like jumping or pivoting as in basketball or soccer
3❑Strenuous activities like heavy physical work, skiing or tennis
2❑Moderate activities like moderate physical work, running or jogging
1❑Light activities like walking, housework or yard work
0❑Unable to perform any of the above activities due to knee pain

2. During the past 4 weeks, or since your injury, how often have you had pain?

	0	1	2	3	4	5	6	7	8	9	10	
Never	❑	❑	❑	❑	❑	❑	❑	❑	❑	❑	❑	Constant

3. If you have pain, how severe is it?

	0	1	2	3	4	5	6	7	8	9	10	
No pain	❑	❑	❑	❑	❑	❑	❑	❑	❑	❑	❑	Worst pain imaginable

4. During the past 4 weeks, or since your injury, how stiff or swollen was your knee?

4❑Not at all
3❑Mildly
2❑Moderately
1❑Very
0❑Extremely

5. What is the highest level of activity you can perform without significant swelling in your knee?

4❑Very strenuous activities like jumping or pivoting as in basketball or soccer
3❑Strenuous activities like heavy physical work, skiing or tennis
2❑Moderate activities like moderate physical work, running or jogging
1❑Light activities like walking, housework, or yard work
0❑Unable to perform any of the above activities due to knee swelling

6. During the past 4 weeks, or since your injury, did your knee lock or catch?

0❑Yes 1❑No

7. What is the highest level of activity you can perform without significant giving way in your knee?

4❑Very strenuous activities like jumping or pivoting as in basketball or soccer
3❑Strenuous activities like heavy physical work, skiing or tennis
2❑Moderate activities like moderate physical work, running or jogging
1❑Light activities like walking, housework or yard work
0❑Unable to perform any of the above activities due to giving way of the knee

SPORTS ACTIVITIES:

8. What is the highest level of activity you can participate in on a regular basis?

   4❑Very strenuous activities like jumping or pivoting as in basketball or soccer
   3❑Strenuous activities like heavy physical work, skiing or tennis
   2❑Moderate activities like moderate physical work, running or jogging
   1❑Light activities like walking, housework or yard work
   0❑Unable to perform any of the above activities due to knee

9. How does your knee affect your ability to:

		Not difficult at all	Minimally difficult	Moderately Difficult	Extremely difficult	Unable to do
a.	Go up stairs	4❑	3❑	2❑	1❑	0❑
b.	Go downstairs	4❑	3❑	2❑	1❑	0❑
c.	Kneel on the front of your knee	4❑	3❑	2❑	1❑	0❑
d.	Squat	4❑	3❑	2❑	1❑	0❑
e.	Sit with your knee bent	4❑	3❑	2❑	1❑	0❑
f.	Rise from a chair	4❑	3❑	2❑	1❑	0❑
g.	Run straight ahead	4❑	3❑	2❑	1❑	0❑
h.	Jump and land on your involved leg	4❑	3❑	2❑	1❑	0❑
i.	Stop and start quickly	4❑	3❑	2❑	1❑	0❑

FUNCTION:

10. How would you rate the function of your knee on a scale of 0 to 10 with 10 being normal, excellent function and 0 being the inability to perform any of your usual daily activities which may include sports?

FUNCTION PRIOR TO YOUR KNEE INJURY:

	0	1	2	3	4	5	6	7	8	9	10	
Couldn't perform daily activities	❑	❑	❑	❑	❑	❑	❑	❑	❑	❑	❑	No limitation in daily activities

CURRENT FUNCTION OF YOUR KNEE:

	0	1	2	3	4	5	6	7	8	9	10	
Can't perform daily activities	❑	❑	❑	❑	❑	❑	❑	❑	❑	❑	❑	No limitation in daily activities

Scoring Instructions for the 2000 IKDC Subjective Knee Evaluation Form

Several methods of scoring the IKDC Subjective Knee Evaluation Form were investigated. The results indicated that summing the scores for each item performed as well as more sophisticated scoring methods.

The responses to each item are scored using an ordinal method such that a score of 0 is given to responses that represent the lowest level of function or highest level of symptoms. For example, item 1, which is related to the highest level of activity without significant pain is scored by assigning a score of 0 to the response "Unable to perform any of the above activities due to knee pain" and a score of 4 to the response "Very strenuous activities like jumping or pivoting as in basketball or soccer". **For item 2, which is related to the frequency of pain over the past 4 weeks, the responses are reverse-scored such that "Constant" is assigned a score of 0 and "Never" is assigned a score of 10. Similarly, for item 3, the responses are reversed-scored such that "Worst pain imaginable" is assigned a score of 0 and "No pain" is assigned a score of 10.** Note: previous versions of the form had a minimum item score of 1 (for example, ranging from 1 to 11). In the most recent version, all items now have a minimum score of 0 (for example, 0 to 10). To score these prior versions, you would need to transform each item to the scaling for the current version.

The IKDC Subjective Knee Evaluation Form is scored by summing the scores for the individual items and then transforming the score to a scale that ranges from 0 to 100. **Note**: The response to item 10a "Function Prior to Knee Injury" is not included in the overall score. To score the current form of the IKDC, simply add the score for each item (the small number by each item checked) and divide by the maximum possible score which is 87:

$$\text{IKDC Score} = \left[\frac{\text{Sum of Items}}{\text{Maximum Possible Score}}\right] \text{x}\, 100$$

Thus, for the current version, if the sum of scores for the 18 items is 45 and the patient responded to all the items, the IKDC Score would be calculated as follows:

$$\text{IKDC Score} = \left[\frac{45}{87}\right] \text{x}\, 100$$

$$\text{IKDC Score} = 51.7$$

The transformed score is interpreted as a measure of function such that higher scores represent higher levels of function and lower levels of symptoms. A score of 100 is interpreted to mean no limitation with activities of daily living or sports activities and the absence of symptoms.

The IKDC Subjective Knee Form score can be calculated when there are responses to at least 90% of the items (i.e. when responses have been provided for at least 16 items). In the original scoring instructions for the IKDC Subjective Knee Form, missing values are replaced by the average score of the items that have been answered. However, this method could slightly over- or under-estimate the score depending on the maximum value of the missing item(s) (2, 5 or 11 points). Therefore, in the revised scoring procedure for the current version of a form with up to two missing values, the IKDC Subjective Knee Form Score is calculated as (sum of the completed items) / (maximum possible sum of the completed items) * 100. This method of scoring the IKDC Subjective Knee Form is more accurate than the original scoring method.

A scoring spreadsheet is also available at: www.sportsmed.org/research/index.asp This spreadsheet uses the current form scores and the revised scoring method for calculating scores with missing values.

IKDC KNEE HISTORY FORM

Patient Initial ___ ___ ___

Birthdate ___ ___ / ___ ___ / ___ ___ ___ ___
Day Month Year

Date of Injury___ ___ / ___ ___ / ___ ___ ___ __
Day Month Year

Date of Initial Exam___ ___ / ___ ___ / ___ ___ ___ ___
Day Month Year

Involved Knee: ❑Right ❑Left

Contralateral: ❑Normal ❑Nearly Normal ❑Abnormal ❑Severely abnormal

Onset of Symptoms: ___ ___ / ___ ___ / ___ ___ ___ ___
Day Month Year

Chief Complaint:____

Activity at Injury: ❑ADL ❑Sports ❑Traffic ❑Work

Mechanism of Injury:

❑Non-traumatic gradual onset ❑Traumatic non-contact onset
❑Non-traumatic sudden onset ❑Traumatic contact onset

Previous Surgery:

Type of Surgery: (check all that apply)

Meniscal Surgery

❑Medial meniscectomy ❑Lateral meniscectomy
❑Medial meniscal repair ❑Lateral meniscal repair
❑Medial meniscal transplant ❑Lateral meniscal transplant

Ligament Surgery

❑ACL Repair ❑Intraarticular ACL reconstruction ❑Extraarticular ACL reconstruction
❑PCL Repair ❑Intraarticular PCL reconstruction ❑Posterolateral corner reconstruction
❑Medial collateral ligament repair/reconstruction
❑Lateral collateral ligament repair/reconstruction

Type of Graft

Patella tendon graft ❑Ipsilateral ❑Contralateral
❑Single hamstring graft
❑2 Bundle hamstring graft
❑4 Bundle hamstring graft
❑Quadriceps tendon graft
❑Allograft
❑Other

Extensor Mechanism Surgery

❑Patella tendon repair ❑Quadriceps tendon repair

Patellofemoral Surgery

❑Extensor Mechanism Realignment

Soft Tissue Realignment

❑Medial imbrication ❑Lateral release

Bone Realignment

Movement of the tibial tubercle
❑Proximal ❑Distal ❑Medial ❑Lateral ❑Anterior

❑Trochleoplasty

❑Patellectomy

Osteoarthritis Surgery

❑Osteotomy

❑Articular Surface Surgery ❑Shaving ❑Abrasion ❑Drilling ❑Microfracture
❑Cell therapy ❑Osteochondral autograft transfer/mosaic-plasty ❑Other

Total number of previous surgeries______________

Imaging Studies:

❑Structural ❑MRI ❑CT ❑Arthrogram

❑Metabolic (Bone Scan)

Findings:

Ligament____

Meniscus ____

Articular Cartilage____

Bone ____

IKDC SURGICAL DOCUMENTATION FORM

Patient's Initial: ____ ____ ____

Date of Procedure: ___ ___ / ___ ___ / ___ ___ ___ ___
Day Month Year

Postoperative Diagnosis:

1.____

2.____

3.____

Status After Procedure:

ARTICULAR CARTILAGE STATUS:

Document the size and location of articular cartilage defects on these figures according to the ICRS mapping system[c].

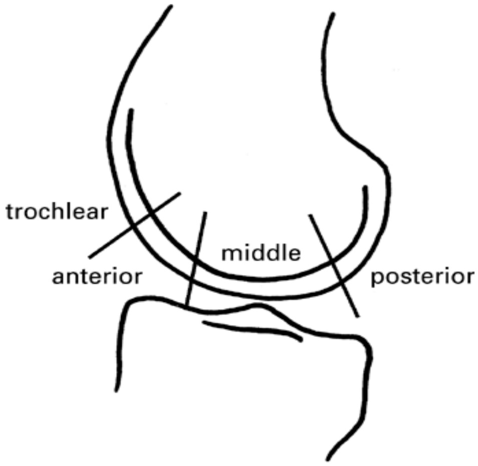

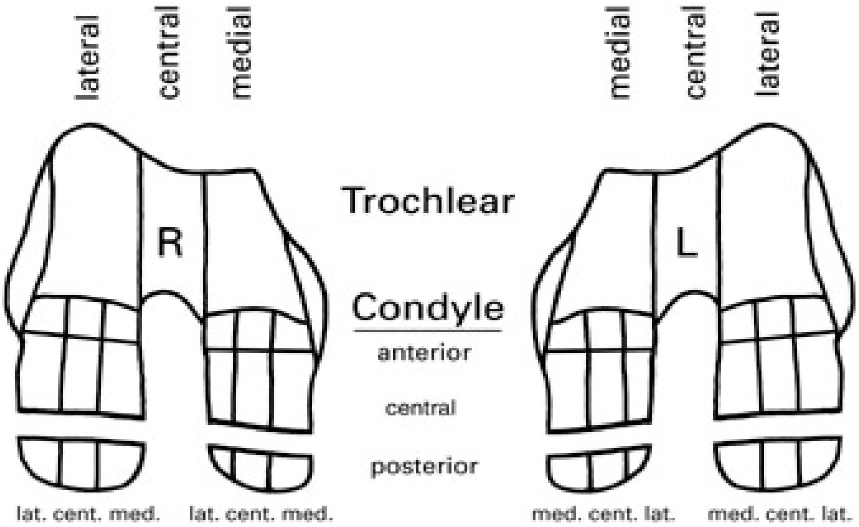

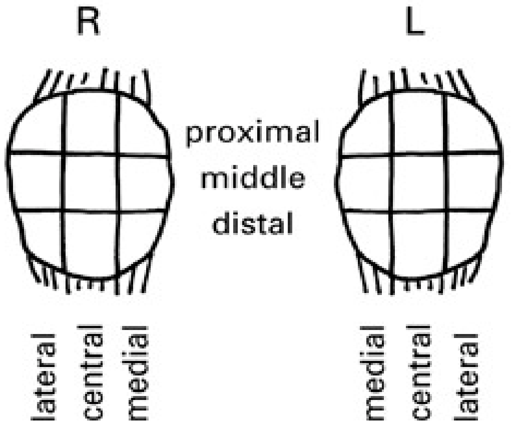

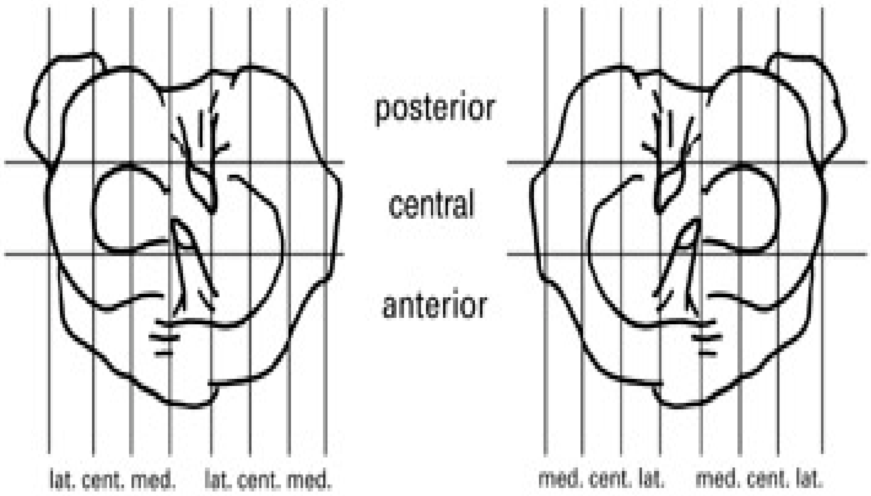

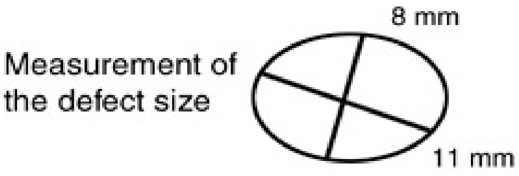

Page 2 – IKDC SURGICAL DOCUMENTATION FORM

Record size, location and grade of articular cartilage lesions.

Femur

First lesion   Second Lesion

Side	Right	Left		
Condyle	Medial	Lateral		
Sagittal plane	Trochlear	Anterior	Middle	Posterior
Frontal plane	Lateral	Central	Medial	

Cartilage lesion (Grade) (*)
Defect size pre-debridement mm
Defect size post-debridement mm

Tibia

Side	Right	Left	
Plateau	Medial	Lateral	
Sagittal Plane	Anterior	Middle	Posterior
Frontal Plane	Lateral	Central	Medial

Cartilage lesion (Grade) (*)
Defect size pre-debridement mm
Defect size post-debridement mm

Patella

Side	Right	Left	
Sagittal plane	Distal	Middle	Proximal
Frontal plane	Lateral	Central	Medial

Cartilage lesion (Grade) (*)
Defect size pre-debridement mm
Defect size post-debridement mm

Diagnosis: ❑ Traumatic cartilage lesion ❑OD ❑OA ❑AVN ❑Others

Biopsy/Osteochondral Plugs: Location: Number of Plugs:

Diameter of Plugs: mm

Treatment:
❑ Shaving ❑ Abrasion
❑ Drilling ❑ Microfracture
❑ Osteochondral autograft transfer/mosaic-plasty
❑ Cell therapy ❑ Other

Notes:

ICRS Grade 0 - Normal

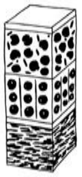

ICRS Grade 1 – Nearly Normal
Superficial lesions, Soft indentation (A) and/or superficial fissures and cracks (B)

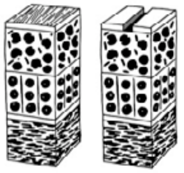

A B

ICRS Grade 2 – Abnormal
Lesions extending down to <50% of cartilage depth

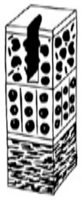

ICRS Grade 3 - Severely Abnormal
Cartilage defects extending down >50% of cartilage depth (A) as well as down to calcified layer (B) and down to Blisters are included in this Grade (D)

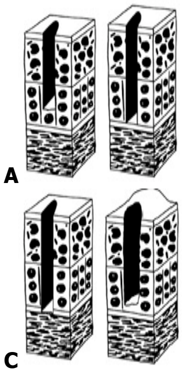

A B C D

ICRS Grade 4 – Severely Abnormal
Osteochondral injuries, lesions extending just through the subchondral boneplate (A) or deeper defects down into trabecular bone (B). Defects that have been drilled are regarded as osteochondral defects and classified as ICRS-C.

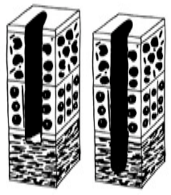

A B

MENISCUS STATUS:

Procedure:
- ❑medial meniscectomy ❑lateral meniscectomy
- ❑medial meniscal repair ❑lateral meniscus repair
- ❑medial meniscal transplant ❑lateral meniscal transplant
- ❑medial abrade & trephine ❑lateral abrade & trephine

Right Knee **Left Knee**

Document tears of the menisci or meniscectomy on these figures

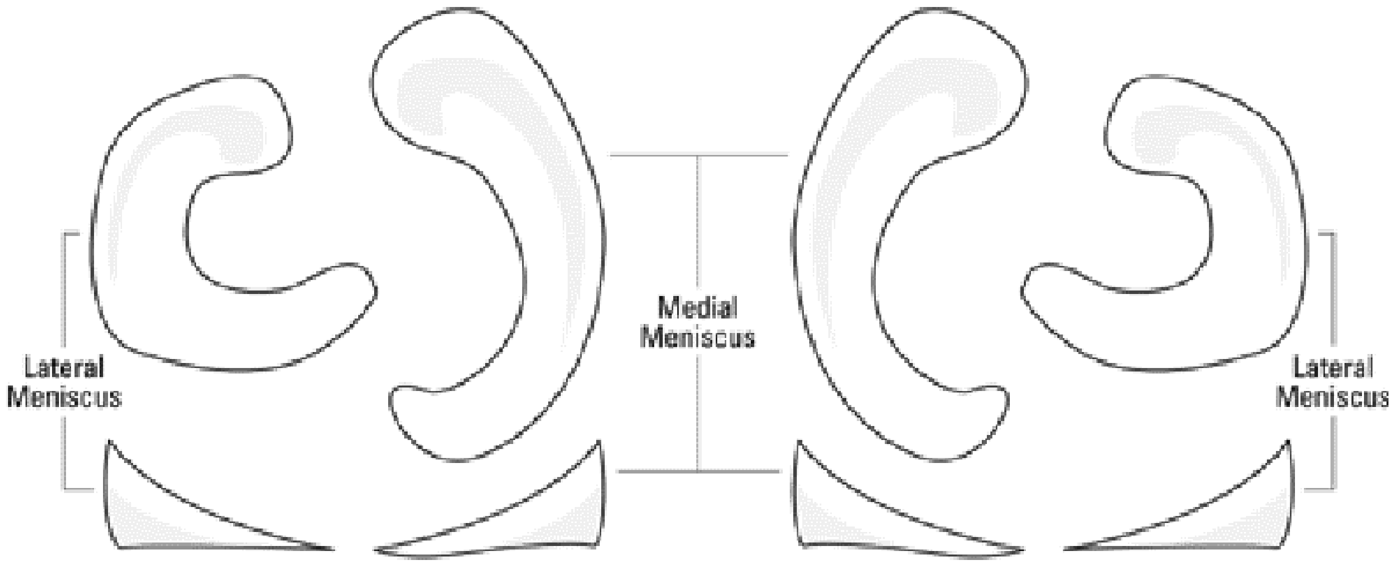

Medial:

❑Normal ❑1/3 Removed ❑2/3 Removed ❑3/3 Removed

Circumferential Hoop Fibers: ❑Intact ❑Disrupted

Remaining Meniscal Tissue: ❑Normal ❑Degenerative changes
❑Stable tear ❑Unstable tear
❑Tear left in situ

Lateral:

❑Normal ❑1/3 Removed ❑2/3 Removed ❑3/3 Removed

Circumferential Hoop Fibers: ❑Intact ❑Disrupted

Remaining Meniscal Tissue: ❑Normal ❑Degenerative changes
❑Stable tear ❑Unstable tear
❑Tear left in situ

LIGAMENT STATUS:

Procedure:

- ❑ACL repair ❑Intraarticular ACL reconstruction ❑Extraarticular ACL reconstruction
- ❑PCL repair ❑Intraarticular PCL reconstruction ❑Posterolateral corner repair/reconstruction
- ❑Medial collateral ligament repair/reconstruction
- ❑Lateral collateral ligament repair/reconstruction

Graft:

- ❑Autologous patella tendon ❑Hamstring tendons ❑Quadriceps tendon
- ❑Other_____________

Previous Graft Harvest:

- ❑Autologous patella tendon ❑Hamstring tendons ❑Quadriceps tendon

Document drill hole placement for ligament reconstruction on these figures.

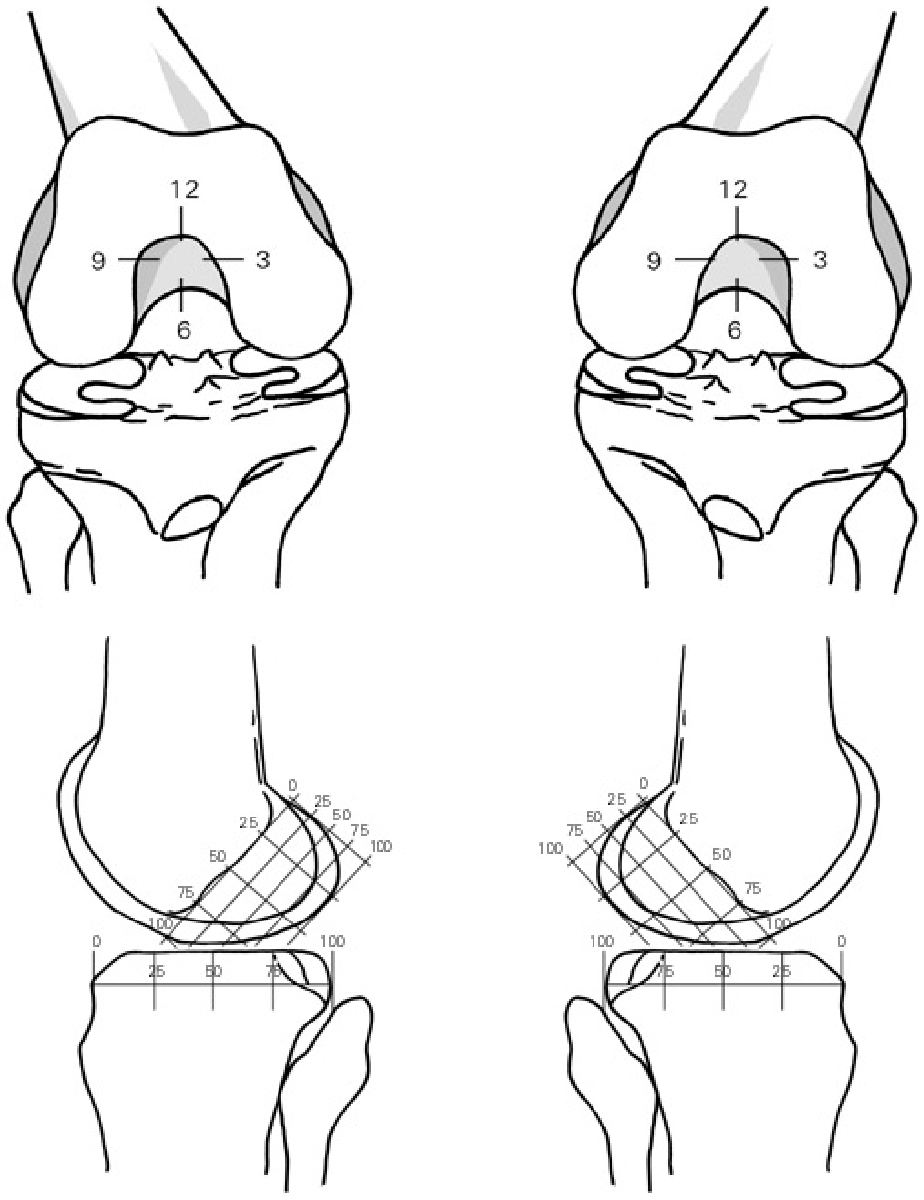

IKDC KNEE EXAMINATION FORM

Patient Initial : ___ ___ ___ **Date of Birth :** ___ ___ / ___ ___ / ___ ___ ___ ___
Day Month Year

Gender: ·F ·M **Age:**____________ **Date of Examination:**______/______/______
Day Month Year

**Generalized Laxity:**	·tight	·normal	·lax	
**Alignment:**	·obvious varus	·normal	·obvious valgus	
**Patella Position:**	·obvious baja	·normal	·obvious alta	
**Patella Subluxation/Dislocation:**	·centered	·subluxable	·subluxed	·dislocated

Range of Motion (Ext/Flex):
Index Side: passive______/______/______ active_____/_____/_____
Opposite Side: passive______/______/______ active_____/_____/_____

**SEVEN GROUPS**	**FOUR GRADES**<br>**A**<br>**Normal**	**B**<br>**Nearly Normal**	**C**<br>**Abnormal**	**D**<br>**Severely Abnormal**	***Group Grade**<br>**A**	**B**	**C**	**D**
**1. Effusion**	· None	· Mild	· Moderate	· Severe	·	·	·	·
**2. Passive Motion Deficit**								
ΔLack of extension	· <3°	· 3 to 5°	· 6 to 10°	· >10°				
ΔLack of flexion	· 0 to 5°	· 6 to 15°	· 16 to 25°	· >25°	·	·	·	·
**3. Ligament Examination** (manual, instrumented, x-ray)								
ΔLachman (25° flex) (134N)	· -1 to 2mm	· 3 to 5mm(1+)<br>· <-1 to –3	· 6 to 10mm(2+)<br>· <-3 stiff	· >10mm(3+)				
ΔLachman (25° flex) manual max	· -1 to 2mm	· 3 to 5mm	· 6 to 10mm	· >10mm				
Anterior endpoint:	· firm		· soft					
ΔTotal AP Translation (25° flex)	· 0 to 2mm	· 3 to 5mm	· 6 to 10mm	· >10mm				
ΔTotal AP Translation (70° flex)	· 0 to 2mm	· 3 to 5mm	· 6 to 10mm	· >10mm				
ΔPosterior Drawer Test (70° flex)	· 0 to 2mm	· 3 to 5mm	· 6 to 10mm	· >10mm				
ΔMed Joint Opening (20° flex/valgus rot)	· 0 to 2mm	· 3 to 5mm	· 6 to 10mm	·>10mm				
ΔLat Joint Opening (20° flex/varus rot)	· 0 to 2mm	· 3 to 5mm	· 6 to 10mm	· >10mm				
ΔExternal Rotation Test (30° flex prone)	· <5°	· 6 to 10°	· 11 to 19°	· >20°				
ΔExternal Rotation Test (90° flex prone)	· <5°	· 6 to 10°	· 11 to 19°	· >20°				
ΔPivot Shift	· equal	· +glide	· ++(clunk)	· +++(gross)				
ΔReverse Pivot Shift	· equal	· glide	· gross	· marked	·	·	·	·
**4. Compartment Findings**			crepitation with					
ΔCrepitus Ant. Compartment	· none	· moderate	· mild pain	· >mild pain				
ΔCrepitus Med. Compartment	· none	· moderate	· mild pain	· >mild pain				
ΔCrepitus Lat. Compartment	· none	· moderate	· mild pain	· >mild pain				
**5. Harvest Site Pathology**	· none	· mild	· moderate	· severe				
**6. X-ray Findings**								
Med. Joint Space	· none	· mild	· moderate	· severe				
Lat. Joint Space	· none	· mild	· moderate	· severe				
Patellofemoral	· none	· mild	· moderate	·severe				
Ant. Joint Space (sagittal)	· none	· mild	· moderate	· severe				
Post. Joint Space (sagittal)	· none	· mild	· moderate	· severe				
**7. Functional Test**								
One Leg Hop (% of opposite side)	· ≥90%	· 89 to 76%	· 75 to 50%	· <50%				
****Final Evaluation**					·	·	·	·

* Group grade: The lowest grade within a group determines the group grade
** Final evaluation: the worst group grade determines the final evaluation for acute and subacute patients. For chronic patients compare preoperative and postoperative evaluations. In a final evaluation only the first 3 groups are evaluated but all groups must be documented. Δ Difference in involved knee compared to normal or what is assumed to be normal.

INSTRUCTIONS FOR THE IKDC KNEE EXAMINATION FORM

The Knee Examination Form contains items that fall into one of seven measurement domains. However, only the first three of these domains are graded. The seven domains assessed by the Knee Examination Form are:

1. Effusion
   An effusion is assessed by ballotting the knee. A fluid wave (less than 25 cc) is graded mild, easily ballotteable fluid – moderate (25-60 cc), and a tense knee secondary to effusion (greater than 60 cc) is rated severe.

2. Passive Motion Deficit
   Passive range of motion is measured with a gonimeter and recorded on the form for the index side and opposite or normal side. Record values for zero point/hyperextension/flexion (e.g. 10 degrees of hyperextension, 150 degrees of flexion = 10/0/150; 10 degrees of flexion to 150 degrees of flexion = 0/10/150). Extension is compared to that of the normal knee.

3. Ligament Examination
   The Lachman test, total AP translation at 70 degrees, and medial and lateral joint opening may be assessed with manual, instrumented or stress x-ray examination. Only one should be graded, preferably a "measured displacement". A force of 134 N (30 lbs) and the maximum manual are recorded in instrumented examination of both knees. Only the measured displacement at the standard force of 134 N is used for grading. The numerical values for the side to side difference are rounded off, and the appropriate box is marked.

   The end point is assessed in the Lachman test. The end point affects the grading when the index knee has 3-5 mm more anterior laxity than the normal knee. In this case, a soft end point results in an abnormal grade rather than a nearly normal grade.

   The 70-degree posterior sag is estimated by comparing the profile of the injured knee to the normal knee and palpating the medial femoral tibial stepoff. It may be confirmed by noting that contraction of the quadriceps pulls the tibia anteriorly.

   The external rotation tests are performed with the patient prone and the knee flexed 30° and 70°.
   Equal external rotational torque is applied to both feet and the degree of external rotation is recorded.

   The pivot shift and reverse pivot shift are performed with the patient supine, with the hip in 10-20 degrees of abduction and the tibia in neutral rotation using either the Losee, Noyes, or Jakob techniques. The greatest subluxation, compared to the normal knee, should be recorded.

4. Compartment Findings
   Patellofemoral crepitation is elicited by extension against slight resistance. Medial and lateral compartment crepitation is elicited by extending the knee from a flexed position with a varus stress and then a valgus stress (i.e., McMurray test). Grading is based on intensity and pain.

5. Harvest Site Pathology
   Note tenderness, irritation or numbness at the autograft harvest site.

6. X-ray Findings
   A bilateral, double leg PA weightbearing roentgenogram at 35-45 degrees of flexion (tunnel view) is used to evaluate narrowing of the medial and lateral joint spaces. The Merchant view at 45 degrees is used to document patellofemoral narrowing. A mild grade indicates minimal changes (i.e., small osteophytes, slight sclerosis or flattening of the femoral condyle) and narrowing of the joint space which is just detectable. A moderate grade may have those changes and joint space narrowing (e.g., a joint space of 2-4 mm side or up to 50% joint space narrowing). Severe changes include a joint space of less than 2 mm or greater than 50% joint space narrowing.

7. Functional Test
   The patient is asked to perform a one leg hop for distance on the index and normal side. Three trials for each leg are recorded and averaged. A ratio of the index to normal knee is calculated.
