# Supplementary material for: Effect of upper limb isometric training (ULIT) on hamstring strength in early postoperative anterior cruciate ligament reconstruction patients: Study protocol for a randomized controlled trial
Source: PLoS One. 2025 Aug 21;20(8):e0319724. doi: 10.1371/journal.pone.0319724 (PMC12370102; doi:10.1371/journal.pone.0319724)
Supplement: S5 Appendix — (PDF) [file pone.0319724.s005.pdf]

S5 Appendix. Upper Limb Isometric Training (ULIT)

Instruction for Upper Limb Isometric Training

General instructions for all exercises:

Finding suitable wall and floor: A solid wall made of concrete, brick, or drywall supported by a stud frame and without sharp edges. Ensures the wall can withstand the force you apply during the exercise without moving or causing uneven pressure. The floor in front of the wall should have good traction, like a rubber mat or non-slip tiles.

Standing Position: Stand upright with feet shoulder-width apart. Ensure your lower back and hip maintain a neutral position, knee in full extension and equal pressure over the bilateral foot throughout each exercise.

Effort Level: Begin the first repetition with 100% effort. For the remaining 5 repetitions, perform each exercise at 50% of your maximum effort.

Repetition Structure: Hold each position for 5 seconds, followed by 5 seconds of rest. Complete a total of 5 repetitions for each exercise. Perform 1 set of 5 repetitions, 1-2x session /day.

Rest: Relax your upper limb muscles and step back slightly to rest for 1 minute before continuing the next exercise.

Additional Tips:

Breathing: Keep your breathing steady and controlled throughout the exercise. Avoid holding your breath.

Modification: If maintaining the position is challenging, you can step slightly closer to the wall to reduce the intensity.

Focus: Concentrate on keeping your body straight and avoiding any movement or sagging in your lower back during the hold.

When to Stop:

Discomfort or Pain: If you feel any sharp pain or discomfort in your shoulders, back, or arms, stop the exercise immediately.

Fatigue: If you feel excessively fatigued or your form begins to deteriorate.

Breathing Difficulty: If you struggle to maintain controlled breathing, take a break and reassess your effort level.

**Exercise 1: Isometric Wall Push-Up**	
Starting Position:<br>Place your palm flat on the wall at shoulder height, slightly wider than shoulder-width apart.<br>Your forearms should be parallel to the floor, and your elbows should be 90 degrees bent at the side of your body.<br>Lean slightly forward, positioning yourself so your body forms a straight line from your head to your heels.<br>Ensure that your lower back stays neutral, avoiding any arching or rounding.<br><br>Movement:<br>Begin the exercise by exerting your effort into the wall through your hands, as if you were trying to push the wall away without moving your body.<br>Engage your chest, shoulders, triceps, back and core muscles to maintain the static position.<br>Your body should remain in a straight line, with your elbows at the side of your body.	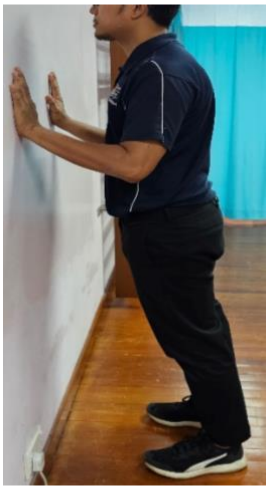
**Exercise 2: Isometric Shoulder Extension**	
Stating Position:<br>Stand close enough against the wall so that your elbows can press against the wall behind you.<br>Your elbows should remain bent at a 90-degree angle and tucked into your sides.<br>Keep your shoulders relaxed and down.<br>Ensure your spine stays in a neutral position, avoiding any arching or rounding in the lower back.<br><br>Movement:<br>Begin the exercise by exerting your effort into the wall through the back of your forearm, as if you were trying to push the wall away without moving your body.<br>Your body should remain in a straight line, with your elbows at the side of your body.	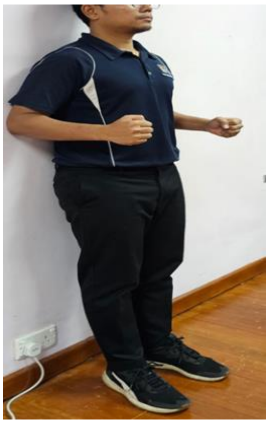
**Exercise 3: Isometric Shoulder External Rotation**	
Starting Position:<br>Stand with your side close to the wall, with the elbow closest to the wall bent at a 90-degree angle.<br>Your forearm should be slightly pronated and parallel to the floor.<br>Keep your elbow tucked into your side and your upper arm parallel to the floor.<br><br>Movement:<br>-Apply pressure by pushing your hand against the wall using the lateral aspect of your arm for external rotation without moving your arm.<br>- Maintain a neutral spine position and engage your core.<br>- After completing the repetitions on one side, switch to the other arm.	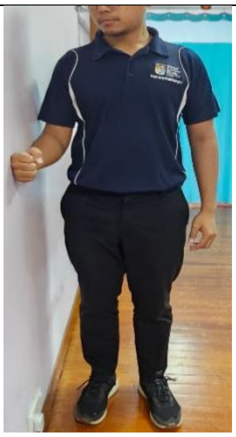
