# Supplementary material for: Effect of upper limb isometric training (ULIT) on hamstring strength in early postoperative anterior cruciate ligament reconstruction patients: Study protocol for a randomized controlled trial
Source: PLoS One. 2025 Aug 21;20(8):e0319724. doi: 10.1371/journal.pone.0319724 (PMC12370102; doi:10.1371/journal.pone.0319724)
Supplement: S6 Appendix — (PDF) [file pone.0319724.s006.pdf]

S6 Appendix. Anterior Cruciate Ligament Reconstruction (ACLR) Exercise Protocol.

HCTM

ANTERIOR CRUCIATE LIGAMENT RECONSTRUCTION

EXERCISE PROGRAM PROTOCOL

Overview

The protocol serves as a guideline for rehabilitation, outlining objectives and criteria for progression. The physician/ physiotherapy team recommends progression, based on team objectives, complications, and surgical findings.

The program is designed with flexibility in mind, presenting exercises in a progressive manner and organized into different stages.

It is recommended to plan a period each day for performing these exercises, which can be performed multiple times per day or at one time.

It is crucial to follow the patient's body lead and avoid continuing exercises that cause pain, especially if it lingers or swells significantly.

When there are concurrent injuries (i.e. meniscus repair), it is important to follow a more conservative protocol for range of motion, weight-bearing, and rehab progression.

In the absence of clear direction from the surgeon upon initial referral, there should be an automatic default, which should be confirmed or modified with the surgeon on subsequent communication:

- Meniscal repair: Non-weight bearing (NWB) for 4 weeks and braced 0° to 90°
- Chondral repair: NWB for 4 weeks and braced 0° to 90°
- Lateral Extra-articular Tenodesis: Weight bearing and ROM as tolerated
- Secondary ligament injuries (i.e posterolateral corner): NWB for 4 weeks and braced 0° to 90°

The baseline case would usually include two additional functional precautions:

- Kneeling Prayer: Modified from month 4, full knee flexion from month 6
- Driving: Right knee: 4-6 weeks // Left knee: 2-3 weeks

This table provides an overview of the **exercise progressions, suggested timelines and rehabilitation goals**. It is important to adapt the progression based on each individual's presentation.

PHASE 1	PHASE 2	PHASE 3	PHASE 4	PHASE 5
RECOVERY FROM SURGERY	INITIAL STRENGTH & NEUROMUSCULAR CONTROL	PROGRESSIVE STRENGTH & NEUROMUSCULAR CONTROL	RUNNING, AGILITY & LANDING	RETURN TO SPORT
0-2 weeks	2-6 weeks	6-12 weeks	3-6 month	9-12 month
• Full knee extension<br>• Minimize swelling<br>• Minimum of 0-90° of knee flexion<br>• Appropriate wound healing	• Progress to full weight-bearing (2 crutches → 1 crutch/cane → walking without support)<br>• walking without any support<br>• Stroke test ≤2<br>• Achieve 0–120° flexion<br>• Return to activities of daily living (ADLs)	• ½ depth single leg squat with appropriate joint mechanics<br>• Stroke test ≤1 (No change in pain/swelling the next morning)<br>• Full knee extension & flexion<br>• ≥70% quadriceps & hamstring LSI	• Completed sport-specific re-training<br>• Stroke test ≤0<br>• Leg symmetry index ≥ 90% (Quad strength, Hamstring strength, Single leg hop test, Triple hop test, Triple crossover test)	a minimum of 9 months back to sport if satisfied 3 key criteria:<br>•Successful completion of the Melbourne Return to Sport Score (>95)<br>•The athlete is comfortable, confident, and eager to return to sport measured by the ACL-RSI and IKDC<br>•An ACL injury prevention program is discussed and implemented.

	PHASE 1	PHASE 2	PHASE 3	PHASE 4	PHASE 5
**RANGE OF MOTION (ROM)**					
Heel Over Roll Stretch	√				
Heel Slides	√				
Knee Extension Isometric	√				
Knee Extension and Flexion in Sitting	√				
Prone Hangs		√			
Wall Slides		√			
Cycling on a Stationary Bike (very little or no resistance)		√			
Hamstrings Stretch		√			
Gastroc Stretch With Towel		√			
Standing Gastroc/Soleus Stretch		√			
**STRENGTHENING**					
Active Knee Flexion	√				
Seated Calf Raise	√				
Hip Abduction (Countertop)	√				
Hip Flexion AROM | Bent Knee (Chair)	√				
Hip Extension AROM (Chair)	√				
Knee Extension Concentric | End Range (Band+Chair)		√			
Hamstring Curls With Tubing While Sitting		√			
1/4 Double and Single Leg Squats with support		√			
Hip Adduction (Band)		√			
Hip Abduction (Band)		√			
Hip Extension (Band)		√			
Calf Raises		√			
Hamstring Curls in Sitting With Elastic Band (as previously)			√		
Standing Hamstring Curls			√		
Hamstring Curls Lying on Your Stomach			√		

Squat - Arms Forward			√		
Single Leg Squat | Straight Leg			√		
Double Leg Squats with Tubing			√		
Step-Ups and Step-Downs			√		
Bridging			√		
Bridging Single Leg			√		
Single Leg Calf Raises Without Support			√		
Deadlift | Single Leg and Arm (Dumbbell)			√		

	PHASE 1	PHASE 2	PHASE 3	PHASE 4	PHASE 5
**STRENGTHENING (Cont'd)**					
Knee Flexion Machine			√		
Knee Flexion Machine | Single Leg			√		
Leg Press (Machine)			√		
Runner's Step Up				√	
Reverse Lunge				√	
Lateral Lunge (Bosu)				√	
Lunge | Forward (Bosu)				√	
Monster Walk (Band)				√	
**BALANCE**					
One Legged Stance		√			
One leg on Cushion			√		
Balance Variations			√		
Single Leg Balance with Arm Movements			√		
Airplane			√		
Advanced Wobble Board				√	
Double Leg Use of Wobble Board				√	
Single Leg use of Wobble Board				√	
Two-legged Squats on Wobble Board				√	
Ball Toss (Wobble Board)				√	
**CARDIO**					
Walking - Gait Cycle		√			
Brisk Walking/Longer Walks/Stairs			√		
Walking Treadmill			√		
Swimming Strokes No Breaststroke/Whipkick, Eggbeater			√		
Water Running			√		
Elliptical			√		
Rowing Machine			√		
Jogging / Running				√	
Skip Rope				√	
**AGILITY**					
Icky Shuffle (Ladder)				√	
Two Foot Hop Forward				√	
Forward Run One Foot (Every Space)				√	
Two Feet In and Out (Every Space)				√	
Two Feet In and Out (Every Other Space)				√	

PHASE 1 RECOVERY FROM SURGERY

In phase 1 it is important to improve the range of motion (ROM) of your knee. **Getting your knee straight is the most important initial goal.** The early motion will reduce the risk of developing a stiff knee. Learning to activate the muscles on the top of the thigh, called the quadriceps, will helprestore and maintain the ROM.

Arrange a visit with a physiotherapist to ensure you understand the specific exercise of this phase.

STAGE 1 GOALS

- Full knee extension (straightening)
- Minimize swelling
- Minimum of 0-90 degrees of ROM
- Appropriate wound healing

PHASE 1 PATIENT EDUCATION

WEIGHT BEARING

- Weight bearing using your brace and crutches that allow quality movement within your pain tolerance.
- Start at about 50% and increase as tolerated to full weight bearing.
- The diagrams and descriptions below will outline how to properly navigate different environments.

WALKING | STEP-THROUGH (CRUTCHES)

Preparation:

- Make sure crutches are properly fitted

Execution:

- Crutches and operated leg come forward at the sametime
- Step-through with the non-operated leg

Tip:

- Squeeze the crutch handles against your ribs - Don't let them ride into your armpits

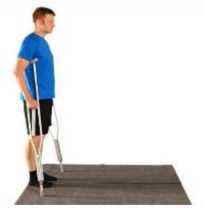

Make sure crutches are properly fitted

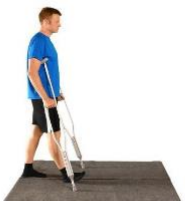

Crutches and operated leg come forward

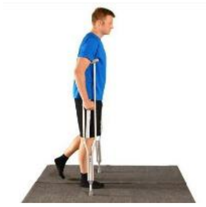

Step-through with non-operated leg

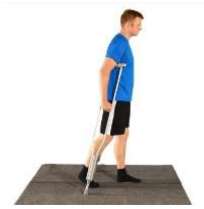

Repeat

PHASE 1 RECOVERY FROM SURGERY

STAIRS | ASCENDING (CRUTCHES + RAILING)

Preparation:

- Make sure crutches are fitted properly
- Both crutches on the side opposite the rail

Execution:

- Press down on crutches and rail to unweight affected leg
- Step up with strong leg first
- Bring operative leg and crutches up to meet strong leg

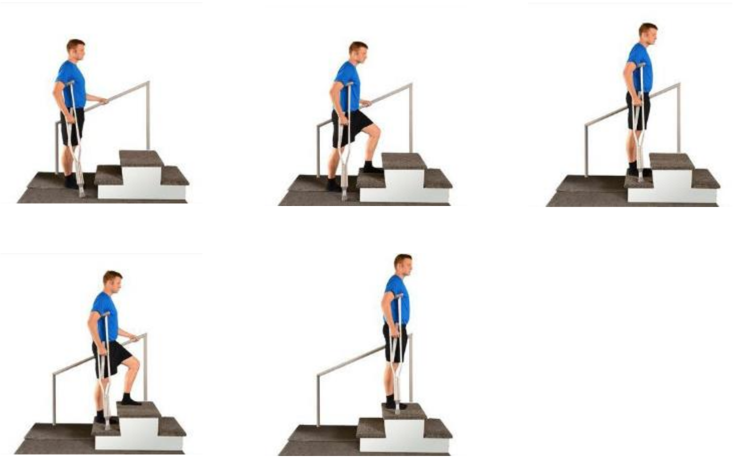

STAIRS | DESCENDING (CRUTCHES + RAILING)

Preparation:

- Make sure crutches are fitted properly
- Both crutches on the side opposite the rail

Execution:

- Step forward with operated leg and crutches
- Press down on crutches and rail to unweight operated leg
- Step down with strong leg

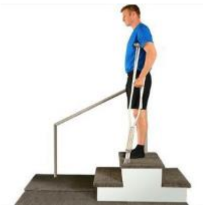

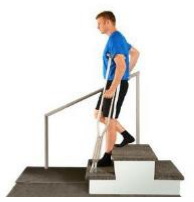

Step forward with operated leg and crutches

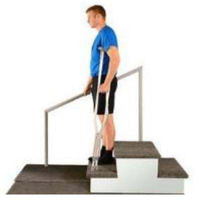

Use crutches to unweight operated leg and step down with strong leg

ICE / ELEVATION

Duration: 15-20 min | Frequency: 3-5x/day

- Ice pack applied immediately after surgery and used every 2 hours,especially after exercises.
- Operated knee should be elevated when icing and at rest.

PHASE 1 REHABILITATION EXERCISES

To maintain range of motion and prevent stiffness from developing, it is important to keep moving with the exercises below.

RANGE OF MOTION

HEEL OVER ROLL STRETCH

Sets: 5x/day | Duration: Start 2 min at a time, work up to 5 min

Preparation:

- Lay on back with knee straight
- Put a roll (ie: towel) under your ankle

Execution:

- Passively (ie: allowing gravity) stretch the knee into extension
- Start with 2 minutes at a time, working up to 2-5minutes as long as you are pain free

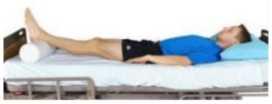

Place a roll (i.e. towel) under your ankle and passively stretch your knee

HEEL SLIDES

Sets: 5x/day | Reps: 5-10 | Hold: 5-10 sec

Preparation:

- Lay on back with knee straight

Execution:

- Keeping the heel in contact with the floor, gently pullthe heel of your operated leg towards you buttock
- You may use a towel to assist with bending your knee
- Relax back to the start position
- Use the opposite leg to assist straightening the knees

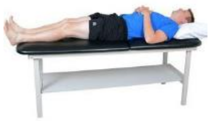

Start position

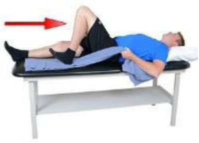

Gently pull towel to assist with bending your knee

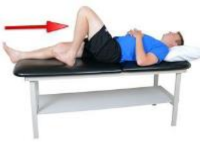

Slide heel up

KNEE EXTENSION ISOMETRIC

Sets: 5x/day | Reps: 15-20

Preparation:

- Lie flat on your back

Execution:

- Tighten the muscles on the top of your thigh bypushing the back of your knee into the floor andlifting your heel off the ground
- Relax

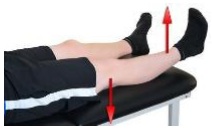

Push back of knee to ground, lift heel

PHASE 1 REHABILITATION EXERCISES

KNEE EXTENSION AND FLEXION IN SITTING

Sets: 5x/day | Reps: 5-10 | Hold: 5 sec

Preparation:
- Sit in a chair with the ankle of your operated legunder the ankle of your good foot

Execution:
- Gently pull your operated leg back, supported with your good leg, until you feel a stretch
- Use your good leg to assist with straightening the operated knee

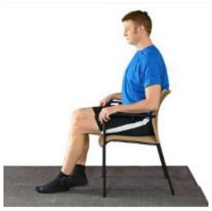
Start in a sitting position

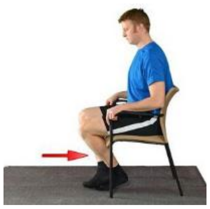
Gently pull your operated leg back, supported with your good leg, until you feel a stretch

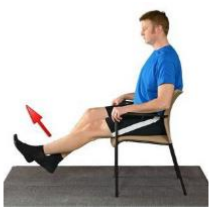
Use your good leg to assist with straightening the operated knee

STRENGTHENING

ACTIVE KNEE FLEXION

Sets: 5x/day | Reps: 20

Preparation:
- Lie on your stomach on a table or bed and position yourself so that your foot hangs off the edge of the bed

Execution:
- Bend your operated knee to raise your lower leg offof the surface
- Then slowly lower your leg, controlling this movement with your other leg
- Avoid letting your foot drop down

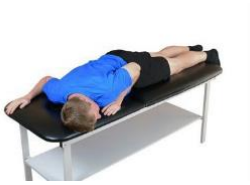
Lie on your stomach with your foot hanging off theedge of the bed

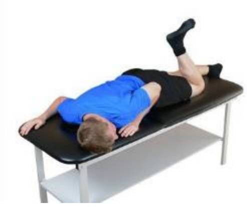
Slowly bend your knee up and down

SEATED CALF RAISE

Sets: 5x/day | Reps: 15

Preparation:
- Sit with good posture

Execution:
- Raise heels up
- Lower down with control

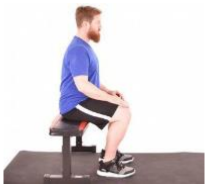
Start position

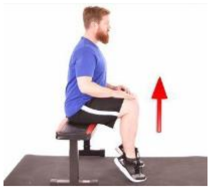
Raise heels up

PHASE 1 REHABILITATION EXERCISES

HIP ABDUCTION (COUNTERTOP)

Sets: 5x/day | Reps: 10

Preparation:
- Standing in front of a counter
- Hands placed firmly on the counter

Execution:
- Lift leg straight out to the side
- This exercise should be completed with both legs

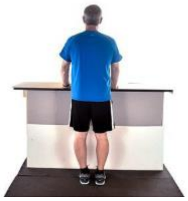
Hands firmly on the countertop for support

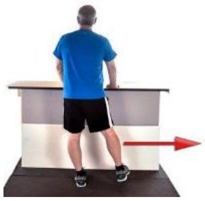
Leg straight out to the side

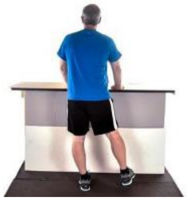
Trunk stays tall

HIP FLEXION AROM | BENT KNEE (CHAIR)

Sets: 5x/day | Reps: 10

Preparation:
- Standing next to a chair

Execution:
- Lift your knee up as if marching

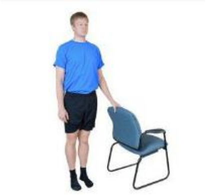
Standing with a chair for support

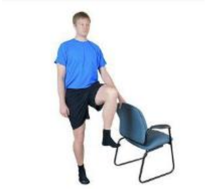
Lift bent knee up high, do not lean or bend trunk

HIP EXTENSION AROM (CHAIR)

Sets: 5x/day | Reps: 10

Preparation:
- Stand with good posture
- Use chair or counter to stabilize yourself

Execution:
- Lift one straight leg behind
- Return to the start position with control

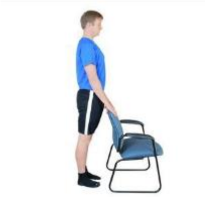
Start position

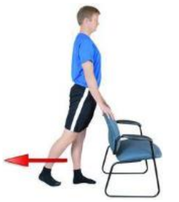
Lift leg behind

PHASE 1 MOVING ON TO PHASE 2

PHASE 1 OTHER CONSIDERATIONS

CRITERIA FOR PROGRESSION TO PHASE 2

1. No quads lag
2. Full Extension ROM (0-5 degree)
3. 90 degrees flexion ROM
4. Settle the swelling down to ‘mild (Stroke test 1+)

PHASE 2 INITIAL STRENGTH & NEUROMUSCULAR

The most important part of phase 2 is strengthening your quadriceps muscles and achieving full knee extension. Your knee extension range of motion should be the same as the non-operated knee.

Schedule appointments with a physiotherapist to make sure you grasp the details of this phase.

PHASE 2 GOALS

- Progress to full weight-bearing (use 2 crutches, then 1 crutch/cane (used on the opposite side), then walking without any support
- Maintain knee extension at 0 degrees
- Swelling reduction
- Achieve 0-120 degree range of motion
- Return to activities of daily living (ADLs)
- Return to office-type jobs

PHASE 2 PATIENT EDUCATION

WEIGHT-BEARING

- Progress towards full weight bearing (FWB) (i.e. walking without crutches). This may vary, depending on pain level, quadriceps control and functional ability (i.e. the ability to walk up/down stairs).
- In order to stop using crutches, you **must** be able to walk **without** a limp while using crutches (i.e. you must be able to fully weight bear on the operated leg **without** compensation).
- If you have also had a repair to your meniscus, you may need more time before full weight bearing
- Work on normalizing walking pattern

ICE / ELEVATION

Duration: 15-20 min | Frequency: 2-3x/day

- Monitor and control swelling.
- Use ice and/or compression after exercise and as required

PHASE 2 REHABILITATION EXERCISES

WALKING | GAIT CYCLE

1. Forward foot connects with the ground heel first
2. Forward foot flat to the ground
3. Shift weight forward - rise onto toes of back foot
4. Push off of back foot, swing back leg forward
5. Repeat

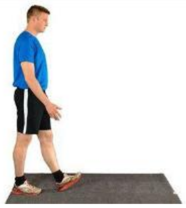

Forward foot connects with ground heel first

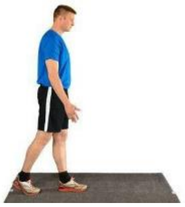

Forward foot flat to the ground

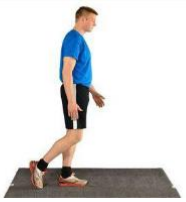

Shift weight forward- rise onto toes of back foot

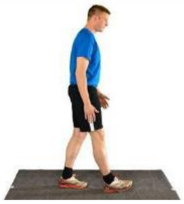

Push off with back foot, swing back leg forward

RANGE OF MOTION

PRONE HANGS

Sets: 4x/day | Reps: 5 | Hold: 20-30 sec

Preparation:

- Lie on your stomach on a table or bed, with your knees approximately 2 inches off the end of the bed

Execution:

- Slowly allow your operated knee to straighten and then hang

- Straightened position should be pain-free
- ADD: Add a heavy shoe or 1-2 kg weight at the ankle

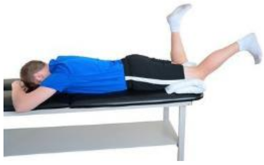

Lie on your stomach with your knees approximately 2” off the end of the bed/table

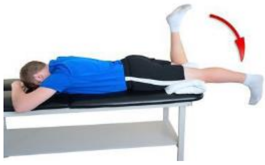

Slowly allow your operated knee to straighten and hang, pain-free

PHASE 2 REHABILITATION EXERCISES

WALL SLIDES

Sets: 4x/day | Reps: 10-15

Preparation:

- Lie on your back, on the floor or a bed that has oneend against the wall
- Place your feet on the wall with your knees almoststraight

Execution:

- Slowly allow your heels to slide down the wall, causing your knees to bend
- Use your non-operated leg to return the operated leg to the starting position

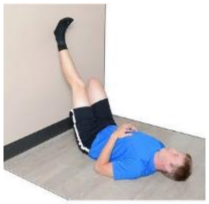

Place foot up on wall

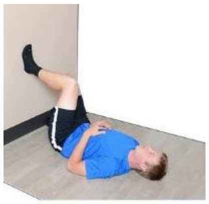

Slide foot down wall, bending knee

HAMSTRINGS STRETCH

Sets: 4x/day | Reps: 5 | Hold: 20-30 sec

Preparation:

- Lie flat on your back
- Use a towel, belt or strap to hold ankle

Execution:

- Keeping your knee straight, lift your leg up until youfeel a stretch in the back of the thigh
- Hold and then slowly lower the leg
- Alternate legs

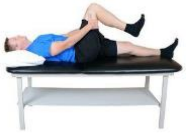

Lie flat on your back

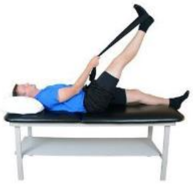

Lift your leg, keeping your knee straight, until you feel a stretch. Hold, then slowly lower leg.

GASTROC STRETCH WITH TOWEL

Sets: 4x/day | Reps: 5 | Hold: 20-30 sec

Preparation:

- Sit with your legs out in front of you
- Loop a towel or belt around the underside of yourfoot

Execution:

- Pull on the towel, drawing your toes towards you to stretch your calf muscles
- Do not bend your knee

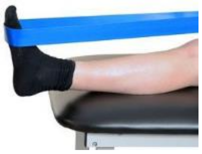

Sit with your legs out in front of you, with a towel/belt around the underside of your foot

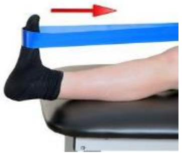

Pull on towel/belt bending ankle. Do not bend your knee.

PHASE 2 REHABILITATION EXERCISES

STANDING GASTROC/SOLEUS STRETCH

Sets: 4x/day | Reps: 5 | Hold: 20-30 sec

Preparation:
- Stand facing a wall

Execution:
- Lean against the wall first with your operated leg forward and knee bent
- **Gastroc Stretch:** Straighten the non-operated leg behind you, keeping a straight line from heel to head
- **Soleus Stretch:** Drop your back knee toward the ground
- Stretch out your calf muscles, ensuring that **both** heels stay on the floor. Hold.
- Then repeat with the operated leg
- Start with partial weight bearing on your operated leg and increase weight bearing as tolerated
- Ensure that you **do not hyperextend** your operated knee

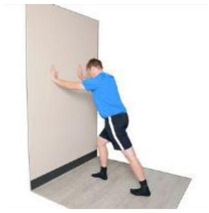

Gastroc Stretch: lean forward, back knee locked, heel on ground

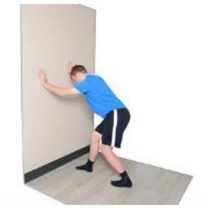

Soleus Stretch: drop back knee toward ground

STRENGTHENING

KNEE EXTENSION CONCENTRIC | END RANGE (BAND + CHAIR)

Sets: 4x/day | Reps: 10-15

Preparation:
- Loop band behind your knee slightly above the knee joint as shown
- Attach band to chair or door
- Stand with good posture, knee slightly bent

Execution:
- Straighten your knee as much as you can, pushing your bodyweight through your leg
- Relax your knee, returning to the start position

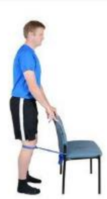

Knee slightly bent

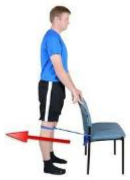

Straighten knee

PHASE 2 REHABILITATION EXERCISES

HAMSTRING CURLS WITH TUBING WHILE SITTING

Sets: 4x/day | Reps: 5-10

Perform all strengthening exercises on both legs so that your non-operated leg does not get weak.

Preparation:

- Sit on a chair
- Attach one end of the tubing to a secure, low-level support (i.e. approximately mid-shin height)
- Loop the other end of the tubing around your ankle

Execution:

- Use your hamstrings to bend your knee
- Return to the starting position and repeat

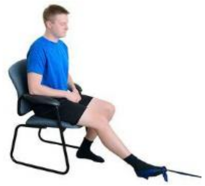

Sit in a chair with tubing looped around your ankle

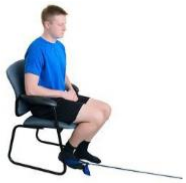

Use your hamstrings to bend your knee

1/4 DOUBLE AND SINGLE LEG SQUATS WITH SUPPORT

Sets: 4x/day | Reps: 10-20

Preparation:

- Stand with feet shoulder width apart
- Place your hands on a support (i.e. countertop), as required

Execution:

- Squat down like you are going to sit
- Only go partially down (range 0-40 degrees maximum) then return to standing position
- Work up to performing squats without **constant** support
- For all double leg squats, concentrate on equal weight bearing
- Start with 1/4 **double** leg squats, then progress to 1/4 **single** leg squats, as tolerated and using support as required

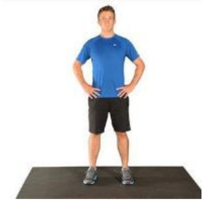

Double Leg Squat: stand with feet shoulder width apart

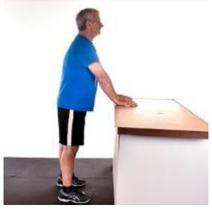

If required, place hands on a support with feet shoulder width apart

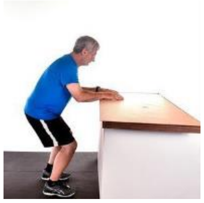

Squat down only partially, with equal weight bearing on both legs

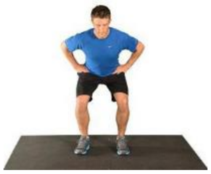

Work up to performing squats without constant support

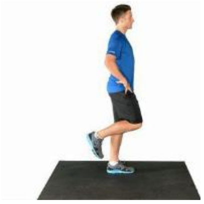

Single Leg Squat: stand with weight on one foot

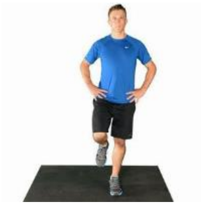

Squat down only partially

PHASE 2 REHABILITATION EXERCISES

HIP ADDUCTION (BAND)

Sets: 4x/day | Reps: 10-15

Preparation:

- Stand with band around thigh as shown
- Use chair to steady yourself

Execution:

- Bring legs together

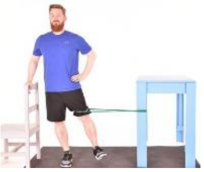

Start position

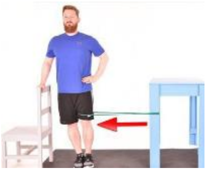

Bring legs together

HIP ABDUCTION (BAND)

Sets: 4x/day | Reps: 10-15

Preparation:

- Stand with band around thigh as shown
- Use table to steady yourself

Execution:

- Bring leg out to the side

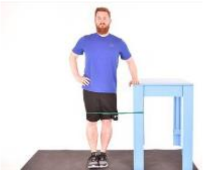

Start position

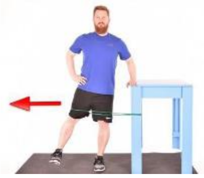

Bring leg out to side

HIP EXTENSION (BAND)

Sets: 4x/day | Reps: 10-15

Preparation:

- Stand with band around thigh as shown
- Use table to steady yourself

Execution:

- Lift leg behind you

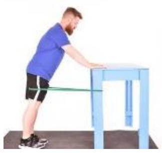

Start position

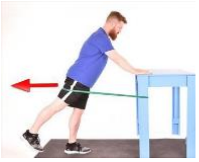

Lift leg behind you

CALF RAISES

Sets: 4x/day | Reps: 15-20

Progress from using both legs (bilateral) while using support, then to one leg at a time (unilateral) with support, then to bilateral without support.

Preparation:

- **For bilateral:** stand on both feet with your toes pointed straight ahead
- **For unilateral:** stand on one foot and bend your

  knee to hold up the other foot

Execution:

- Use your calf muscles to go up on your toes, lifting your heels off the ground, as high as you can
- Ensure a slow, controlled movement both up and down

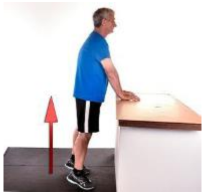

Bilateral calf raises with support. Stand on tip toes, then lower back down with control

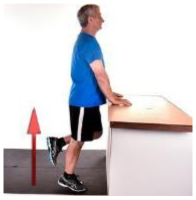

Unilateral calf raises with support. Stand on tip toes on one foot then lower back down with control

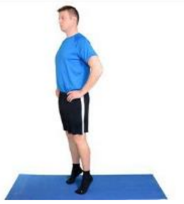

Bilateral calf raises without support. Stand on tip toes, then lower back down with control

PHASE 2 REHABILITATION EXERCISES

BALANCE

ONE LEGGED STANCE

Sets: 4x/day | Duration: 30 sec

The goal is to be able to balance comfortably and confidently on your operated knee.

Progress from standing on your operated leg (with support, as required) while looking down to standing while looking away, to performing an arm swing and then swinging the non-operated knee.

Progress further by performing these balanced activitieswith your eyes closed.

Preparation:

- Stand on your operated leg beside a support (i.e. countertop, wall)
- Start with fingertips on the countertop

Execution:

- Lift your non-operated leg
- Lift hand off the countertop
- Balance in this position

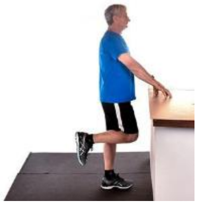

Stand on your operated leg with your fingertips on the countertop

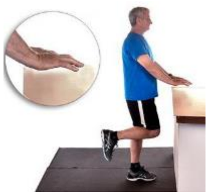

Balance on your operated leg with hand floating above the countertop

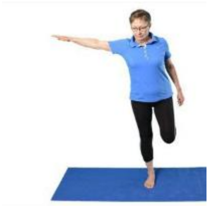

Balance on your operated leg while reaching to the side with your arm

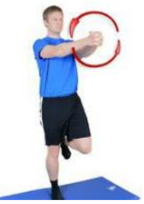

Progress to balancing on your operated leg while swinging arms

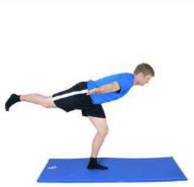

Balance on your operated leg while bending forward at your hip to make an airplane

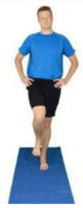

Balance on your operated leg with your eyes closed

CYCLING ON A STATIONARY BIKE (VERY LITTLE OR NO RESISTANCE)

Load: no resistance | Tempo: as able | Intensity: half circles

Duration: 10-20 min

- Use little or no resistance with the seat set higher than normal
- When getting on the bike, approach from you non-operated side. Be careful when mounting and dismounting the bike.
- Start with slow half circles forward and backwards
- Slowly work toward complete rotations, as you are able. You need between 105-110 degrees of flexion to make a full rotation.

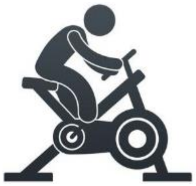

PHASE 2 MOVING ON TO PHASE 3

CRITERIA FOR PROGRESSION TO PHASE 3

1. Walk without a limp or gait aid
2. 120 degrees flexion
3. Balance on a single leg without assistance

PHASE 3 PROGRESSIVE STRENGTH & NEUROMUSCULAR CONTROL

The most important part of phase 3 is to perform all activities of daily living and to get back in gym for workouts (low-impact, controlled exercises).

Arrange visits with a physiotherapist to fully comprehend the details of this stage.

PHASE 3 GOALS

- Independent strengthening at the gym
- Eliminate swelling
- Achieve full range of motion
- Perform all activities of daily living (ADLs), example: stairs
- Return to work (except heavy labor or difficult environmental conditions)
- Clear to do upper body and core strengthening that do not involve pivoting to the knee

PHASE 3 PATIENT EDUCATION

ICE

- After exercise/activity and as required to control swelling

PHASE 3 REHABILITATION EXERCISES

CARDIO

During this stage, it is important to build up endurance for the knee and cardiovascular system.

Choose one of the suggested options below. Begin at a light intensity and progress as able. You may experience mild discomfort during exercise, but the pain levels should not increase during exercise. There should be no pain or swelling the following day.

A physiotherapist will be able to better guide you on the appropriate intensity and how to progress

BRISK WALKING/LONGER WALKS/STAIRS

- Full weight-bearing without limp, light cardio

WALKING TREADMILL

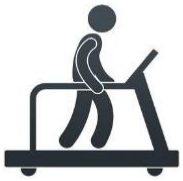

SWIMMING STROKES NO BREASTSTROKE/ WHIPKICK, EGGBEATER

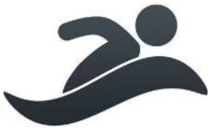

WATER RUNNING

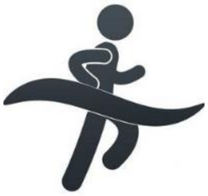

PHASE 3 REHABILITATION EXERCISES

ELLIPTICAL

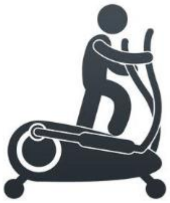

ROWING MACHINE

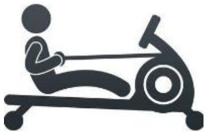

STRENGTHENING

Strengthening during this phase involves the use of external resistance in the form of weights, machines, and bands. By the end of the third set of exercises, you should begin to feel some muscular fatigue. At no point should your form become compromised. If you are unable to achieve the appropriate form after the third repetition, discontinue the exercise and try the following day. As we increase the load, you may need to give your muscles a break to recover. In such cases, it is recommended to take a day off of certain exercises.

Below you will find a variety of exercises to meet your strengthening goals. It is not recommended to do all the exercises in one session. Begin at a light intensity and progress as able. You may experience mild discomfort during exercise, but the pain levels should not increase during exercise. There should be no pain or swelling the following day.

HAMSTRING CURLS

Sets: 3 | Reps: 8-15

Perform all strengthening exercises on **both** legs so that your non-operated leg does not get weak.

You can alternate any of the following exercises for variety.

HAMSTRING CURLS IN SITTING WITH ELASTIC BAND

(as previously described)

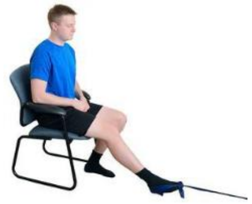

Hamstring Curls in Sitting with Tubing

STANDING HAMSTRING CURLS

Preparation:

- Attach one end of the band securely at heel height and attach the other end to your ankle
- Stand with good posture
- Use a table or chair for support

Execution:

- Actively bend your knee to lift your heel up towardsyour buttocks as far as you can
- **Slowly** return to the starting position and repeat
- Maintain pelvic tilt throughout

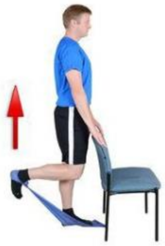

Standing Hamstring Curls: bend knee toward your buttocks, as far as you can. **Slowly** return to start.

HAMSTRING CURLS LYING ON YOUR STOMACH

Preparation:

- Attach one end of the band securely at heel height and attach the other end to your ankle
- Lie flat on your stomach with your legs straight

Execution:

- Actively bend your knee to lift your heel up towardsyour buttocks as far as you can
- **Slowly** return to the starting position and repeat
- Maintain pelvic tilt throughout

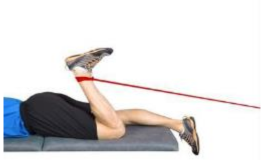

Hamstring Curls Lying on Stomach: bend knee toward buttocks. **Slowly** return to start.

SQUAT - ARMS FORWARD

Sets: 3 | Reps: 8-15

Preparation:
- Stand with good posture, feet shoulder-width apart
- Hold arms out in front at shoulder height

Execution:
- Perform a squat by bending at the hip
- Rise up by straightening at the hip

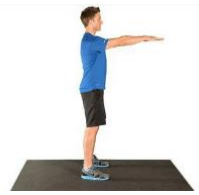
Start position

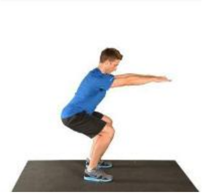
Squat - bend at the hip, back flat

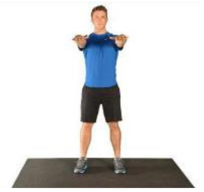
Knees aligned with toes

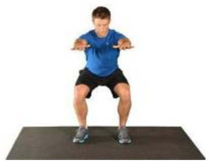
Finish position

SINGLE LEG SQUAT | STRAIGHT LEG

Sets: 3 | Reps: 8-15

Preparation:
- Stand with good posture on one leg

Execution:
- Begin to squat with one leg by bending at the hipand knee
- Keep the opposite leg straight
- Return to the start position in a controlled manner

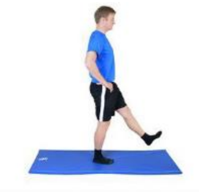
Start position

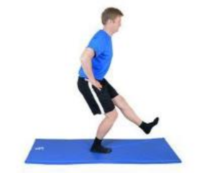
Squat - opposite leg straight forward

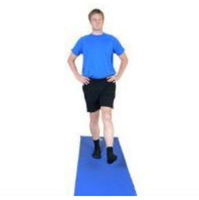
Front view

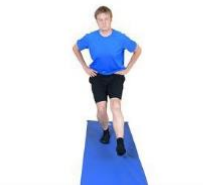
Knee in line with toes!

PHASE 3 REHABILITATION EXERCISES

DOUBLE LEG SQUATS WITH TUBING

Sets: 3 | Reps: 8-15

- Progress from 1/4 single-leg squats to 1/3 single-leg squats (as described previously). Squat deeper, but do not go beyond 90 degrees
- Progress to single-leg squats using hand weights or tubing for extra resistance. Progress to 1 minute per set and 6 sets with 1 minute rest between sets

DOUBLE LEG SQUAT:

- Increase the resistance of double-leg squats withhand weights or tubing
- Hold hand weights (dumbbells, plastic bottles filledwith water or sand) in your hands
- Place tubing under both feet and hold it in your hands. Adjust the tension of the band so that you are stretching the tubing as you push upwards

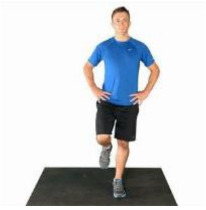

Progress to 1/3 Single Leg Squat. Squat deeper, but not beyond 90 degrees.

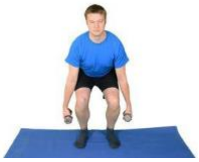

Double Leg Squat with hand weights

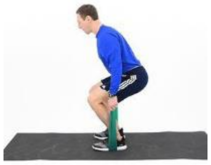

Double Leg Squat with tubing looped under feet

STEP-UPS AND STEP-DOWNS

Sets: 3 | Reps: 8-15

Step-Downs, Forward and Lateral Step-Up Exercises:

- At graduated heights starting at 4” and increasing to < 6”
- Increase the number of steps as tolerated

Preparation:

- Stand next to the box or step
- Hands resting at sides, pull belly button in

Execution:

- Step up with one leg, follow with the other
- Step down with the first leg
- Complete the repetition by stepping both feet down

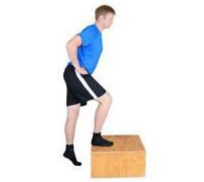

Forward Step-Ups: Step onto box

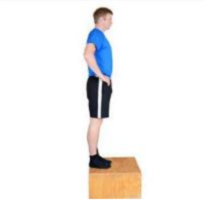

Rise on box with control

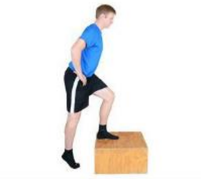

Step-Downs: Step back down from box

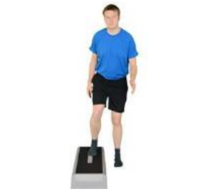

Lateral Step-Ups: Step onto box, one foot at a time, then back down

BRIDGING

Sets: 3 | Reps: 8-15

Preparation:

- Lie flat on your back with your arms straight besideyou
- Bend knees to 45 degrees

Execution:

- Use the muscles in your buttocks and the backs ofyour thighs to lift your hips off the floor
- Tighten your abdominal muscles for support
- Hold for 10 seconds and then lower down in acontrolled manner

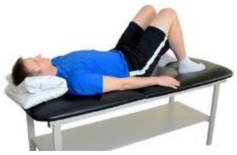

Lie on your back with your arms straight beside you and knees bent to 45 degrees

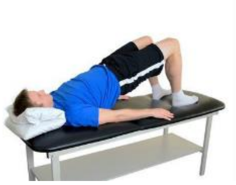

Use the muscles in your buttocks and backs of your thighs to lift your hips off the floor

PHASE 3 REHABILITATION EXERCISES

BRIDGING SINGLE LEG

Sets: 3 | Reps: 8-15

- Progress to single leg as able, can also have operated leg do 60%, 70%, etc. if unable to perform a complete single leg bridge. Focus on engaging your glute muscles

Bridging (as previously described):

- Raise buttocks from bed/floor

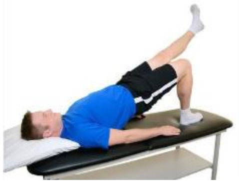

Bridging while lifting knee: alternate raising your knees

SINGLE LEG CALF RAISES WITHOUT SUPPORT

Sets: 3 | Reps: 8-15

Progress from double leg to single leg calf raises without support.

Preparation:

- Stand on one leg next to a chair, counter or wall

Execution:

- Stand on your tip toes, lifting your heel as high as you can
- Relax your heel back down to the ground

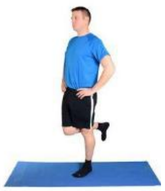

Stand on one leg

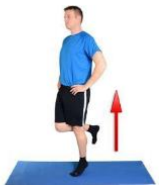

Rise onto tip toes as high as you can, then relax your heel back down to the ground

DEADLIFT | SINGLE LEG AND ARM (DUMBBELL)

Sets: 3 | Reps: 8-15

Preparation:

- Stand on one leg, dumbbell in the opposite hand asthe stance leg

Execution:

- Bend at the hip, keeping the trunk aligned with theback leg
- Rise up from the hips

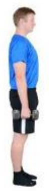

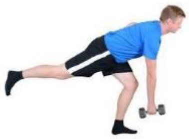

The knee will always stay aligned over the toes

KNEE FLEXION MACHINE

Sets: 3 | Reps: 8-15

Preparation:

- Sit with good posture as shown

Execution:

- Bend legs against resistance

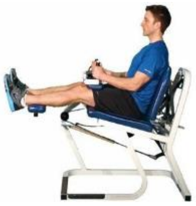

Start position

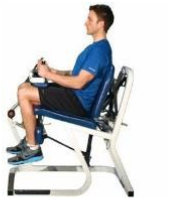

Finish position

PHASE 3 REHABILITATION EXERCISES

QUAD BENCH MACHINE | SINGLE LEG

Sets: 3 | Reps: 8-15

Preparation:

- Sit with good posture as shown

Execution:

- Bend one leg against resistance

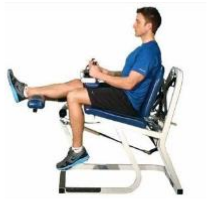

Start position

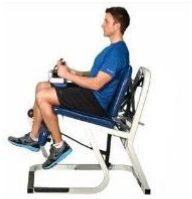

Finish position

LEG PRESS (MACHINE)

Sets: 3 | Reps: 8-15

Preparation:

- Sit with good posture as shown

Execution:

- Push weight away from body while straightening knees

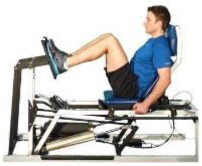

Start position

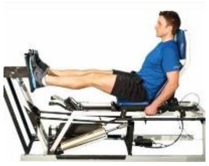

Finish position

BALANCE

When completing the balance exercises, it is normal for your knee to shake. Your goal is to limitthe amount of movement at the knee and maintain good upper-body posture. It is important to ensure your knee does not collapse inwards or rotate during the movements.

CUSHION

Sets: 3-5 | Duration: 30 sec

Preparation:

- Stand on a small pillow/cushion near a wall/ table top

Execution:

- Maintain balance on the wobble board
- Avoid touching the edges to the ground as long aspossible

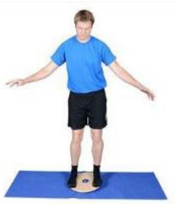

Balance on wobble board

PHASE 3 REHABILITATION EXERCISES

BALANCE VARIATIONS

Sets: 3-5 | Duration: 30 sec

Start with the first exercise, progressing to the others in the order listed, as you are able.

Do not perform balance exercises in **socks** - use either bare feet or sneakers to avoid slipping.

- Weight shifting forward / backward / laterally (as previously described)
- Continue with single leg balance exercises while standing on your operated leg (as previously described)

1. Perform arm circles in both directions
2. Raise your non-operated knee and swing it back and forth
3. Alternate leaning your upper body forward, backwards and to each side while maintaining your balance
4. Perform exercises while moving your head, with eyes closed and/or on different surfaces

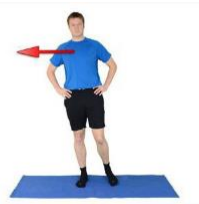

Lateral Weight Shifting Without Support: shift weight to one side, then shift to the other

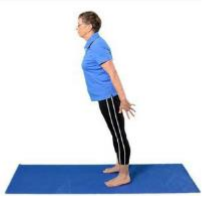

Forward/Backward Weight Shifting: stand with feet shoulder width apart, lean forward then return

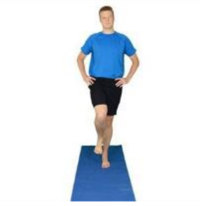

Single Leg Balance Exercises: stand on your operated leg

SINGLE LEG BALANCE WITH ARM MOVEMENTS

Sets: 3-5 | Duration: 30 sec

Preparation:

- Stand with good posture

Execution:

- Balance on one leg
- Clasp hands together
- In a controlled fashion, draw wide circles with your arms

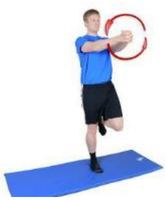

Balance on one leg, wave arms

AIRPLANE

Sets: 3-5 | Reps: 10

Preparation:

- Stand with good posture

Execution:

- Bend forward at the hip standing on one leg
- Keep the back leg and spine straight

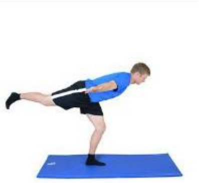

Make an airplane

PHASE 3 MOVING ON TO PHASE 4

Outcome Measure	Test Description & Reference	Goal	√
Passive Knee Extension	Prone hang test (Sachs et al, 1989)<br>Subjects lie prone on a treatment bed with the lower legs off the end allowing full passive knee extension. The heel height difference is measured (approx 1cm = 1°	Equal to the other side	○
Passive Knee Flexion	Supine with a long arm goniometer (Norkin & White, 1995).<br>Bony landmarks: greater trochanter, the lateral femoral condyle, and the lateral mallelous.	125^{+}	○
Swelling/ Effusion	Stroke Test (Sturgill et al, 2009)<br>Zero: No wave produced on downstroke<br>Trace: Small wave on medial side with downstroke<br>1+: Large bulge on medial side with downstroke<br>2+: Effusion spontaneously returns to medial side after upstroke<br>3+: So much fluid that it is not possible to move the effusion out of the medial aspect of the knee	Zero	○
Functional Alignment Test	Single leg squat test (Crossley et al, 2011)<br>Subjects stand on one leg on a 20cm box with arms crossed. 5 x single leg squats are performed in a slow controlled manner (at a rate of 2 seconds per squat). The task is rated as “good”, “fair” or “poor”.<br>For a subject to be rated “good”;<br>• Maintain balance<br>• Perform the movement smoothly<br>• Squat must be to at least 60 degrees<br>• No trunk movement (lateral deviation, rotation, lateral flexion, forward flexion)<br>• No pelvic movement (shunt or lateral deviation, rotation, or tilt)<br>• No hip adduction or internal rotation<br>• No knee valgus<br>• Centre of knee remains over the center of foot	Good	○

PHASE 3 MOVING ON TO PHASE 4

CRITERIA FOR PROGRESSION TO PHASE 4

1. Successfully complete ½ depth single leg squat
2. Appropriate joint mechanics with closed kinetic chain exercises (Squats, lunges, etc)
3. No/trace effusion
4. Full knee extension and 125° knee flexion
5. No change in pain or swelling next morning
6. Neutral pelvis in frontal plane at midstance
7. 70% quadriceps and hamstring strength LSI

PHASE 4 RUNNING, AGILITY & LANDING

The most important part of phase 4 is to improve single-leg balance, control and agility, and to return to in-line impact activities.

Arrange visits with a physiotherapist to ensure that you understand the specificsof this stage.

PHASE 4 GOALS

- Maintain full ROM - should be equal to the non-operated leg
- Continue strengthening
- Improve balance
- Improve agility

PHASE 4 PATIENT EDUCATION

ICE

Duration: 15-20 min | Frequency: as needed

- After exercise/activity and as required to control swelling

PHASE 4 RUNNING, AGILITY & LANDING

CARDIO

JOGGING/RUNNING

- Start power walking
- Progress to jogging against tubing resistance (forward/backward) - increase time, resistance and speed gradually
- Increase distance and speed (avoid steep terrain, < 3% grade on treadmill)

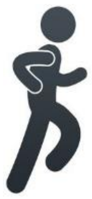

SKIP ROPE

Duration: as tolerated

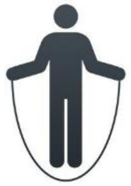

STRENGTHENING

RUNNER’S STEP UP

Sets: 3 | Reps: 15+ | Intensity: 6” then increase height

Preparation:

- Stand in front of a box or step that is below the levelof the knee

Execution:

- Step up onto the box and bring the opposite leg up towards your chest
- Lower down in a controlled manner
- Repeat, alternating legs

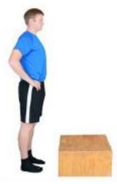

Start position

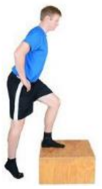

Keep knee in line with toes

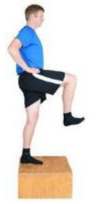

Rise with control, lifting opposite leg

PHASE 4 RUNNING, AGILITY & LANDING

REVERSE LUNGE

Sets: 3 | Reps: 8-15

Preparation:
- Stand with good posture

Execution:
- Lunge backwards
- Return to start position or continue lunging backwards

Note:
- Keep your front knee aligned with your middle toe

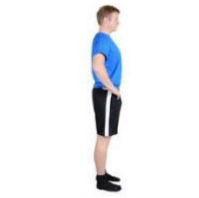

Start position

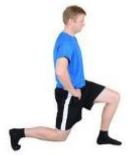

Lunge backward

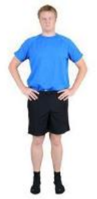

Front view

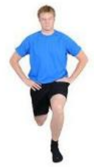

Knee aligned with toes!

LATERAL LUNGE (BOSU)

Sets: 3 | Reps: 8-15

Preparation:
- Stand with good posture

Execution:
- Lunge to the side on to the blue side of Bosu
- Keep your hip and knee aligned with your middle toe

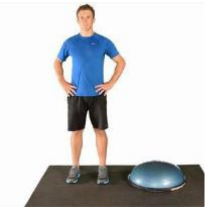

Start position

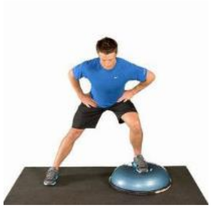

Keep hip and knee in line with foot

LUNGE | FORWARD (BOSU)

Sets: 3 | Reps: 8-15

Preparation:
- Stand with good posture

Execution:
- Lunge forward, keeping your front knee aligned with your middle toe
- Rise up and step forward with the opposite foot, repeating the motion

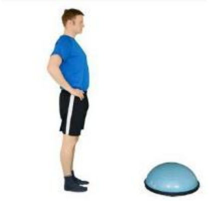

Lunge

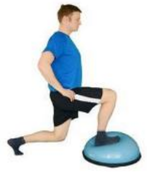

Rise up, lunge with opposite foot

PHASE 4 RUNNING, AGILITY & LANDING

MONSTER WALK (BAND)

Sets: 3 | Duration: 30 sec

Preparation:
- Attach tubing to ankles as shown
- Wide stance, knees and hips slightly bent

Execution:
- Perfom a partial squat
- Walk forward, keeping legs wide
- Keep tension on tubing throughout the whole exercise

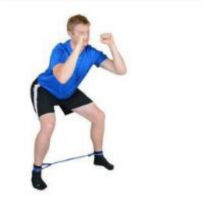

Wide stance

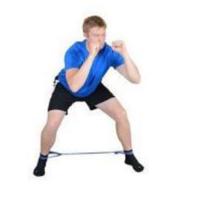

Walk wide - keep tubing under tension

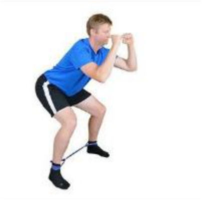

BALANCE

ADVANCED WOBBLE BOARD

Sets: 5 | Duration: 30 sec | Frequency: daily

Double Leg Use of Wobble Board:
- Move side to side, then front to back (i.e. 4 points ofthe compass)
  - Ensure there is no rotation
  - Progress from feet wide to feet close
  - Control is goal
- Try to keep the edges of the board off the floor for

  10 seconds, increasing this time as you are able

Single Leg use of Wobble Board
- Progress to single leg
- As previously described, progress to balancing and performing controlled movement with one leg instead of two
- Place the foot in the middle of the board
- Perform the same activities as for Double Leg Use

Two-legged Squats on Wobble Board
- 10 sets daily
- Start with your feet shoulder width apart
- Try to keep the edges of the wobble board off theground while performing the squats

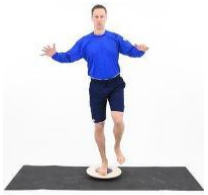

Single Leg Use of Wobble Board: try to keep edges off floor for 10 seconds

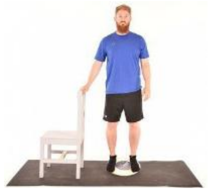

Double Leg Use of Wobble Board: move side to side, front to back

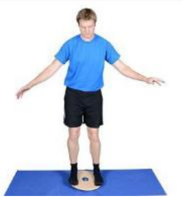

Double Leg Use of Wobble Board: try to keep edges off floor for 10 seconds

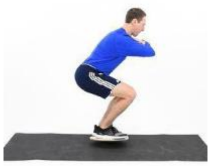

Squats on Wobble Board: squat while keeping edges off floor

PHASE 4 RUNNING, AGILITY & LANDING

BALL TOSS (WOBBLE BOARD)

Sets: 3 | Reps: 8-15

Preparation:
- Stand on a wobble board

Execution:
- Toss ball against the wall

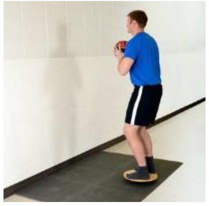

Stand on a wobble board

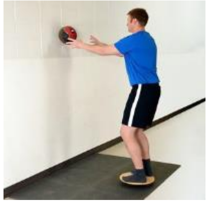

Toss ball against wall

AGILITY

ICKY SHUFFLE (LADDER)

Frequency: 3-5x/week

Preparation:
- Stand on one side of the ladder

Foot Pattern:
- Shuffle into the first space with one foot
- Follow by bringing the other foot in and quickly shuffling the first foot out
- Repeat throughout the length of the ladder

Remember:
- Step as quickly as possible

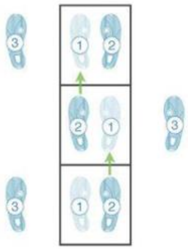

TWO FOOT HOP FORWARD

Frequency: 3-5x/week

Preparation:
- Start facing the ladder with feet shoulder width apart

Foot Pattern:
- Hop forward, landing both feet in the first space
- Immediately hop forward, landing both feet in the second space
- Repeat through the whole ladder

Remember:
- Hop as quickly as possible
- Absorb the shock of the landing by bending at the hips, knees and ankles

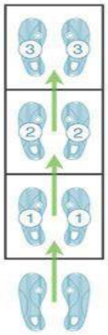

PHASE 4 RUNNING, AGILITY & LANDING

FORWARD RUN ONE FOOT (EVERY SPACE)

Frequency: 3-5x/week

Preparation:
- Stand facing the ladder

Foot Pattern:
- Run forward, landing one foot into each space
- Continue using the full length of the ladder

Remember:
- Step as quickly as possible
- Touch the floor only using the ball of your foot

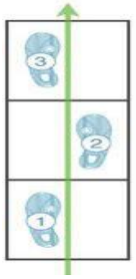

TWO FEET IN AND OUT (EVERY SPACE)

Frequency: 3-5x/week

Preparation:
- Start with feet apart straddling either side of the ladder

Foot Pattern:
- Jump and land with feet together inside the first space
- Jump and land with feet apart landing on either side of the first rung
- Repeat throughout the length of the ladder

Remember:
- Absorb the shock of the landing by bending at the hips, knees and ankles

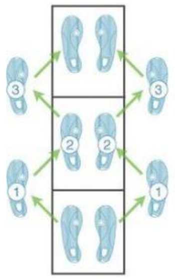

TWO FEET IN AND OUT (EVERY OTHER SPACE)

Frequency: 3-5x/week

Preparation:
- Start with feet apart straddling either side of the ladder

Foot Pattern:
- Jump up and land with feet together inside the firstspace
- Jump up and land with feet apart on the outside ofthe second space
- Repeat throughout the length of the ladder

Remember:
- Absorb the shock of the landing by bending at the hips, knees and ankles

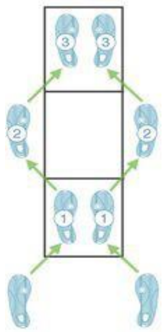

Phase 4: Outcome Measures and Goal

The following hurdle criteria must be met before the testing is conducted:

- Full range of motion (prone hang test and knee flexion)
- No effusion/swelling (stroke test)
- A "good" rating on the Single Leg Squat Test

Outcome Measure	Test Description & Reference	Goal	√
Single Hop Test	Single leg hop test (Noyes et al, 1991)<br>Subjects stand on one leg and hop as far forward as possible and land on the same leg. The average (mean) distance of 2 valid hops is recorded with a tape measure which is fixed to the ground. Measure from toe at take-off to heel at landing. Arms are free to swing.<br>A limb symmetry index is calculated by dividing the mean distance (in cms) of the involved limb by the mean distance of the noninvolved limb then multiplying by 100.	**1.** >90% compared with other side<br>**2.** Equal to or greater than pre-operative data (best result – affected or non-affected)	○
Triple Hop	Triple Hop Test (Noyes et al., 1991)<br>Subjects are required to hop forwards three consecutive times on one foot. The total distance is measured, and the average (mean) of 2 valid tests is recorded. Measure from toe at take off to heel at landing. Arms are free to swing.<br>A limb symmetry index is calculated by dividing the mean distance (in cms) of the involved limb by the mean distance of the noninvolved limb then multiplying by 100.	>90% compared with other side	○
Triple Cross Over Hop Test	Triple Cross Over Hop Test (Noyes et al, 1991)<br>This test is performed on a course consisting of a 15cm marking strip on the floor which is 6m long. Subjects are required to hop three consecutive times on one foot going in a medial to lateral to medial direction, crossing the strip on each hop. The total distance is measured, and the average (mean) of 2 valid hop tests is recorded. Measure from toe at take-off to heel at landing. Arms are free to swing.<br>A limb symmetry index is calculated by dividing the mean distance (in cms) of the involved limb by the mean distance of the noninvolved limb then multiplying by 100.	>90% compared with other side	○

PHASE 4 RUNNING, AGILITY & LANDING

Outcome Measure	Test Description & Reference	Goal	√
Side Hop Test	Side Hop Test (Gustavsson et al., 2006)<br>Subjects stands on test leg with hands behind the back and jumps from side to side between two parallel strips of tape, placed 40 cm apart on the floor.<br>Subject jumps as many times as possible during 30sec. The number of successful jumps performed, without touching the tape is recorded.	>90% compared with other side	○
Single Leg Squat	Single Leg Rise Test (Culvenor et al., 2016 & Thorstensson et al., 2004)<br>Subjects sit on a chair (or a plinth) with test leg bent to 90°, and 10cm from edge of chair.<br>With hands behind the back, the subject aims to stand up from the sitting position, and sit down as many times as possible.	Hurdle requirement = >22 repetitions both limbs	○
Balance (Dynamic)	Star Excursion Balance Test (Gribble et al, 2012)<br>The star excursion balance test (SEBT) is performed in the anterior, posterolateral, and posteromedial directions.<br>A composite score for all 3 directions is obtained for each leg. A limb symmetry index is then calculated by dividing the mean distance (in cms) of the involved limb by the mean distance of the noninvolved limb then multiplying by 100.	>90% compared with other side	○
Balance (Dynamic)	Cooper & Hughes Sports Vestibular Balance Test<br>Subjects stand on one leg with a small amount of flexion in the hip, knee and ankle, and place their hands on their waist.<br>In this position, two assessments are performed;<br>1. Side to side<br>At a rate of 60 beats per minute, subjects repeatedly turn their head from side to side (70-90 degree turn) for a period of 15 seconds. Vision needs to be inline with head position (no visual fixing).<br>2. Up and down<br>At a rate of 60 beats per minute, subjects repeatedly tilt their head up and down (looking floor to ceiling) for a period of 15 seconds. Vision needs to be inline with head position (no visual fixing).<br>The test is passed if subjects can maintain single leg stance and do not take their hands off their waist for both assessments	Pass both limbs	○

PHASE 5 RETURN TO SPORT

CRITERIA FOR PROGRESSION TO PHASE 5

1. **Completed sport specific re-training**
2. **Completed a gradual return to running**
3. **Leg symmetry index over 90%**
    - Quad strength
    - Hamstring strength
    - Single leg hop test
    - Triple hop test
    - Triple crossover test

PHASE 5 RETURN TO SPORT

You have completed the first four stages of your rehabilitation; however, the health of your knee requires more work if you want to return to sport. The most important part of phase 5 is to ensure your progress is done safely and gradually.

It is best to expose yourself to activity-specific movements slowly. While it is normal to fear reinjury, it is necessary to simulate game-like scenarios to develop confidence in unpredictable situations. Managing your risk of re-injury is essential to achieving a successful return to sport. You may be able to safely return to sport but be aware that returning to your pre-injury performance level will take time.

Risk factors for re-injury:

- Low hamstring strength compared to the quadriceps muscle
- Quadriceps strength asymmetry (operated vs. non-op)
- Poor hop performance (single hop for distance, triple hop for distance, triple crossover fordistance)
- Time from surgery (< 9 months)
- Insufficient sport specific training

PHASE 5 OTHER CONSIDERATIONS

Key components of an injury prevention program should include:

- Plyometric, balance, and strengthening exercises
- That the program must be performed for at least 10mins before every training session and game
- That the program is ongoing

Five popular injury prevention programs that include exercises to help reduce ACL injuries are available on the web. Five programs are:

- Sportsmetrics Program
- The 11+ Warm Up: http://f-marc.com/11plus/home/
- The PEP Program: https://www.youtube.com/watch?v=7Lag8uNU6AQ
- The KNEE Program - Netball Australia
- The FootyFirst Program - AFL

It is highly recommended that athletes continue with an ongoing ACL injury prevention program whilst they continue to play sports

PHASE 5 RETURN TO SPORT

So, when are people ready to return to sport after an ACL reconstruction?

Current research suggests a **minimum of 9 months**, however, please be guided by your surgeon and sports medicine team. Our suggestion is to allow people back to sport if they have satisfied 3 key criteria:

- Successful completion of **the Melbourne Return to Sport Score (>95)**
- The athlete is comfortable, confident, and eager to return to sport, as measured by the ACL-RSI and IKDC
- An ACL injury prevention program is discussed, implemented, and continued whilst the athlete is participating in sport. To lower the risk of future injury, evidence supports that **injury prevention programs are performed at least 15 minutes** before each training session and game.

The Melbourne Return to Sport Score is an assessment tool for returning to sport following anterior cruciate ligament reconstruction. There are six components to the test:

- Clinical Examination (10 points)
- IKDC Subjective Knee Evaluation and ACL-RSI (20 points)
- Tampa Scale of Kinesiophobia (hurdle criteria)
- Functional Testing (50 points)
- Assessment of General Fitness (hurdle criteria)
- Functional Testing in a Fatigued State (20 points)

We suggest performing the tests over two sessions at least 3 days apart.

- Session 1 – Part A, B, C, & D
- Session 2 – Part E & F

Melbourne Return to Sport Scoring Sheet

Part A: Stability, Swelling, & Range

Item	Result	Score	
**Effusion**		/5	
**Stability**		/5	
**Flexion**		/5	
**Extension**		/5	**Converted**
		/20	/10

Part B: IKDC Subjective Knee Evaluation Form & ACL-RSI

Item	Result	Converted	
**ACL-RSI**	/100	/10	
**IKDC**	/100	/10	**Converted**
		/20	/20

Part C: Tampa Scale of Kinesiophobia (TSK-11)

Item	Score	Result	
**TSK-11**	19 or more	Fail	
	11 - 18	Pass	**Pass / Fail**

Part D: Functional Testing

Item	Result	Score	
**SEBT**		/10	
**Vestibular Balance**		/10	
**Single Hop**		/5	
**Triple Hop**		/5	
**Triple Crossover**		/5	
**Side Hop**		/5	
**SL Rise**		/10	**Total**
			/50

Part E: General Fitness Testing

Item	Result	
Test 1		
Test 2		Pass / Fail

Part F: Functional Testing in a Fatigued State

Item	Result	Score	
Single Hop		/5	
Triple Hop		/5	
Triple Crossover		/5	
Side Hop		/5	Total
			/20

Final Score
/100

PHASE 5 RETURN TO SPORT

Part A: Stability, Swelling, & Range

Test	Outcome	Points Awarded
Effusion	Absent	5 Points
	Present	0 Points
Stability (Pivot Shift Test)	Nil	5 Points
	Grade I	3 Points
	Grade II	1 Points
	Grade III-IV	0 Points
Flexion	0-5 degrees deficit	5 Points
	5-20 degrees deficit	3 Points
	20+ degrees deficit	0 Points
Extension (Prone Hang Test)	0-1cm deficit	5 Points
	1-5cm deficit	3 Points
	5cm+ deficit	0 Points

Total
/20

PHASE 5 RETURN TO SPORT

Part B: ACL-RSI

1. Are you confident that you can perform at your previous level of sport participation?

Not at all confident	0 ☐	10 ☐	20 ☐	30 ☐	40 ☐	50 ☐	60 ☐	70 ☐	80 ☐	90 ☐	100 ☐	Fully confident

2. Do you think you are likely to re-injury your knee by participating in your sport?

Extremely likely	0 ☐	10 ☐	20 ☐	30 ☐	40 ☐	50 ☐	60 ☐	70 ☐	80 ☐	90 ☐	100 ☐	Not likely at all

3. Are you nervous about playing your sport?

Extremely nervous	0 ☐	10 ☐	20 ☐	30 ☐	40 ☐	50 ☐	60 ☐	70 ☐	80 ☐	90 ☐	100 ☐	Not nervous at all

4. Are you confident that your knee will not give way by playing your sport?

Not at all confident	0 ☐	10 ☐	20 ☐	30 ☐	40 ☐	50 ☐	60 ☐	70 ☐	80 ☐	90 ☐	100 ☐	Fully confident

5. Are you confident that you could play your sport without concern for your knee?

Not at all confident	0 ☐	10 ☐	20 ☐	30 ☐	40 ☐	50 ☐	60 ☐	70 ☐	80 ☐	90 ☐	100 ☐	Fully confident

6. Do you find it frustrating to have to consider your knee with respect to your sport?

Extremely frustrating	0 ☐	10 ☐	20 ☐	30 ☐	40 ☐	50 ☐	60 ☐	70 ☐	80 ☐	90 ☐	100 ☐	Not at all frustrating

7. Are you fearful of re-injuring your knee by playing your sport?

Extremely fearful	0 ☐	10 ☐	20 ☐	30 ☐	40 ☐	50 ☐	60 ☐	70 ☐	80 ☐	90 ☐	100 ☐	Not fear at all

Part B: ACL-RSI

8. Are you confident about your knee holding up under pressure?

	0	10	20	30	40	50	60	70	80	90	100	
Not at all confident	☐	☐	☐	☐	☐	☐	☐	☐	☐	☐	☐	Fully confident

9. Are you afraid of accidentally injuring your knee by playing your sport?

	0	10	20	30	40	50	60	70	80	90	100	
Extremely afraid	☐	☐	☐	☐	☐	☐	☐	☐	☐	☐	☐	Not at all afraid

10. Do thoughts of having to go through surgery and rehabilitation prevent you from playing your sport?

	0	10	20	30	40	50	60	70	80	90	100	
All of the time	☐	☐	☐	☐	☐	☐	☐	☐	☐	☐	☐	None of the time

11. Are you confident about your ability to perform well at your sport?

	0	10	20	30	40	50	60	70	80	90	100	
Not at all confident	☐	☐	☐	☐	☐	☐	☐	☐	☐	☐	☐	Fully confident

12. Do you feel relaxed about playing your sport?

	0	10	20	30	40	50	60	70	80	90	100	
Not at all relaxed	☐	☐	☐	☐	☐	☐	☐	☐	☐	☐	☐	Fully relaxed

Test	Outcome	Points Awarded
**ACL RSI**	**> 90% = 10/10**	10 Points
	**< 90% = 0/10**	0 Points

**Total**
/10

Reference: Webster et al, 2008

Part B: IKDC Subjective Knee Evaluation Form

Test	Outcome		Points Awarded
**IKDC**	**Raw score**	**/100**	
	**Divide by 10**		/10 Points

Reference: Anderson et al, 2006

SYMPTOMS*:

*Grade symptoms at the highest activity level at which you think you could function without significant symptoms, even if you are not actually performing activities at this level.

1. What is the highest level of activity that you can perform without significant knee pain?

4 ☐ Very strenuous activities like jumping or pivoting as in basketball or soccer
3 ☐ Strenuous activities like heavy physical work, skiing or tennis
2 ☐ Moderate activities like moderate physical work, running or jogging
1 ☐ Light activities like walking, housework or yard work
0 ☐ Unable to perform any of the above activities due to knee pain

2. During the past 4 weeks, or since your injury, how often have you had pain?

	0	1	2	3	4	5	6	7	8	9	10	
Never	☐	☐	☐	☐	☐	☐	☐	☐	☐	☐	☐	Constant

3. If you have pain, how severe is it?

	0	1	2	3	4	5	6	7	8	9	10	
No pain	☐	☐	☐	☐	☐	☐	☐	☐	☐	☐	☐	Worst pain imaginable

4. During the past 4 weeks, or since your injury, how stiff or swollen was your knee?

4 ☐ Not at all
3 ☐ Mildly
2 ☐ Moderately
1 ☐ Very
0 ☐ Extremely

5. What is the highest level of activity you can perform without significant swelling in your knee?

4 ☐ Very strenuous activities like jumping or pivoting as in basketball or soccer
3 ☐ Strenuous activities like heavy physical work, skiing or tennis
2 ☐ Moderate activities like moderate physical work, running or jogging
1 ☐ Light activities like walking, housework, or yard work
0 ☐ Unable to perform any of the above activities due to knee swelling

Part B: IKDC Subjective Knee Evaluation Form

6. During the past 4 weeks, or since your injury, did your knee lock or catch?

0 ☐ Yes 1 ☐ No

7. What is the highest level of activity you can perform without significant giving way in your knee?

4 ☐ Very strenuous activities like jumping or pivoting as in basketball or soccer
3 ☐ Strenuous activities like heavy physical work, skiing or tennis
2 ☐ Moderate activities like moderate physical work, running or jogging
1 ☐ Light activities like walking, housework or yard work
0 ☐ Unable to perform any of the above activities due to giving way of the knee

Sports Activities:

8. What is the highest level of activity you can participate in on a regular basis?

4 ☐ Very strenuous activities like jumping or pivoting as in basketball or soccer
3 ☐ Strenuous activities like heavy physical work, skiing or tennis
2 ☐ Moderate activities like moderate physical work, running or jogging
1 ☐ Light activities like walking, housework or yard work
0 ☐ Unable to perform any of the above activities due to knee

9. How does your knee affect your ability to:

		Not difficult at all	Minimally difficult	Moderately difficult	Extremely difficult	Unable to do
a.	Go up stairs	4 ☐	3 ☐	2 ☐	1 ☐	0 ☐
b.	Go down stairs	4 ☐	3 ☐	2 ☐	1 ☐	0 ☐
c.	Kneel on the front of your knee	4 ☐	3 ☐	2 ☐	1 ☐	0 ☐
d.	Squat	4 ☐	3 ☐	2 ☐	1 ☐	0 ☐
e.	Sit with your knee bent	4 ☐	3 ☐	2 ☐	1 ☐	0 ☐
f.	Rise from a chair	4 ☐	3 ☐	2 ☐	1 ☐	0 ☐
g.	Run straight ahead	4 ☐	3 ☐	2 ☐	1 ☐	0 ☐
h.	Jump and land on your involved leg	4 ☐	3 ☐	2 ☐	1 ☐	0 ☐
i.	Stop and start quickly	4 ☐	3 ☐	2 ☐	1 ☐	0 ☐

Part B: IKDC Subjective Knee Evaluation Form

FUNCTION:

10. How would you rate the function of your knee on a scale of 0 to 10 with 10 being normal, excellent function and 0 being the inability to perform any of your usual daily activities which may include sports?

Function Prior To Your Knee Injury:

Couldn't perform daily activities	0	1	2	3	4	5	6	7	8	9	10	No limitation in daily activities
	☐	☐	☐	☐	☐	☐	☐	☐	☐	☐	☐	

Current Function Of Your Knee:

Can't perform daily activities	0	1	2	3	4	5	6	7	8	9	10	No limitation in daily activities
	☐	☐	☐	☐	☐	☐	☐	☐	☐	☐	☐	

Scoring Instructions for the 2000 IKDC Subjective Knee Evaluation Form

Several methods of scoring the IKDC Subjective Knee Evaluation Form were investigated. The results indicated that summing the scores for each item performed as well as more sophisticated scoring methods.

The responses to each item are scored using an ordinal method such that a score of 0 is given to responses that represent the lowest level of function or highest level of symptoms. For

example, item 1, which is related to the highest level of activity without significant pain is scored by assigning a score of 0 to the response "Unable to perform any of the above activities due to knee pain" and a score of 4 to the response "Very strenuous activities like jumping or pivoting as in basketball or soccer". For item 2, which is related to the frequency of pain over the past 4 weeks, the responses are reverse-scored such that "Constant" is assigned a score of 0 and "Never" is assigned a score of 10.
Similarly, for item 3, the responses are reversed-scored such that "Worst pain imaginable" is assigned a score of 0 and "No pain" is assigned a score of 10. Note: previous versions of the form had a minimum item score of 1 (for example, ranging from 1 to 11). In the most recent version, all items now have a minimum score of 0 (for example, 0 to 10). To score these prior versions, you would need to transform each item to the scaling for the current version.The IKDC Subjective Knee Evaluation Form is scored by summing the scores for the individual items and then transforming the score to a scale that ranges from 0 to
100. **Note:** The response to item 10a "Function Prior to Knee Injury" is not included in the overall score. To score the current form of the IKDC, simply add the score for each item (the small number by each item checked) and divide by the maximum possible score which is 87:

Thus, for the current version, if the sum of scores for the 18 items is 45 and the patient responded to all the items, the IKDC.

Score would be calculated as follows:

$$\text{IKDC Score} = \frac{45}{87} \times 100 \qquad \text{IKDC Score} = 51.7$$

The transformed score is interpreted as a measure of function such that higher scores represent higher levels of function and lower levels of symptoms. A score of 100 is interpreted to mean no limitation with activities of daily living or sports activities and the absence of symptoms.The IKDC Subjective Knee Form score can be calculated when there are responses to at least 90% of the items (i.e. when responses have been provided for at least 16 items). In the original scoring instructions for the IKDC Subjective Knee Form, missing values are replaced by the average score of the items that have been answered. However, this method could slightly over- or under-estimate the score depending on the maximum value of the missing item(s) (2, 5 or 11 points).
Therefore, in the revised scoring procedure for the current version of a form with up to two missing values, the IKDC Subjective Knee Form Score is calculated as (sum of the completed items) / (maximum possible sum of the completed items) * 100. This method of scoring the IKDC Subjective Knee Form is more accurate than the original scoring method.

A scoring spreadsheet is also available at: www.sportsmed.org/research/index.asp This spreadsheet uses the current form scores and the revised scoring method for calculating scores with missing values.

$$\text{IKDC Score} = \frac{\text{Sum of Items}}{\text{Maximum Possible Score}} \times 100$$

Total
/10

Part C: Tampa Scale of Kinesiophobia (TSK-11)

People who fail the TSK-11 should not continue with the remainder of the MRSS2.0 testing as return to sport should not be considered. Further time and rehabilitation is required.

		Strongly disagree	Somewhat disagree	Somewhat agree	Strongly agree
1	I'm afraid that I might injure myself if I exercise.	1	2	3	4
2	If I were to try to overcome it, my pain would increase.	1	2	3	4
3	My body is telling me I have something dangerously wrong.	1	2	3	4
4	People aren't taking my medical condition seriously enough.	1	2	3	4
5	My accident has put my body at risk for the rest of my life.	1	2	3	4
6	Pain always means I have injured my body.	1	2	3	4
7	Simply being careful that I do not make any unnecessary movements is the safest thing I can do to prevent my pain from worsening.	1	2	3	4
8	I wouldn't have this much pain if there weren't something potentially dangerous going on in my body.	1	2	3	4
9	Pain lets me know when to stop exercising so that I do not injure myself.	1	2	3	4
10	I can't do all the things normal people do because it's too easy for me to get injured.	1	2	3	4
11	No one should have to exercise when he/she is in pain.	1	2	3	4

Test	Outcome	Result	
TSK-11	11 - 18	Pass	
	> 18	Fail	Pass / Fail

Reference: Woby et al, 2005

PHASE 5 RETURN TO SPORT

Part D: Functional Testing

Star Excursion Balance Test

	Right	Left	LSI	Points
**Anterior**			%	/5
**Posteromedial**				
**Posterolateral**			LSI	Points
**Total**			%	/5

Cooper & Hughes Vestibular Balance Test

Item	Pass or Fail	Points Awarded
**Side to Side**		/5
**Up and Down**		/5
**Total**		/10

Single Hop Test

	Right	Left		
**Trial 1**	cm	cm		
**Trial 2**	cm	cm	LSI	Points
**Mean**	cm	cm	%	/5

PHASE 5 RETURN TO SPORT

Part D: Functional Testing

Triple Hop Test

	Right	Left	LSI	Points
**Trial 1**	cm	cm		
**Trial 2**	cm	cm		
**Mean**	cm	cm	%	/5

Triple Cross Over Hop Test

	Right	Left	LSI	Points
**Trial 1**	cm	cm		
**Trial 2**	cm	cm		
**Mean**	cm	cm	%	/5

Side Hop Test

	Right	Left	LSI	Points
**Trial 1**	reps	reps	%	/5

Single Leg Rise Test (90° knee flexion)

	Right	Left	LSI	Points
**Trial 1**			%	/5

The single leg rise to fatigue test: Subjects are seated on the edge of a treatment plinth with hips and knees at 90. Arms are to be crossed over the chest. On one leg, subjects are asked to raise to a fully extended knee as many times as possible at a tempo of 2 seconds up, and 2 seconds down. The test is complete when subjects are unable to complete any further squats, or the tempo or form is incorrect. The maximum number of squats is recorded for each leg.

PHASE 5 RETURN TO SPORT

Part D: Functional Testing

Limb Symmetry Index (LSI) Scoring

The Limb Symmetry Index is calculated by dividing the mean distance (cms), or repetitions of the involved limb by the means of the non-involved limb, and multiply by 100. For the tests that use the limb symmetry index, the following criteria will apply:

Limb Symmetry Index (dominant leg)	Points Awarded	Limb Symmetry Index (non dominant leg)	Points Awarded
97-105	10/10 or 5/5	95-103	10/10 or 5/5
90-96 / 105-110	8/10 or 4/5	85-94 / 103-110	8/10 or 4/5
80-89 / 110-120	6/10 or 3/5	75-84 / 110-120	6/10 or 3/5
70-79 / 120-130	4/10 or 2/5	65-74 / 120-130	4/10 or 2/5
60-69 /130-140	2/10 or 1/5	55-64 / 130-140	2/10 or 1/5
□ 60 / 140+	0 points	□ 55 / 140+	0 points

Part E: General Fitness Testing

Two sports-specific fitness tests that have previously been performed before the ACL injury are to be selected. Such tests could include:

*Beep Test/Shuttle/Yo-Yo Test *Timed Run *Agility T-Test *Sprint Test *Illinois Agility Test

*Bruce Protocol *Other

The purpose of running these fitness tests is to ensure people have regained sufficient fitness to allow full return to game play. For both tests, the athlete must attain the same or better result as pre-injury testing. It's pass or fail. If no fitness tests have been previously performed, or baseline data is unavailable, it is suggested that the clinician and athlete discuss two appropriate tests with the coach (and/or fitness staff if available) and acceptable results are mutually agreed.

General Fitness Test

	Results	Pass or Fail
**Test 1**		
**Test 2**		

It is suggested that these two fitness tests are performed prior to undertaking Part F:

Part F: Functional Testing in a Fatigued State

Four hopping tests are to be performed in a fatigued state. Athletes are to undertake sports-specific exercise or game/match play until they reach a general fatigue level of **7 over 10 on the VAS scale**. When athletes have reached this level of general fatigue, the following tests are to be performed in reasonably quick time.

Single Hop Test

	Right	Left	LSI	Points
**Trial 1**	cm	cm		
**Trial 2**	cm	cm		
**Mean**	cm	cm	%	/5

Triple Hop Test

	Right	Left	LSI	Points
**Trial 1**	cm	cm		
**Trial 2**	cm	cm		
**Mean**	cm	cm	%	/5

Triple Cross Over Hop Test

	Right	Left	LSI	Points
**Trial 1**	cm	cm		
**Trial 2**	cm	cm		
**Mean**	cm	cm	%	/5

Side Hop Test

	Right	Left	LSI	Points

REFERENCES

1. Andrade, R., Pereira, R., Cingel, R., Staal, J. B., & Espregueira-Mendes, J. (2019). How should clinicians rehabilitate patients after ACL reconstruction? A systematic review of clinical practice guidelines (CPGs) with a focus on quality appraisal (AGREE II). *British Journal of Sports Medicine*, *54*, 1-9. https://doi.org/10.1136/bjsports-2018-100310
2. Aspetar. (2023). *Aspetar ACL Rehabilitation Protocol*. Aspetar. https://www.aspetar.com/en/acl-rehabilitation-protocol
3. Berk, A. N., Piasecki, D. P., Fleischli, J. E., Trofa, D. P., & Saltzman, B. M. (2023). Trends in Patient-Reported Outcomes After Anterior Cruciate Ligament Reconstruction: A Systematic Review. *Orthop J Sports Med*, *11*(5), 23259671231174472. https://doi.org/10.1177/23259671231174472
4. Buckthorpe, M., Gokeler, A., Herrington, L., Hughes, M., Grassi, A., Wadey, R., Patterson, S., Compagnin, A., La Rosa, G., & Della Villa, F. (2024). Optimising the Early-Stage Rehabilitation Process Post-ACL Reconstruction. *Sports Med*, *54*(1), 49-72. https://doi.org/10.1007/s40279-023-01934-w
5. Forelli, F., Barbar, W., Kersante, G., Vandebrouck, A., Duffiet, P., Ratte, L., Hewett, T. E., & Rambaud, A. J. M. (2023). Evaluation of Muscle Strength and Graft Laxity With Early Open Kinetic Chain Exercise After ACL Reconstruction: A Cohort Study. *Orthopaedic Journal of Sports Medicine*, *11*(6), 23259671231177594. https://doi.org/10.1177/23259671231177594
6. Guan, Y., Bredin, S. S. D., Taunton, J., Jiang, Q., Wu, N., Warburton, D. E. R., Rauch, B., & Lip, G. Y. H. (2022). Association between Inter-Limb Asymmetries in Lower-Limb Functional Performance and Sport Injury: A Systematic Review of Prospective Cohort Studies. *Journal of Clinical Medicine*, *11*(2). https://doi.org/10.3390/jcm11020360
7. Kuenze, C., Weaver, A., Grindstaff, T. L., Ulman, S., Norte, G. E., Roman, D. P., Giampetruzzi, N., Lisee, C. M., Birchmeier, T., Triplett, A., Farmer, B., Hopper, H., Sherman, D. A., Ness, B. M., Collins, K., Walaszek, M., Baez, S. E., Harkey, M. S., Tulchin-Francis, K., . . . Hart, J. M. (2023). Age-, Sex-, and Graft-Specific Reference Values From 783 Adolescent Patients at 5 to 7 Months After ACL Reconstruction: IKDC, Pedi-IKDC, KOOS, ACL-RSI, Single-Leg Hop, and Thigh Strength. *J Orthop Sports Phys Ther*, *53*(4), 1-8. https://doi.org/10.2519/jospt.2023.11389
8. Ohji, S., Aizawa, J., Hirohata, K., Ohmi, T., Mitomo, S., Koga, H., & Yagishita, K. (2022). Changes in subjective knee function and psychological status from preoperation to 6 months post anterior cruciate ligament reconstruction. *Journal of Experimental Orthopaedics*, *9*(1), 114. https://doi.org/10.1186/s40634-022-00551-2
9. Roula, K., Vasileios, K., Enda, K., Olivia, B., Dustin, M., Michail, P., Andreas, B., Julius, L., Jan, W., & Rodney, W. (2023). Aspetar clinical practice guideline on rehabilitation after anterior cruciate ligament reconstruction. *British Journal of Sports Medicine*, *57*(9), 500. https://doi.org/10.1136/bjsports-2022-106158
10. Sonesson, S., & Kvist, J. (2022). Rehabilitation after ACL injury and reconstruction from the patients' perspective. *Physical Therapy in Sport*, *53*, 158-165. https://doi.org/https://doi.org/10.1016/j.ptsp.2021.10.001
11. Svantesson, E., Hamrin Senorski, E., Webster, K. E., Karlsson, J., Diermeier, T., Rothrauff, B. B., Meredith, S. J., Rauer, T., Irrgang, J. J., Spindler, K. P., Ma, C. B., Musahl, V., The Panther Symposium Acl Injury Clinical Outcomes Consensus, G., Fu, F. H., Ayeni, O. R., Della Villa, F., Della Villa, S., Dye, S., Ferretti, M., . . . Hao Zheng, M. (2020). Clinical Outcomes After Anterior Cruciate Ligament Injury: Panther Symposium ACL Injury Clinical Outcomes Consensus Group. *Orthop J Sports Med*, *8*(7), 2325967120934751. https://doi.org/10.1177/2325967120934751
12. Webster, K. E., & Feller, J. A. (2021). Evaluation of the Responsiveness of the Anterior Cruciate Ligament Return to Sport After Injury (ACL-RSI) Scale. *Orthop J Sports Med*, *9*(8), 23259671211031240. https://doi.org/10.1177/23259671211031240
