# Supplementary material for: Effect of upper limb isometric training (ULIT) on hamstring strength in early postoperative anterior cruciate ligament reconstruction patients: Study protocol for a randomized controlled trial
Source: PLoS One. 2025 Aug 21;20(8):e0319724. doi: 10.1371/journal.pone.0319724 (PMC12370102; doi:10.1371/journal.pone.0319724)
Supplement: S7 Appendix — (PDF) [file pone.0319724.s007.pdf]

S7 Appendix. Adverse Events Form

Adverse Events Form

**Study Site: HCTM**	**Study Name: EFFECT OF UPPER LIMB ISOMETRIC TRAINING ON HAMSTRING STRENGTH IN EARLY POSTOPERATIVE ANTERIOR CRUCIATE LIGAMENT RECONSTRUCTION PATIENTS: A RANDOMISED CONTROLLED TRIAL**
**Subject ID:**	**Ethical Review Board: REC UKM**
	**Principal Investigator:**

Has the participant had any Adverse Events during the study? **Yes ☐ No ☐** ***(If yes, please list all Adverse Events below)***

Severity	Study Intervention Relationship	Action Taken Regarding Study Intervention	Outcome of AE	Expected	Serious
1 = Mild<br>2 = Moderate<br>3 = Severe	1 = Definitely related<br>2 = Possibly related<br>3 = Not related	1 = None<br>2 = Discontinued permanently<br>3 = Discontinued temporarily<br>4 = Reduced Dose<br>5 = Increased Dose<br>6 = Delayed Dose	1 = Resolved, No Sequelae<br>2 = AE still present- no treatment<br>3 = AE still present-being treated<br>4 = Residual effects present-not treated<br>5 = Residual effects present- treated<br>6 = Death<br>7 = Unknown	1 = Yes<br>2 = No	1 = Yes<br>2 = No<br>(If yes, complete SAE form)

No.	Adverse Event	Start Date	Stop Date	Severity	Relationship to Study Intervention	Action Taken	Outcome of AE	Expected?	Serious Adverse Event?	I[illegible]
