# Supplementary material for: Effect of upper limb isometric training (ULIT) on hamstring strength in early postoperative anterior cruciate ligament reconstruction patients: Study protocol for a randomized controlled trial
Source: PLoS One. 2025 Aug 21;20(8):e0319724. doi: 10.1371/journal.pone.0319724 (PMC12370102; doi:10.1371/journal.pone.0319724)
Supplement: S9 Appendix — (PDF) [file pone.0319724.s009.pdf]

S9 Appendix. Study Ethics Approval

UNIVERSITI KEBANGSAAN MALAYSIA
The National University of Malaysia

SEKRETARIAT ETIKA PENYELIDIKAN UKM • UKM RESEARCH ETHICS SECRETARIAT

Reference: UKM PPI/111/8
Date : 29 November 2024

Associate Professor Dato' Dr. Badrul Akmal Hisham Md Yusoff
Department of Orthopaedics & Traumatology
Faculty of Medicine UKM

Dear Professor/Datuk/Dato'/Datin/Sir/Madam,

ETHICAL APPROVAL TO CONDUCT RESEARCH IN THE NATIONAL UNIVERSITY OF MALAYSIA

Research title	:	Effect Of Upper Limb Isometric Training On Hamstring Strength In Early Postoperative Anterior Cruciate Ligament Reconstruction: A Randomised Controlled Trial
Ethics Ref. No.	:	JEP-2024-860
Approval period	:	24 November 2024 – 23 November 2026
Study Site	:	Hospital Canselor Tuanku Muhriz
Sample Size	:	32 Subjects

With reference to the above.

2. The Research Ethics Committee, The National University of Malaysia (RECUKM) has provided ethical approval for above study. Please be reminded permission from the Deputy Dean of research of the faculty or Director of Institute/Centre and all relevant heads of departments / units where the study will be carried out must be obtained prior to the study. You are required to follow and comply with their decision and all other relevant regulations.

3. Please note that the investigator's responsibility is to ensure that:

i. All Adverse Events should be reported to RECUKM as soon as possible.
ii. Progress report should be submitted every **6 Months.**
iii. All changes / amendments to the study documents / study sites / study team must be notified to the RECUKM. All changes must be approved by RECUKM before continuation of the study.
iv. Application for renewal of the approval has to be submitted to RECUKM within one month (1 month) prior to the expiry of ethical approval.
v. Final Report should be provided to RECUKM when the project is complete.
vi. Please take note that all records and data are to be kept strictly CONFIDENTIAL and can only be used for the purpose of this study. All precautions are to be taken to maintain data confidentiality.

Required forms can be obtained from the The Research Ethics Committee, The National University of Malaysia (RECUKM) website: **https://www.ukm.my/jepukm**

Thank you.

Yours sincerely,

PROFESOR DR MOHD SHAHRIR MOHAMED SAID
Chairman
Research Ethics Committee
The National University Of Malaysia

c.c. - **Director**
Hospital Canselor Tuanku Muhriz

- **Deputy Dean (Research & Innovation)**
Faculty of Medicine, UKM

- **Head of Department Orthopaedics & Traumatology**
Faculty of Medicine UKM

- **Associate Professor Dr. Ashril Yusof**
Faculty of Sports and Exercise Science
University of Malaya

- **Associate Professor Dr. Mohamad Shariff A Hamid**
Faculty of Medicine
University of Malaya

- **Dr. Norlelawati Mohamad**
- **Dr. Muhamad Karbela Reza Ramlan**
Department of Orthopaedics
Hospital Canselor Tuanku Muhriz
UKM Medical Centre

- **Mr. Razlan Alkhudri Ismail**
Department of Medical Rehabilitation Services
Hospital Canselor Tuanku Muhriz
UKM Medical Centre

- **Mr. Khairul Hazdi Yusof**
Unit Pengurusan Risiko
Hospital Canselor Tuanku Muhriz
UKM Medical Centre

- **Efri Noor Muhamad Hendri (K015643 – PhD Candidate)**
Department of Orthopaedics & Traumatology
Faculty of Medicine UKM

- Approval letter File 2024

SEKRETARIAT ETIKA PENYELIDIKAN UKM • UKM RESEARCH ETHICS SECRETARIAT

**NAME OF ETHICS COMMITTEE/IRB:**<br>Research Ethics Committee,<br>The National University of Malaysia	**ETHICS COMMITTEE/IRB REF NO :**<br>**JEP-2024-860**

PROTOCOL TITLE:
Effect Of Upper Limb Isometric Training On Hamstring Strength In Early Postoperative Anterior Cruciate Ligament Reconstruction: A Randomised Controlled Trial

PRINCIPAL INVESTIGATOR:
Associate Professor Dato' Dr. Badrul Akmal Hisham Md Yusoff
Department of Orthopaedics & Traumatology
Faculty of Medicine UKM

The following items [X] have been received and reviewed in connection with the above study to be conducted by the above investigator.

Documents

- [X] Research Application Form (UKM-JEP-BO01)
- [X] Review Form (UKM-JEP-BO02)
- [X] Non-Disclosure Agreement (UKM-JEP-BO03)
- [X] Conflict Of Interest Form
- [X] Research Proposal / Protocol
- [X] Participant Information Sheet:
  - [X] Malay [X] English [ ] Others :
- [X] Participant Consent Form:
  - [X] Malay [X] English [ ] Others :
- [ ] Questionnaire:
  - [ ] Malay [ ] English [ ] Others :
- [X] Curriculum Vitae of Researchers:
  - [X] Principal [X] Co-researcher [X] Student
- [X] Good Clinical Practice Certificate (GCP)
- [X] Project Agreement

The Research Ethics Committee, The National University of Malaysia operates in accordance to the International Conference of Harmonization Good Clinical Practice Guidelines.

Comments (if any): ______________________________

Date of Approval: 24 November 2024

PROFESSOR DR. MOHD SHAHRIR MOHAMED SAID
Chairman
Research Ethics Committee
The National University of Malaysia
